# Genome-enabled insights into the biology of thrips as crop pests

**DOI:** 10.1186/s12915-020-00862-9

**Published:** 2020-10-19

**Authors:** Dorith Rotenberg, Aaron A. Baumann, Sulley Ben-Mahmoud, Olivier Christiaens, Wannes Dermauw, Panagiotis Ioannidis, Chris G. C. Jacobs, Iris M. Vargas Jentzsch, Jonathan E. Oliver, Monica F. Poelchau, Swapna Priya Rajarapu, Derek J. Schneweis, Simon Snoeck, Clauvis N. T. Taning, Dong Wei, Shirani M. K. Widana Gamage, Daniel S. T. Hughes, Shwetha C. Murali, Samuel T. Bailey, Nicolas E. Bejerman, Christopher J. Holmes, Emily C. Jennings, Andrew J. Rosendale, Andrew Rosselot, Kaylee Hervey, Brandi A. Schneweis, Sammy Cheng, Christopher Childers, Felipe A. Simão, Ralf G. Dietzgen, Hsu Chao, Huyen Dinh, Harsha Vardhan Doddapaneni, Shannon Dugan, Yi Han, Sandra L. Lee, Donna M. Muzny, Jiaxin Qu, Kim C. Worley, Joshua B. Benoit, Markus Friedrich, Jeffery W. Jones, Kristen A. Panfilio, Yoonseong Park, Hugh M. Robertson, Guy Smagghe, Diane E. Ullman, Maurijn van der Zee, Thomas Van Leeuwen, Jan A. Veenstra, Robert M. Waterhouse, Matthew T. Weirauch, John H. Werren, Anna E. Whitfield, Evgeny M. Zdobnov, Richard A. Gibbs, Stephen Richards

**Affiliations:** 1 Department of Entomology and Plant Pathology, North Carolina State University, Raleigh, NC 27695 USA; 2 Virology Section, College of Veterinary Medicine, University of Tennessee, A239 VTH, 2407 River Drive, Knoxville, TN 37996 USA; 3 Department of Entomology and Nematology, University of California Davis, Davis, CA 95616 USA; 4 Laboratory of Agrozoology, Department of Plants and Crops, Ghent University, Coupure Links 653, 9000 Ghent, Belgium; 5 Institute of Molecular Biology and Biotechnology, Foundation for Research and Technology-Hellas, Vassilika Vouton, 70013 Heraklion, Greece; 6 Department of Genetic Medicine and Development, University of Geneva Medical School, and Swiss Institute of Bioinformatics, Geneva, Switzerland; 7 Institute of Biology, Leiden University, 2333 BE Leiden, The Netherlands; 8 Institute for Zoology: Developmental Biology, University of Cologne, 50674 Cologne, Germany; 9 Department of Plant Pathology, University of Georgia - Tifton Campus, Tifton, GA 31793-5737 USA; 10 National Agricultural Library, USDA-ARS, Beltsville, MD 20705 USA; 11 Department of Plant Pathology, Kansas State University, Manhattan, KS 66506 USA; 12 Department of Biology, University of Washington, Seattle, WA 98105 USA; 13 Chongqing Key Laboratory of Entomology and Pest Control Engineering, College of Plant Protection, Southwest University, Chongqing, China; 14 International Joint Laboratory of China-Belgium on Sustainable Crop Pest Control, Academy of Agricultural Sciences, Southwest University, Chongqing, China and Ghent University, Ghent, Belgium; 15 Department of Botany, University of Ruhuna, Matara, Sri Lanka; 16 Human Genome Sequencing Center, Department of Human and Molecular Genetics, Baylor College of Medicine, One Baylor Plaza, Houston, TX 77030 USA; 17 Department of Biological Sciences, University of Cincinnati, Cincinnati, OH 45221 USA; 18 IPAVE-CIAP-INTA, 5020 Cordoba, Argentina; 19 Department of Biology, Mount St. Joseph University, Cincinnati, OH 45233 USA; 20 Department of Biology, University of Rochester, Rochester, NY 14627 USA; 21 Queensland Alliance for Agriculture and Food Innovation, The University of Queensland, St. Lucia, QLD 4072 Australia; 22 Department of Biological Sciences, Wayne State University, Detroit, MI 48202 USA; 23 School of Life Sciences, University of Warwick, Gibbet Hill Campus, Coventry, CV4 7AL UK; 24 Department of Entomology, Kansas State University, Manhattan, KS 66506 USA; 25 Department of Entomology, University of Illinois at Urbana-Champaign, Urbana, IL 61801 USA; 26 INCIA UMR 5287 CNRS, University of Bordeaux, Pessac, France; 27 Department of Ecology and Evolution, Swiss Institute of Bioinformatics, University of Lausanne, 1015 Lausanne, Switzerland; 28 Center for Autoimmune Genomics and Etiology, Divisions of Biomedical Informatics and Developmental Biology, Cincinnati Children’s Hospital Medical Center, Cincinnati, OH 45229 USA; 29 Department of Pediatrics, University of Cincinnati, College of Medicine, Cincinnati, OH 45229 USA

**Keywords:** Thysanoptera, Western flower thrips, Hemipteroid assemblage, Insect genomics, Tospovirus, Salivary glands, Chemosensory receptors, Opsins, Detoxification, Innate immunity

## Abstract

**Background:**

The western flower thrips, *Frankliniella occidentalis* (Pergande), is a globally invasive pest and plant virus vector on a wide array of food, fiber, and ornamental crops. The underlying genetic mechanisms of the processes governing thrips pest and vector biology, feeding behaviors, ecology, and insecticide resistance are largely unknown. To address this gap, we present the *F. occidentalis* draft genome assembly and official gene set.

**Results:**

We report on the first genome sequence for any member of the insect order Thysanoptera. Benchmarking Universal Single-Copy Ortholog (BUSCO) assessments of the genome assembly (size = 415.8 Mb, scaffold N50 = 948.9 kb) revealed a relatively complete and well-annotated assembly in comparison to other insect genomes. The genome is unusually GC-rich (50%) compared to other insect genomes to date. The official gene set (OGS v1.0) contains 16,859 genes, of which ~ 10% were manually verified and corrected by our consortium. We focused on manual annotation, phylogenetic, and expression evidence analyses for gene sets centered on primary themes in the life histories and activities of plant-colonizing insects. Highlights include the following: (1) divergent clades and large expansions in genes associated with environmental sensing (chemosensory receptors) and detoxification (CYP4, CYP6, and CCE enzymes) of substances encountered in agricultural environments; (2) a comprehensive set of salivary gland genes supported by enriched expression; (3) apparent absence of members of the IMD innate immune defense pathway; and (4) developmental- and sex-specific expression analyses of genes associated with progression from larvae to adulthood through neometaboly, a distinct form of maturation differing from either incomplete or complete metamorphosis in the Insecta.

**Conclusions:**

Analysis of the *F. occidentalis* genome offers insights into the polyphagous behavior of this insect pest that finds, colonizes, and survives on a widely diverse array of plants. The genomic resources presented here enable a more complete analysis of insect evolution and biology, providing a missing taxon for contemporary insect genomics-based analyses. Our study also offers a genomic benchmark for molecular and evolutionary investigations of other Thysanoptera species.

## Background

**Fig. 1:**
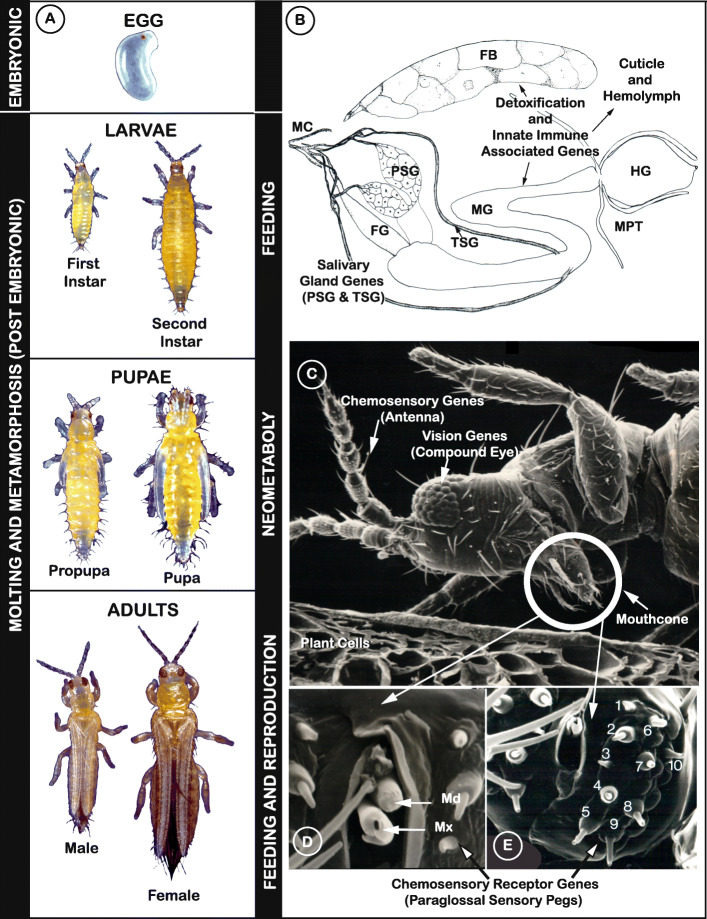
Illustration of how curated gene sets intertwine with understanding of biological processes of *Frankliniella occidentalis*. **a** Developmental stages. Vertical bars: (left) embryonic and postembryonic stages associated with developmental and sex-specific expression analyses of genes underlying molting and metamorphosis through neometaboly; (right) larval and adult stages feed and are associated with divergent clades and expansions in gene families related to host selection and feeding (vision, chemosensation) and detoxification of xenobiotics; propupal and pupal stages do not feed; adults reproduce by arrhenotokous parthenogenesis. Modified from [2], permission of CAB International through PLSclear. **b** Cartoon depicting principal and tubular salivary glands (PSG, TSG) associated with enriched expression of specific genes, and the midgut (MG), hindgut (HG), Malpighian tubules (MPT), and fat body (FB), important sites for detoxification and innate immunity gene sets along with the hemolymph and cuticle. Modified from [3], permission by Elsevier. **c** Scanning electron micrograph (SEM) of adult pre-probing behavior highlights compound eyes used in visual aspects of host finding (associated with opsin genes); external antennal and mouthcone sensory structures essential to host finding, choice, and feeding; likely associated with expanded gene families underlying chemosensation. Internal leaf anatomy shows cells most commonly fed on. Modified from [4], permission from Springer-Verlag. **d** SEM showing the tips of the single mandible (Md) and paired maxillae (Mx) forming the feeding tube. Modified from [5], permission of Elsevier. **e** Mouthcone paraglossal sensory pegs (numbered, left paraglossa)—pegs 1–5, 7–10, are dual function (mechano- and chemosensory), peg 6 is mechanosensory; their location suggests importance in detecting plant surface microtopography and chemistry during host and feeding-site selection and association with divergent and expanded gene families related to environmental sensing. Modified from [5], permission of Elsevier

Thrips are small, polyphagous, and cosmopolitan insects that comprise the order Thysanoptera. Thysanoptera lies within the Paraneoptera, also commonly called the “hemipteroid assemblage” which also includes the orders Hemiptera, Psocoptera, and Phthiraptera. Among the over 7000 reported thrips species classified into nine families with an additional five identified from fossil species [1], the plant-feeders and crop pests are the most well-characterized members of the order due to their agricultural importance. Thysanopterans present a diverse array of biological, structural, and behavioral attributes, but share characteristics that are unique to insects in the order. Among these are fringed wings (Fig. 1a, Adult panel) and a complex mouthcone (Fig. 1b, c) that houses asymmetrical mouthparts composed of three stylets (Fig. 1d). The paired, maxillary stylets interlock when extended during ingestion, forming a single tube, i.e., food canal, that is also thought to serve as a conduit for saliva, while the single, solid-ended mandibular stylet (peg) is used to pierce substrates (its counterpart is resorbed during embryonic development) [6, 7]. All the stylets are innervated, giving thrips control of stylet direction and movement in response to sensory cues [8]. Thrips also have mechano- and chemosensory structures likely governing host finding and choice. The external surface of the mouthcone supports 10 sensory pegs on each paraglossa, nine of which appear to have a dual chemosensory and mechanosensory function (sensory pegs 1–5, 7–10), and one with a mechanosensory function (sensory peg 6) (Fig. 1e). In addition, internally, there are precibarial and cibarial chemosensory structures, likely important in feeding choices [8].

Also remarkable is their postembryonic development, referred to as “remetaboly” [9] and more recently termed “neometabolous” [10] (Fig. 1a). This developmental strategy has been described as intermediate between holo- and hemimetabolous because the two immobile and non-feeding pupal stages (propupae and/or pupae) (Fig. 1a, Pupae panel) undergo significant histolysis and histogenesis, yet the emergent adult body plan largely resembles that of the larva except for the presence of wings and mature reproductive organs (Fig. 1a, Adult panel).

*Frankliniella occidentalis* (suborder Terebrantia, family Thripidae, subfamily Thripinae) is a devastatingly invasive crop pest species with a global geographical distribution and an extraordinarily broad host range, capable of feeding on hundreds of diverse plant species, tissue types, fungi, and other arthropods. Additionally, this species has developed resistance to diverse insecticides with varying modes of action [11–13]. For example, on cotton, there have been 127 cases reported of field-evolved resistance to 19 insecticides belonging to six groups (modes of action) of insecticides [14]. The insect is haplo-diploid, i.e., haploid males arise from unfertilized eggs, while diploid females develop from fertilized eggs [15]. The short reproductive cycle and high fecundity of this species contributes to its success as an invasive species.

In addition to direct damage to plants, *F. occidentalis* and other thrips vectors interact with and transmit diverse types of plant pathogens [16–19], most notoriously orthotospoviruses [20–22], to a wide array of food, fiber, and ornamental crops around the globe. With regard to orthotospovirus-thrips interactions, global expression analyses of whole bodies of *F. occidentalis* [23, 24] and other thrips vectors [25, 26] indicated the occurrence of insect innate immune responses to virus infection. In addition to serving as crop disease vectors, thrips support vertically transmitted, facultative bacterial symbionts that reside in the hindgut [27, 28].

**Fig. 2:**
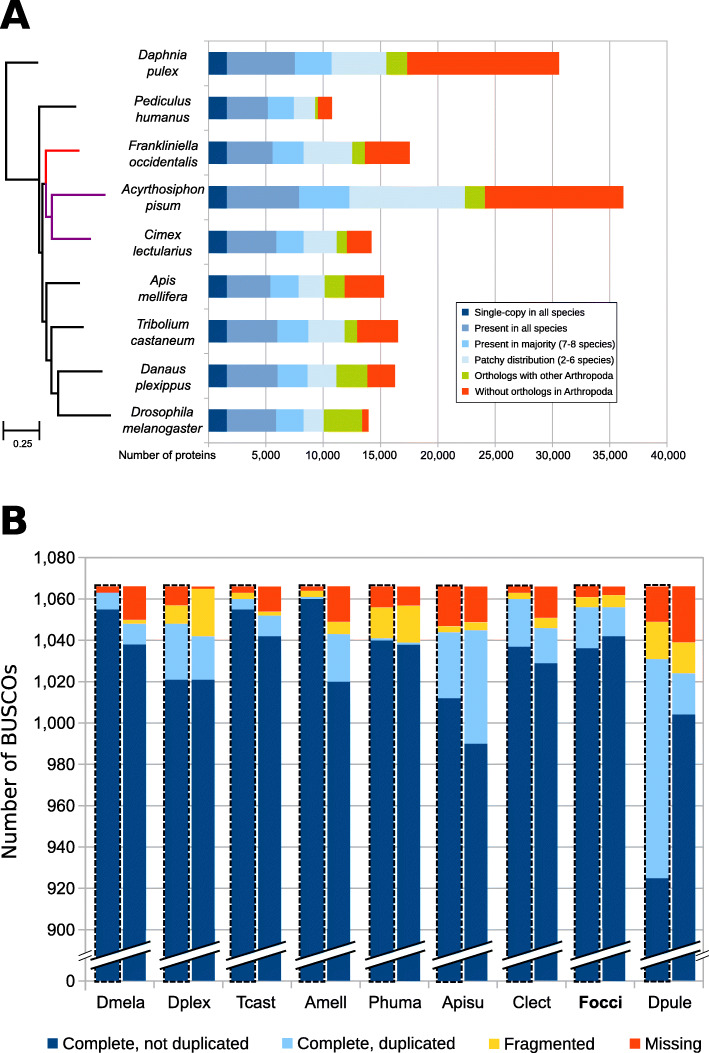
Phylogeny and orthology of *Frankliniella occidentalis* with other arthropods, with genome and gene set completeness assessments. **a** The phylogenomic analysis was based on the aligned amino acid sequences of 1604 single-copy orthologs and placed *F. occidentalis* (shown in red) as basal to the hemipteran species *Acyrthosiphon pisum* and *Cimex lectularius* (shown in purple). All nodes have bootstrap support of 100% and the scale bar corresponds to substitutions per site. OrthoDB orthology delineation with the protein-coding genes from the *F. occidentalis* official gene set identify genes with orthologs in all or most of the representative insects and the outgroup species, *Daphnia pulex*, as well as those with more limited distributions or with no confidently identifiable arthropod orthologs. **b** Assessments using the 1066 arthropod Benchmarking Universal Single-Copy Orthologs (BUSCOs) show few missing genes (5 for the assembly, 4 for the OGS) from *F. occidentalis*, with better OGS completeness than *A. pisum*, *C. lectularius*, and *P. humanus*. The *F. occidentalis* official gene set (OGS) scores better than its genome assembly, indicating that the gene annotation strategy has successfully managed to capture even difficult to annotate genes. The left bars for each species, also outlined with a dashed line, show the results based on the genome, whereas the right bars show the results for the OGSs. Species names abbreviations: Dmela—*Drosophila melanogaster*, Dplex—*Danaus plexippus*, Tcast—*Tribolium castaneum*, Amell—*Apis mellifera*, Phuma—*Pediculus humanus*, Apisu—*Acyrthosiphon pisum*, Clect—*Cimex lectularius*, Focci—*Frankliniella occidentalis*, Dpule—*Daphnia pulex*

**Fig. 3:**
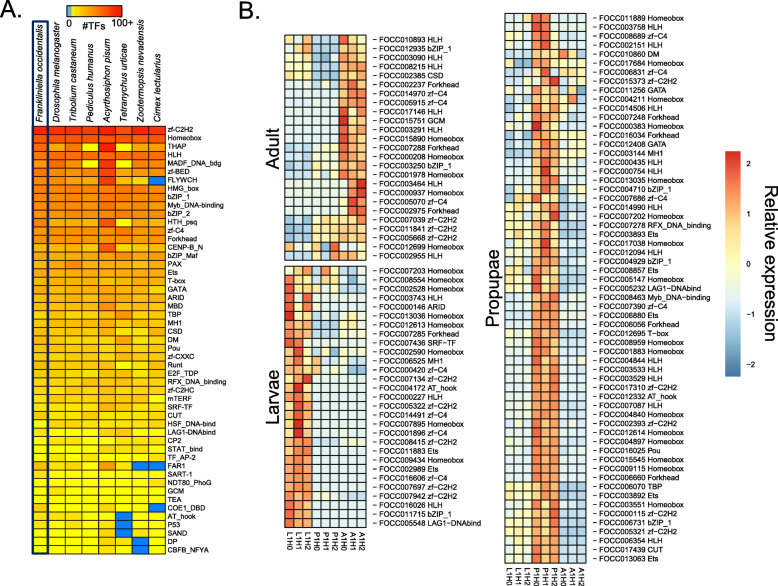
Distribution of transcription factor families across insect genomes and stage-specific expression in *Frankliniella occidentalis.* **a** Heatmap depicting the abundance of transcription factor (TF) families across a collection of insect genomes. Each entry indicates the number of TF genes for the given family in the given genome, based on presence of DNA-binding domains (DBD). Color key is depicted at the top (light blue means the TF family is completely absent)—note log (base 2) scale. Species were hierarchically clustered using average linkage clustering. *F. occidentalis* is boxed. See Additional file 2: Table S5 for TF genes with predicted DBDs. **b** Expression of specific TFs enriched within each developmental stage (larvae, propupae, and adult) based on data presented in Additional file 3. Sample designations: L1 = first-instar larvae, P1 = propupae, and A1 = adults (mixed males and females) of healthy cohorts (H) from three biological replicates (0, 1, 2)

**Fig. 4:**
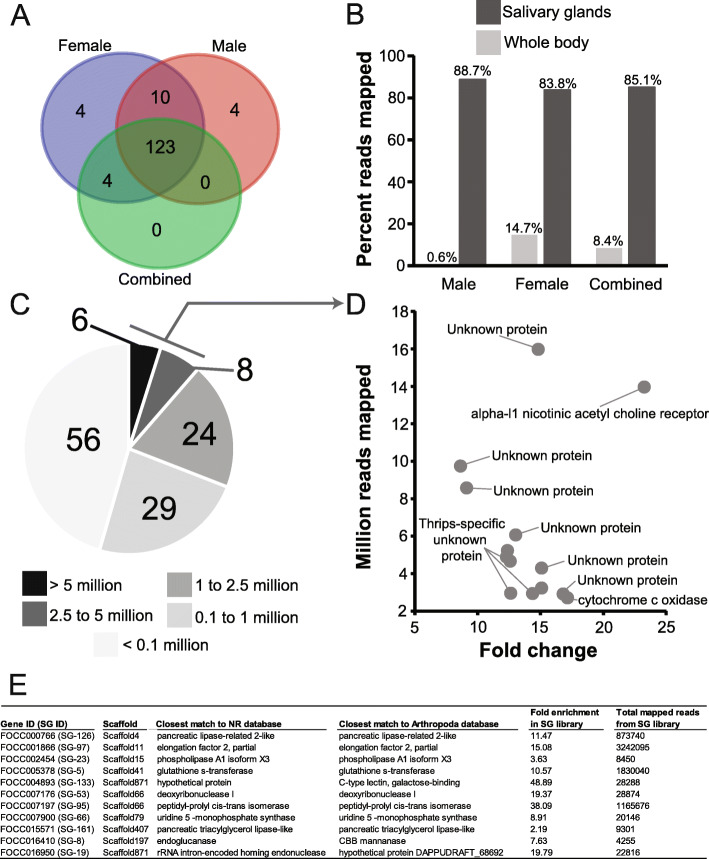
Genes/contigs with enriched expression in the salivary glands of *Frankliniella occidentalis.* RNA-seq reads generated from male and female principal and tubular salivary glands collectively [151] and whole bodies (this study) were used for the enrichment analysis. **a** Venn diagram depicting the overlap in transcript sequences enriched in the salivary glands of males, females, and combined sexes compared respectively to whole bodies. **b** Percent reads from salivary glands and whole-body RNA-seq datasets mapped to the putative 123 salivary gland-associated sequences. **c** Number of reads from the female salivary gland RNA-seq dataset mapping to each of the 123 salivary gland-associated sequences. **d** Reads mapped by fold change for 14 sequences with the highest number of mapped reads denoted in panel **c**. “Thrips-specific unknown protein” signifies hypothetical proteins with no match to proteins in other organisms and “unknown” indicates uncharacterized proteins in other arthropods. Details of expression and potential functions are denoted in Additional file 2: Table S11 (Excerpt D). **e** Specific sequences with functional assignments suggesting they are enzymatic, and based on comparison with other insects systems, could be involved in plant feeding and digestion. Details of expression and potential functions are denoted in Additional file 2: Table S11 (Excerpt E)

While there are numerous studies centered on thrips systematics, feeding behaviors, ecology, virus transmission biology, pest biology and insecticide resistance [29], the underlying genetic mechanisms of the complex and dynamic processes governing these areas of research are largely unknown. Here we present the *F. occidentalis* genome assembly and annotation, with phylogenetic analyses and genome-referenced transcriptome-wide expression data of gene sets centered on primary themes in the life histories and activities of plant-colonizing insects: (1) host-locating and chemical sensory perception (Fig. 1c–e), (2) plant feeding and detoxification (Fig. 1b, c), (3) innate immunity (Fig. 1b), and (4) development and reproduction (Fig. 1a). Analysis of the *F. occidentalis* genome highlights evolutionary divergence and host adaptations of plant-feeding thysanopterans compared to other taxa. Our findings underscore the ability of *F. occidentalis* to sense diverse food sources, to feed on and detoxify an array of natural compounds (e.g., plant secondary compounds) and agrochemicals (e.g., insecticides), and to combat and/or support persistent microbial associations. We also provide insights into thrips development and reproduction. This is the first thysanopteran genome to be sequenced, and the annotations and resources presented herein provide a platform for further analysis and better understanding of not just *F. occidentalis*, but all members of this intriguing insect order.

## Results and discussion

### Genome metrics

The assembly size of the *F. occidentalis* draft genome (Focc_1.0) was determined to be 416 Mb (Table 1), including gaps, which is larger than the published genome size estimate obtained by flow cytometry of propidium iodide-stained nuclei of adult males (337.4 ± 4.3 Mb) and females (345 ± 5 Mb) of *F. occidentalis* (see Table 1 in [31]). The assembly consists of 6263 scaffolds (N50 = 948 kb). One striking feature of the genome is the GC content of ~ 50%, extraordinarily larger than other insects to date [32]. Updated assemblies with reduced proportions of gaps yielded total assembly sizes of 275–278 Mbp (see “Methods”); however, already accumulated manual annotations could not be comprehensively mapped to these new assemblies so the community reverted to using the original assembly.

**Table 1:** Genome metrics of *Frankliniella occidentalis*

Feature	Metric
Assembly size	415.8 Mb (263.8 Mb, contigs only)
Genome coverage	158.7×
Number of contigs	76,021
Contig N50	6.2 kb
Number of scaffolds	6263
Scaffold N50	948.9 kb
GC content	50.9%
^a^Repeat content	9.86%
^b^BUSCO scores	C:99.0%, S:97.6%, D:1.4%, F:0.5%, M:0.6%, *n*:1066
OGS v.1 (# curated)	16,859 genes (1694); 16,902 mRNAs (1738)

^a^Repeat content retrieved from Petersen et al. [30]

^b^BUSCO = Benchmarking Universal Single-Copy Orthologs (Arthropoda gene set = 1066 single-copy genes present in at least 90% of selected representative arthropods); performed on the genome assembly, orthologs classified as complete (C), single (S), duplicate (D), fragmented (F), or missing (M)

### Phylogenomics with a complete and well-annotated genome assembly

Phylogenomic analysis correctly placed *F. occidentalis* (Insecta: Thysanoptera) basal to *Acyrthosiphon pisum* and *Cimex lectularius* (Insecta: Hemiptera) (Fig. 2a). Unexpectedly, however, the body louse *Pediculus humanus* (Insecta: Psocodea) appears as an outgroup to all other insects, which disagrees with previous findings [33]. This discordance is most likely due to taxon sampling and would likely be resolved when more genome sequences become available from early-diverging insect lineages (e.g., Paleoptera). BUSCO assessments (see “Methods”) showed that both the genome assembly (Fig. 2b, left bars, C:99.0%, S:97.6%, D:1.4%, F:0.5%, M:0.6% n:1066) and the official gene set (OGS) (Fig. 2b, right bars, C:99.1%, S:97.6%, D:1.5%, F:0.6%, M:0.4% n:1066) of *F. occidentalis* are very complete when compared to those of other arthropods. Moreover, the OGS-based BUSCO scores are slightly better than the genome-based scores, resulting in reduced numbers of missing BUSCOs. These findings indicate that the *F. occidentalis* gene annotation strategy successfully managed to capture even difficult-to-annotate genes.

### Assembly quality assessment via Hox gene copy number and cluster synteny

The Hox and Iro-C gene clusters that encode homeodomain transcription factors are highly conserved in bilaterian animals and in insects, respectively [34–36], and offer an additional quality appraisal for genome assembly. All single-copy gene models for the expected Hox and Iro-C orthologs were successfully constructed (Additional file 1: Section 1, Table S1.1), and with regard to synteny, we could reconstitute the small Iro-C cluster and partially assemble the larger Hox cluster (Additional file 1: Section 1, Figure S1.1), with linkage for *Hox2/3/4*, *Hox5/6/7*, and *Hox8/9/10*. All linked Hox genes occurred in the expected order and with the expected, shared transcriptional orientation, albeit with some missing coding sequence for some gene models. However, direct concatenation of the four scaffolds with Hox genes would yield a Hox cluster of 5.9 Mb in a genome assembly of 416 Mb, which is disproportionately large (3.5-fold larger relative cluster size compared to the beetle *Tribolium castaneum* and other, de novo insect genomes [37–40]).

Interestingly, although orthology is clear for all ten Hox genes, they are rather divergent compared to other insects. Specifically, several *F. occidentalis* Hox genes have acquired novel introns in what are generally highly conserved gene structures, and several Hox genes encode unusually large proteins compared to their orthologs, corroborating a previous, pilot analysis on unique protein-coding gene properties in this unusually GC-rich genome ([38], see supplement). Global comparisons of structural properties with other insects further confirm that the *F. occidentalis* genome is unusual for the combination of high GC content, large protein sizes, and short exons [41]. It will be interesting to see whether these trends are borne out as genome data become available for more Thysanoptera.

### Genome-wide analysis of transcription factors

In addition to the selected homeodomain proteins, we comprehensively identified likely transcription factors (TFs) among our entire OGS by scanning the amino acid sequences of predicted protein-coding genes for putative DNA-binding domains (DBDs). When possible, we also predicted the DNA-binding specificity of each TF. Using this approach, we discovered 843 putative TFs in the *F. occidentalis* genome, which is similar to other insect genomes (e.g., 701 for *Drosophila melanogaster*). Likewise, the number of members of each *F. occidentalis* TF family is comparable to that of other insects (Fig. 3a). Of the 843 *F. occidentalis* TFs, we were able to infer motifs for 197 (23%) (Additional file 2: Table S5), mostly based on DNA-binding specificity data from *D. melanogaster* (120 TFs), but also from species as distant as human (43 TFs) and mouse (12 TFs). Many of the largest TF families have inferred motifs for a substantial proportion of their TFs, including homeodomain/Hox (64 of 78, 82%), bHLH (30 of 36, 83%), and nuclear receptors (11 of 17, 65%). As expected, the largest gap is for C_2_H_2_ zinc fingers (only 24 of 321, ~ 7%), which evolve quickly by shuffling their many zinc finger arrays, resulting in largely dissimilar DBD sequences (and hence, DNA-binding motifs) across organisms [42]. Weighted gene correlation network analysis (WGCNA) [43] revealed stage-specific patterns in TF expression (Fig. 3b; Additional file 3). For example, Fer3, a basic Helix-Loop-Helix (bHLH) TF—previously linked to reproductive mechanisms [44]—showed increased expression in *F. occidentalis* adults compared to the larvae and propupae. In addition, multiple Hox genes exhibited increased expression in the propupae, which is consistent with their role in morphological development [45].

### Genome-wide search for putative lateral gene transfers (LGTs) of bacterial origin

Once thought to be rare, LGTs from microbes into genomes of arthropods are now considered to be relatively common [46]. Although LGTs are expected to degrade due to mutation and deletion, natural selection can lead to the evolution of functional genes from LGTs, thus expanding the genetic repertoire of the recipient species [47]. We investigated candidate LGTs in *F. occidentalis* using a modification of the pipeline originally developed by Wheeler et al. [48], which has been used to identify LGTs in a number of arthropod species (e.g., [38, 49, 50]).

Three ancient LGT events from different bacterial sources were detected in the *F. occidentalis* genome, involving a levanase, a mannanase, and an O-methyltransferase, with subsequent gene family expansions (Additional file 1: Section 2, Table S2.1, Figures S2.1–S2.3) [24, 28, 38, 48, 51–57]. A PCR-based approach was used to confirm physical linkage between the candidate LGTs and the nearest annotated thrips genes found on the same genomic scaffolds (Additional file 1: Section 2, Table S2.2).

Two of these LGTs show evidence of subsequent evolution into functional thrips genes, based on maintenance of an open reading frame, transcriptional activity, and a signature of purifying selection indicated by reduced levels of non-synonymous to synonymous substitution (Additional file 1: Table S2.1). Both of these are glycoside hydrolases (GHs), which are a large class of proteins involved in carbohydrate metabolism [58]. One is a mannanase (GH5) which was acquired from a *Bacillus* or *Paenibacillus* based on phylogenetic analysis. This gene subsequently underwent expansion into three paralogs in *Frankliniella*. The second ancient LGT is a levanase (GH32) that has undergone duplication subsequent to transfer. The possible origin of this gene is a bacterium in the genus *Streptomyces* or *Massilia*, although the phylogenetic reconstruction precludes a clear resolution of its source. These LGTs could be important in carbohydrate metabolism and therefore impact the feeding ecology of *F. occidentalis*, although their actual functions remain a topic for future study.

The O-methyltransferase LGT is likely derived from a bacterium in the Silvanigrellales or a related proteobacteria in the class Oligoflexia. O-methyltransferases induce the addition of a methyl moiety to small molecules and can affect many biological processes [52]. Subsequent to transfer, the gene has expanded into a three-gene family and two show transcriptional activity based on currently available RNA sequencing data. Whether any of these copies has evolved function in *F. occidentalis* is less clear. There is not strong evidence for purifying selection in any of the paralogs; however, one shows a significant signature of directional selection (Additional file 1: Section 2, Table S2.1).

All three LGT events appear to be ancient. A search of the NCBI transcriptome sequence assembly (TSA) database for Thysanoptera indicates that O-methyltransferase and levanase were acquired prior to divergence of the thrips suborders Terebrantia and Tubulifera approximately 260 MYA [54], while the mannanase was acquired after divergence of the Thripidae and Aeolothripidae approximately 175 MYA. A better understanding of LGT history in thrips will come as additional genomes and more complete phylogenies are available. Further analyses could help to elucidate their functional evolutionary roles in thrips.

### Gene set annotations and analyses

Here we report on the consortium’s analysis of *Frankliniella occidentalis* gene sets and, in select cases, gene expression associated with four primary themes centered on interactions between phytophagous insects, plants, and their environment:

i) Host-locating, sensing, and neural processes;
ii) Plant feeding and detoxification;
iii) Innate immunity, including RNA interference; and
iv) Development and reproduction.

### Host-locating, sensing and neural processes

#### Chemosensory receptors

Chemosensation is important for most insects, including thrips, and the three major gene families of chemoreceptors, the odorant and gustatory receptors (ORs and GRs) in the insect chemoreceptor superfamily [59], and the unrelated ionotropic receptors (IRs) [60], mediate most smell and taste abilities [61]. Chemosensory organs have been described on the antennae of several thrips species, and on the mouthcone, and within the precibarium and cibarium of *F. occidentalis* [5, 8, 62]. Chemosensation plays an important role in the sequence of behaviors involved in host exploration by *F. occidentalis*. This behavioral repertoire includes surface exploration (antennal waving, presumably perceiving olfactory cues; labial dabbing, detecting surface chemistry with paraglossal sensory pegs) (Fig. 1c) and internal exploration (perception of plant fluids with precibarial and cibarial sensilla) (Figure 13 in [8]). The OR, GR, and IR gene families from *F. occidentalis* were compared with those from other representative hemipteroids, specifically the human body louse *P. humanus* [63], the pea aphid *A. pisum* [64], and the bedbug *C. lectularius* [39], as well as conserved representatives from *D. melanogaster* [59, 60] and other insects (Additional file 1: Section 3 [37, 39, 61, 65–107]; Additional file 2: Table S7; Additional file 4). The OR family consists of a highly conserved 1:1 ortholog, the Odorant receptor co-receptor (Orco), found in most insects, including *F. occidentalis* as determined here, plus a variable number of “specific” ORs that bind particular ligands. Comparable to the number reported for *A. pisum* [64], *F. occidentalis* has 84 specific OR genes and all form a divergent clade in phylogenetic analysis of the family (Additional file 1: Figure S3.1), reflecting the generally rapid sequence divergence of ORs in insects and the divergence of thrips from other hemipteroids or Paraneoptera [33]. In addition, *F. occidentalis* has 102 GRs—second to the milkweed bug, *Oncopeltus fasciatus* (115 GR genes) [38] and third in a ranking with other well-curated hemipteran genomes [108]. Phylogenetic analysis of the *F. occidentalis* GRs revealed large expansions within the candidate sugar (18 genes) and carbon dioxide (30 genes) receptor subfamilies (Additional file 1: Figure S3.2). It is unclear how the expansion of sugar receptors might be involved in *Frankliniella* utilization of flowers on host plants, in part because we have yet to fully understand how the eight *Drosophila* sugar receptors [59] are deployed to sense diverse sugars [94]. The large expansion of 30 genes in the carbon dioxide receptor subfamily is comparable to a similar expansion of this subfamily in the dampwood termite *Zootermopsis nevadensis* [109] and the German cockroach *Blattella germanica* [95], but not all are expected to be involved in perception of this gas. The *F. occidentalis* GR repertoire also includes a small expansion of the candidate fructose receptor subfamily to five genes compared to one in other hemipteroids. This subfamily belongs to a distinct lineage of GRs, and in *D. melanogaster*, which have been implicated in detecting “bitter” compounds typically from plants [99]. The remaining 49 GRs, perhaps playing a similar role in detecting “bitter” plant defensive compounds, are highly divergent from those of other hemipteroids. With indication of old and young gene duplications (Additional file 1: Figure S3.2), this group includes a recent expansion of very similar GRs (GR54–67) perhaps involved in sensing host plant chemicals.

The IR family consists of several proteins that are conserved throughout most pterygote insects including the three known co-receptors (Ir8a, 25a, and 76b) and a set of four proteins involved in perception of temperature and humidity (Ir21a, 40a, 68a, and 93a) [102]. Like other hemipteroids and most other insects, *F. occidentalis* has single orthologs of each of these seven genes. This insect species also has eight members of the Ir75 clade that is commonly expanded in insects and involved in perception of acids and amines [103]. The IR family commonly has a set of divergent proteins, some encoded by intron-containing genes, while most are intronless. *F. occidentalis* has one intron-containing gene (Ir101) with relatives in other hemipteroids, and a large divergent clade of 167 IRs including several sets of recently duplicated genes that are encoded by mostly intronless genes (the few with single introns apparently gained them idiosyncratically after expansion of an original intronless gene) (Additional file 1: Figure S3.3). This is a considerable expansion of IRs, with the number of IR genes in *F. occidentalis* being at least five times that reported for other hemipteroids (see Table 2 in [108]). By analogy with the divergent IRs of *D. melanogaster* that appear to function in gustation [106], these genes likely encode gustatory receptors that perhaps mediate perception of host plant chemicals and, hence, host and feeding choices.

There is considerable evidence that chemosensation is important to host, feeding, and oviposition choices made by *F. occidentalis*. For example, *F. occidentalis* detects pheromones and prefers specific plant volatiles [84, 110]. In choice tests with diverse tomato cultivars, adult female *F. occidentalis* preferred fully developed flowers with sepals and petals fully open to those in earlier stages of development and opening, fed preferentially on specific portions of the flower depending on tomato cultivar, and avoided specific acylsugar exudates from Type IV trichomes of tomatoes [111]. Adult females also distinguished between acylsugar molecules, different acylsugar amounts and fatty acid profiles with differentially suppressed oviposition [111–113].

#### Vision genes

In contrast to their uniquely modified wings and mode of flight, thrips are equipped with the canonical pair of lateral compound eyes (Fig. 1c) and three dorsal ocelli, as is typical for winged insects [114]. The success of a multitude of color and light enhanced thrips-trapping devices highlights the importance of vision for host plant recognition in this insect order [115]. For instance, female *F. occidentalis* have been found to exhibit preference for mature host plant flowers over senescent ones during dispersal within a radius of 4 m [116]. In phototaxis assays, *F. occidentalis* displayed conspicuous peak attraction to UV (355 nm) and green (525 nm) light sources in comparison to blue (405, 470 nm), yellow (590 nm), and red (660 nm) [117]. Electroretinogram studies suggested the presence of UV-, blue-, and green-sensitive photopigments in both sexes [117].

Compared to hemipteran genome species studied so far [38, 39, 66], the *F. occidentalis* genome contains a rich repertoire of the opsin G-protein-coupled receptor subfamilies that are expressed in the photoreceptors of the insect compound eye retina. This includes singleton homologs of the UV- and blue (B) opsin subfamilies as well as three homologs of the long wavelength (LW)-opsin subfamily (Additional file 2: Table S8). The latter are closely linked within a 30-k region, indicative of a tandem gene duplication-driven gene family expansion.

Gene tree analysis provided tentative support that the *F. occidentalis* LW opsin cluster expansion occurred independently of the previously reported LW opsin expansions in different hemipteran groups such as water striders, shield bugs, and seed bugs (Additional file 1: Section 4, Fig. S4.1) [38, 66, 67, 108, 118–130]. At the same time, the considerable protein sequence divergence of the three paralogs, which differ at over 140 amino acid sites in each pairwise comparison, indicated a more ancient origin of the cluster, potentially associated with elevated adaptive sequence change. Comparative searches for possible wavelength-sensitivity shifting/tuning substitutions paralleling those identified in the water strider LW opsin paralogs did not produce compelling evidence of candidate changes (not shown) [66]. Understanding the functional significance of the *F. occidentalis* LW opsin gene cluster thus requires future study.

By comparison to the differential deployment of three LW opsins in *Drosophila* [131], it seems likely that one *F. occidentalis* LW opsin paralog is specific to the ocelli, while the remaining two paralogs may be expressed in subsets of the compound eye photoreceptor cells. Overall, the presence of homologs of all three major insect retinal opsin subfamilies correlates well with the previous findings on the visual sensitivities and preferences in this species [117].

The *F. occidentalis* genome also contains singleton homologs of two opsin gene families generally expressed in extraretinal tissues and most often the central nervous system: c-opsin [123] and Rh7 opsin [122]. We failed to detect sequence conservation evidence for Arthropsins, the third extraretinal opsin gene family discovered in arthropods [121], despite the fact that all three extraretinal opsins are present, although at variable consistency, in hemipteran species [38, 66].

#### Neuropeptide signaling

Insect genomes contain large numbers of neuropeptide and protein hormones (> 40), and their receptors, many of which play significant roles in modulating sensory signals and feeding. Neuropeptides are generally encoded by small genes and occasionally evolve rapidly including the loss and duplications of these genes in different evolutionary lineages. While a number of neuropeptides are missing in several insect genomes, the genome of *F. occidentalis* still seems to have a complete set of neuropeptides (Additional file 2: Table S10), including all three allatostatin C-like peptides, which is a rather rare case in insects. Alternatively spliced exons encoding similar, but distinctive, mature peptides are also conserved: mutually exclusive exons of ion transport peptide A and B [132] and orcokinin A and B [133]. Exceptions occurred in natalisin and ACP signaling pathways [134, 135], for which both neuropeptides and the receptors are missing in this species. A surprising finding in this genome is a second corazonin gene that encodes a slightly different version of corazonin [136]. The gene clearly arose from a duplication of the corazonin gene and it has accumulated a substantial number of changes in the sequence (Additional file 5). The duplicated gene encoding the corazonin precursor does not contain disruptive mutations in the open reading frame and its signal peptide is expected to be functional. The transcripts were also confirmed by RNA-seq evidence provided with the genome resources. Together, this evidence collectively suggests that it is unlikely to be a pseudogene.

Similar to the case of conserved gene number, the motif sequences of the putative mature peptides are also well conserved in *F. occidentalis* (Additional file 5). An exception in this case is found in MIP (myoinhibitory peptide or allatostatin B) [137]. While its peptide motif is highly conserved not only in insects but also in mollusks and annelids, in *F. occidentalis*, the C-terminal tryptophan is replaced by a phenylalanine and 23 of the 25 MIP paracopies of the precursor have this unusual sequence. The predicted presence of a disulfide bridge in the N-terminal of the longest pyrokinin is another unusual and noteworthy structural feature.

Receptors associated with the set of *F. occidentalis* neuropeptides and hormones were also cataloged (Additional file 2: Table S9). In *Drosophila*, only a few neuropeptide genes have more than one receptor. However, in the *F. occidentalis* genome, there are duplicate G-protein-coupled receptors (GPCR) for SIFamide, PTH, the CRF-like diuretic hormone 44, and CNMamide. These are ancestral and are generally conserved in other insect species as single copies. What is unusual in the *F. occidentalis* genome is that GPCRs for trissin, vasopressin, leucokinin, and RYamide as well as the orphan GPCR moody all have local duplications, which are likely generated by recent events in this species. These recently duplicated GPCRs include receptors for neuropeptides implicated in water homeostasis: vasopressin, leucokinin, and RYamide [138–140], implying that osmoregulatory processes are tightly regulated in *F. occidentalis*.

### Plant feeding

#### Salivary gland-associated genes

Among piercing-sucking insects, salivation is a key component of their ability to feed on plants. Saliva may form a protective sheath for the stylets, permit intra and intercellular probing, and serve as elicitors that interact with plant defense pathways in ways that may benefit the insect (reviewed in [141, 142]). While little is known about the function of *F. occidentalis* saliva, it is expected to play a key role in this insect’s capacity to feed on an extraordinarily large number of plant species and its ability to transmit viruses (reviewed in [20]). Many insect SG-associated genes, in particular those that encode proteins that elicit or suppress host defenses, are species-specific, are highly divergent, and evolve rapidly [143–146]. Furthermore, arthropod SG transcriptomes and proteomes have unveiled significant proportions of novel proteins, i.e., with no known homology in other, even closely related, species [143, 144]. Among highly polyphagous arthropods (i.e., the spider mite, *Tetranychus urticae*, or the green peach aphid, *Myzus persicae*), transcriptomic analyses revealed an especially large collection of salivary proteins and many genes that lack homology to genes of known function [147–150]. In light of these findings and the highly polyphagous nature of *F. occidentalis*, we used a comprehensive set of putative *F. occidentalis* salivary gland-associated genes and performed comparative analyses of RNA-seq datasets derived from salivary glands (SGs: principal salivary glands and tubular salivary glands, Fig. 1b) [151] relative to the entire body. The analysis revealed 141 and 137 transcript sequences in SGs of *F. occidentalis* females and males, respectively, and 127 in a combined sex analysis that were significantly greater in abundance compared to whole-body expression. There were 123 sequences that overlapped between the three salivary gland sets (Fig. 4a; Additional file 2: Table S11). These 123 sequences represent 83–88% of all reads mapped in salivary gland libraries and only a maximum of 14.7% of the reads from the whole-body samples (Fig. 4b). Many of the SG-enriched sequences (~ 69%) have fewer than one million reads mapped per salivary gland dataset and very few (11%) are highly expressed with over 2.5 million reads mapped per sequence (Fig. 4c). Among the 123 putative SG-enriched genes, fewer than half (41%) match described proteins. The majority (~59%) are either unknown proteins (12%), i.e., matches proteins in other species that are not yet functionally characterized, or *F. occidentalis*-specific (46%), uncharacterized proteins with no significant match to known proteins (Additional file 2: Table S11). Of the 14 highly expressed genes (Fig. 4d), structural prediction analyses revealed that nine are predicted to be extracellular (among these, one has a signal peptide predicting a secreted protein), indicating that these proteins may be saliva components, and one has a predicted transmembrane domain (specific proteins denoted in Additional file 2: Table S11, Excerpt D). At least 11 of the predicted SG-enriched proteins have provisional functions expected to be enzymatic, suggesting they likely have specific roles related to the breakdown of plant materials or response to the host during feeding (Fig. 4e). Among these, five are predicted to be extracellularly localized, one of which has a predicted signal peptide and two are robustly predicted to be secreted proteins based on all three criteria: presence of a signal peptide cleavage site on the N terminus, predicted to be extracellularly localized, and predicted to be transmembrane proteins associated with outer membranes (details regarding function denoted in Additional file 2: Table S11, Excerpt E). One of the proteins predicted to be secreted, the pancreatic tricylglycerol lipase-like gene (FOCC002454, original maker ID: FOCC003652-RA) and three additional thrips-specific proteins with signal peptides were included in validation of enriched expression by real-time quantitative reverse-transcription PCR. Expression analysis confirmed that the predicted SG sequences are either specifically expressed in SGs, or enriched in SGs when compared to thrips heads and bodies (Additional file 1: Section 5, Fig. S5.1) [4, 20, 151–163]. Validation with these genes yielded a Pearson correlation coefficient of 0.845, indicating that the comparative analysis we performed accurately identified putative salivary gland-enriched sequences. The SG gene set will be very valuable in future investigations aimed at understanding the diverse diet of *F. occidentalis*, and the role of saliva as a vehicle for virus inoculation and possibly a means by which the insect manages plant defenses by its many hosts. Prior to the SG-enrichment analysis, other gene models encoding digestive enzymes were annotated as potential SG genes; we therefore consider these likely gut-associated genes (Additional file 2: Table S12).

The thrips genome has enabled identification of SG-enriched transcripts, greatly refining our understanding of the sialotranscriptome of this highly polyphagous insect [151]. The salivary glands of thrips are of particular importance due to their role in extra-oral digestion, defense against host responses, and delivery of viruses to plants. Annotation and analysis of the SG genes revealed a suite of novel thrips genes, some encoding proteins predicted to be secreted extracellularly, thus likely components of the insect saliva, and may play roles in digestion and/or elicitation or suppression of innate plant defenses. Like other polyphagous herbivores studied to date, many of the thrips SG-enriched genes lack homology to genes of known function [147, 149]. Further genomic and functional comparisons between polyphagous and oligophagous thrips will determine whether the high proportion of thrips-specific genes among the SG-enriched genes is related to the thrips wide host range and further enable the identification of genes that play a role in host specificity.

### Detoxification

#### Cytochrome P450s

Cytochrome P450s (CYPs) are a large, ancient superfamily of enzymes identified in all domains of life and are involved in the metabolism of multiple substrates with prominent roles in hormone synthesis and breakdown, development, and detoxification [164, 165]. In agricultural systems, *F. occidentalis* has shown a propensity for developing resistance to insecticides commonly utilized to manage this species, and P450s have been specifically implicated in the detoxification of insecticides by *F. occidentalis* [166, 167]. Within the *F. occidentalis* genome we identified and classified, using CYP nomenclature [168], a relatively large number of P450s—130 CYP gene models (Additional file 2: Table S13) comprising 112 different CYP genes (Additional file 6). There was evidence of CYP gene clusters on some scaffolds as noted to occur in other insect genomes including *D. melanogaster* and *T. castaneum* [169, 170]. The repertoire of *F. occidentalis* P450 genes represents 24 CYP families distributed across four known clans (CYP 2, 3, 4, and mito) (Additional file 1: Section 6.1.3, Table S6.1) [168–178]. As with other insects, gene families in CYP clans 3 and 4 are overrepresented—these families include members frequently associated with the breakdown of toxic plant products and insecticides [166]. Family members belonging to clan 2 and mito, i.e., those associated with the synthesis and turnover of the 20-hydroxyecdysone (20E) and cuticle formation, were also represented in the genome (refer to “Postembryonic development” section below). The majority of annotated *F. occidentalis* P450s showed relatively low amino acid identity to other insect P450s, a common aspect of insect genomes [179]. In fact, of the 24 CYP families represented in the *F. occidentalis* genome, we identified 10 new families (= 40 of the 112 CYP genes) (Additional file 1: Table S13), and therefore we consider these thrips-specific. Of these 40 thrips-specific CYP genes, 90% belong to clan 4, with the remaining members in clan 3, and phylogenetic analysis revealed gene duplications and subsequent expansions in gene family members of these two clans in *F. occidentalis* (Additional file 1: Fig. S6.1). Given the already described importance of P450s in insecticide resistance [166, 167], the prevalence of insecticides in the management of thrips species [166], and the multitude of plant defense compounds encountered during the their phytophagous lifestyle [165], knowledge of the diversity of P450s present within the *F. occidentalis* genome is likely essential for optimizing management of this important agricultural pest. The annotation of these P450 genes will enable future functional studies in *F. occidentalis* related to the detoxification of insecticidal and plant defense compounds.

#### ATP-binding cassette (ABC) transporters and carboxyl/choline esterase (CCE) genes

The ABC protein family is one of the largest protein families and present in all kingdoms of life. The majority functions as primary active transporters, hydrolyzing ATP to transport substrates across membranes. Some ABC proteins, however, are receptors or are involved in translation. The carboxyl/cholinesterase (CCE) enzyme family catalyzes the hydrolysis of carboxylesters and plays a role in many biological processes, such as neuron signaling, development, and detoxification of xenobiotics, including insecticides [180–182]. Forty-five and 50 putative ABC and CCE genes were annotated in the *F. occidentalis* genome, respectively (Additional file 2: Table S15 and S16; Additional file 7). The number of *F. occidentalis* ABC genes is on the lower side among those reported for other insect species (Additional file 1: Section 6.2.2, Table S6.3) [54, 125, 129, 130, 180–203] including *Bemisia tabaci* of the Hemiptera, the sister-group of the Thysanoptera [54]. Nevertheless, we did identify a lineage-specific expansion of ABCH genes within the *F. occidentalis* genome (Additional file 1: Figure S6.6). Lineage-specific arthropod ABCH genes were previously shown to respond to environmental changes or xenobiotic exposure [183, 187, 190] and hence these ABCH genes might have a similar function in *F. occidentalis*. In contrast to ABC genes, the number of *F. occidentalis* CCE genes is among the highest of those identified in any insect species (Additional file 1: Table S6.4). This high number of CCEs is due to a lineage-specific expansion within the dietary/detoxification class of CCEs (Additional file 1: Figure S6.7), and with exception to *Bombyx mori*, it is the largest CCE expansion compared to other orders (Additional file 1: Table S6.4). Future work should confirm whether these 28 *F. occidentalis*-specific CCEs are actually detoxification CCEs and whether the polyphagous nature and/or rapid development of insecticide resistance in *F. occidentalis* [200] might be related to this CCE expansion.

### Innate immunity

#### Canonical signaling pathways

Insects rely on innate immunity to respond to and limit infections by myriad microbes, viruses, and parasites encountered in their environments [204–211]. Here we report the annotation of genes associated with pathogen recognition, signal transduction, and execution of defense in *F. occidentalis*, and support these findings with a comparative analysis of immune-related transcripts in two other thrips vector species, *F. fusca* and *Thrips palmi* [24–26].

In total, 96 innate immune genes were curated from the genome (Additional file 2: Table S17) [38, 39, 129, 197, 212–223]. Toll and JAK-STAT pathway members were well represented, and all but two members of the IMD pathway were located. Based on the number of different pathogen recognition receptors, *F. occidentalis* has a well-developed surveillance system—14 PGRPs and 8 GNBPs—greatly exceeding the number reported for other insects [38, 224]. The broad plant host range and biogeography of this thysanopteran species may have expanded the repertoire of receptors capable of recognizing diverse pathogen and/or microbial-associated molecular patterns in these diverse biomes. Expansion of these surveillance systems could be due to the close contact of pupal stages with the soil environment during their development. Likewise, the melanization pathway encoded by the *F. occidentalis* genome is notably extensive compared to other insect genomes [38, 224]. The melanization pathway is triggered by the binding of pathogen recognition molecules to PGRPs and is the first line of defense in insects. Prophenoloxidase (PPO) and serine proteases are the primary players of the melanization pathway. These primary players are well represented in the *F. occidentalis* genome, with six PPOs and serine proteases, compared to the closest plant feeding hemipteran relatives that have only two PPOs each (*Acyrthosiphon pisum* and *Oncopeltus fasciatus).*

The most striking finding is the absence of the signal transducing molecule IMD, as well as FADD, another death domain-containing protein that acts downstream of IMD to activate transcription of antimicrobial peptides (AMPs) [225] in response to Gram-negative bacteria [205] and viruses [211]. Absence of IMD has also been reported for the hemipteran species *A. pisum*, *Bemisia tabaci*, and *Diaphorina citri* [129, 197, 212, 213, 224]. In *Oncopeltus*, IMD could not be identified by homology searches, but was identified by cloning the gene using degenerate primers [38]. IMD was also reported missing from the bedbug *C. lectularius* [39], but was later found using the *Plautia stali* IMD sequence as a query [214]. These findings in hemipterans illustrate that IMD sequences can be highly divergent and conclusions about their absence using solely a homology-based (in silico analysis) approach should be drawn with care.

It has been suggested for *A. pisum* that its phloem-limited diet and dependence on Gram-negative endosymbionts accounts for a generally reduced immune repertoire and the absence of IMD [129, 215, 224]. This does not seem valid for the polyphagous, mesophyll feeding thrips. In contrast to *A. pisum*, almost all other components of the IMD signaling pathway are present in *Frankliniella*, including two Relish molecules (Additional file 2: Table S17). In conclusion, the apparent absence of IMD in *F. occidentalis* does not seem to suggest a reduced immune repertoire, but rather a different way of mediating the response to Gram-negative bacteria, possibly by Toll signaling components. In *Drosophila*, DAP-type peptidoglycans of Gram-negative bacteria moderately induce Toll signaling [216, 217]. In *Tenebrio molitor*, PGRP-SA recognizes both Gram-positive and Gram-negative bacteria [218]. Extensive cross-reactivity of the Toll and IMD signaling pathway is the currently emerging picture from studies on other insects [214, 219, 220] and might have set the stage for multiple independent IMD losses in evolution [214].

#### Comparative analysis of innate immune transcripts in three thrips vector species

**Fig. 5:**
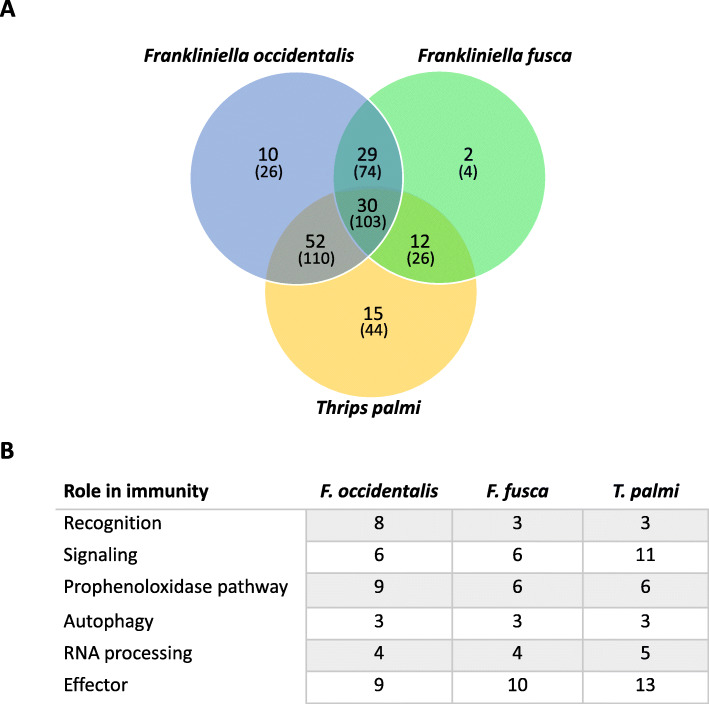
Unique and shared innate immunity-associated transcripts in three thrips vector species of orthotospoviruses. Whole-body, assembled transcriptomes obtained from published orthotospovirus-thrips RNA-seq studies [24–26] were mined for putative innate immune transcripts using an innate immune-associated protein database derived from ImmunoDb (http://cegg.unige.ch/Insecta/immunodb). **a** Venn diagram depicting overlap in orthologous clusters (bold) and transcripts (in parentheses) of innate immune-associated protein sequences in *Frankliniella occidentalis* (tomato spotted with virus), *F. fusca* (tomato spotted wilt virus), and *Thrips palmi* (capsicum chlorosis virus) using Orthovenn.v2. **b** Number of transcripts classified into innate immune categories (roles) and shared across all three vector species. Sequences may fall into more than one category. See Additional file 2: Table S17 and S18, respectively, for innate immune genes and transcript sets; Additional file 8 for Orthovenn outputs

With the apparent absence of IMD and FADD genes in the *F. occidentalis* genome, we used a custom database of innate immune protein sequences to identify a diverse repertoire of transcripts implicating the activities of canonical humoral and cellular innate immunity from a previously assembled transcriptome of *F. occidentalis* adults [24] (Additional file 2: Table S18) [226–234] and similarly for two other known vectors of orthotospoviruses: *F. fusca* [25] and *Thrips palmi* adults [26]. Comparative analysis revealed the occurrence of shared and species-specific innate immune-associated transcripts (Fig. 5; Additional file 8). Both IMD and FADD transcripts were apparently absent (E-value cut-off = 10^− 5^) in all three species which agrees with the annotation of the *F. occidentalis* genome. Relaxing the cut-off (10^− 3^) resulted in weak and ambiguous matches to IMD or IMD-like sequences (Additional file 1: Section 7.4, Table S7.2) [38, 212, 224] of other hemipterans. Absence of transcripts encoding these two canonical genes suggests either cross-reactivity with the other immune signaling pathways or evolution of an atypical signaling pathway which is yet to be deciphered. All components of the JAK/STAT pathway were identified in all three thrips species. There appeared to be an over-representation of sequence matches to cytokine receptors in *F. occidentalis* and *F. fusca*, and while some of these may be involved in innate immunity, they likely play roles in other biological processes as well. Antioxidants, autophagy-related proteins, and inhibitors of apoptosis were well represented among the three transcriptomes. Differences in the number of immune-related transcripts identified between the species should be taken with caution—different biological and experimental factors, including thrips rearing conditions, sampling strategies, and sequencing/assembly parameters may contribute to this variation.

#### Small RNA-mediated gene silencing pathways and auxiliary genes

The RNAi-related gene set examined in this study constitutes a group of genes that are all members of a diverse range of gene (super)families that are evolutionarily unrelated but are linked based on their roles in RNAi [235, 236]. This group includes core machinery genes for the siRNA and miRNA pathways, including several *dicer* and *argonaute* genes, *drosha*, *pasha*, *aubergine*, *loquacious* as well as several genes involved in antiviral immune response and genes encoding auxiliary proteins (*stau*, *maelstrom*, *fmr-1*, *clp-1*, *translin*, *gawky*, *prmt5*, *hen-1*, *p68 RNA helicase*, *ars2*, *egghead*). Also, the gene encoding the transmembrane channel protein *sid1* implicated in cellular uptake of dsRNA was identified in the *F. occidentalis* genome. *F. occidentalis* transmits seven described orthotospovirus species (Order *Bunyavirales*, Family *Tospoviridae*) of economic importance, including tomato spotted wilt virus (TSWV) (reviewed in [22]). These plant-pathogenic viruses are transmitted in a persistent-propagative manner by the thrips vector, i.e., retained through molts, replicating in infected tissues, and inoculative over the lifespan of the adult. In the case of *F. occidentalis*, however, virus infection does not appear to have a negative effect on thrips development or fitness [237, 238]. As RNAi is a potent innate antiviral defense in arthropods, the activities of the core cellular machinery in thrips vectors may be associated with orthotospovirus persistence.

Of the 24 RNAi-related genes queried against the genome, 23 were identified (Additional file 2: Table S19). One gene, *r2d2*, which encodes a co-factor of Dicer-2 and is therefore an element of the siRNA pathway, was not located. This could be due to the absence of *r2d2* in this species, extensive divergence precluding its identification using orthologs, or location in a region of the genome that was not covered by our sequencing. Using pre-existing transcriptome sequence databases for *F. occidentalis*, dsRNA-binding proteins were located; however, they did not match the r2d2 sequences used as queries. For example, in a published *F. occidentalis* EST library of first-instar larvae [239], one sequence (GT302686) was annotated as “tar RNA binding” containing a predicted conserved domain indicative of double-stranded RNA binding (DSRM), matching a staufen-like homolog, while one sequence (contig01752) obtained from a 454 de novo-assembled transcriptome representing mixed stages of *F. occidentalis* matched RISC-loading complex subunit tar RNA-binding proteins [23]. In the *F. occidentalis* genome sequence, one gene coding for an RNA-binding protein similar to *r2d2* was located, but it appeared to encode the very similar protein Loquacious (Loqs) and had a significant match (99.8%) to contig01752. Given their similarity, a phylogenetic tree was constructed with the four isoforms identified to be coded by this gene, clearly confirming that it is indeed the *loqs* homolog (Additional file 1: Section 7.5, Fig. S7.8) [240].

*r2d2* has been reported to be missing in other annotated winged and wingless arthropod genomes and transcriptomes. For example, *r2d2* is missing from the hemipteran *D. citri* [241]. A recent study on the phylogenetic origin and diversification of RNAi genes reported that the gene could not be found in the transcriptomes of any of the wingless insects investigated and did not occur in some older orders of winged insects [240]. Furthermore, *r2d2* also seems to be missing in non-insect arthropods. In the common shrimp *Crangon crangon* for example, no *r2d2* could be found in the transcriptome [235] and data-mining of other Crustacea such as *Daphnia pulex* [240] and *Artemia franciscana* [242] and in the chelicerates *T. urticae* and *Ixodes scapularis* [147, 240] also suggested that *r2d2* is missing in those respective genomes. It has been suggested that in these arthropods and insects, the role of *r2d2* and its interaction with Dicer-2 in the siRNA pathway may have been replaced by Loqs, which serves a similar function, interacting with Dicer-1 in the miRNA pathway. In fact, the involvement of Loqs in the siRNA pathway has been reported in the fruitfly *D. melanogaster*, where four dsRNA-binding proteins interacting with Dicer enzymes have been found, one encoded by the *r2d2* gene and three by the *loqs* gene through alternative splicing. In these fruit flies, Fukunaga and Zamore [243] have shown that one of the Loqs isoforms interacts with Dicer-2 and is involved in siRNA processing. A dual role in both pathways has also been described for Loqs in *Aedes aegypti* [244]. Whether or not this is also the case in non-dipteran insects, such as *F. occidentalis*, or other arthropods is yet to be determined.

#### Antioxidants

Twenty-nine putative proteins in seven families related to antioxidant capacity were identified within the *F. occidentalis* genome (Additional file 2: Table S20). Consequently, the suite of antioxidant proteins identified in *F. occidentalis* was largely as expected, and further investigation into the antioxidant system of *F. occidentalis* will further elucidate the players. The twenty-nine antioxidant response proteins showed high homology to related proteins in other published genomes including *A. pisum*, *Apis mellifera*, *Bombyx mori*, *C. lectularius*, *D. melanogaster*, *P. humanus*, and *T. castaneum*. In most comparisons, homologs in *T. castaneum* showed the highest degree of similarity followed by *A. pisum* and *P. humanus*.

### Development 

#### Embryonic development

The Wnt pathway is a signal transduction pathway with fundamental regulatory roles in embryonic development in all metazoans. The emergence of several gene families of both Wnt ligands and Frizzled receptors allowed the evolution of complex combinatorial interactions with multiple layers of regulation [245]. Wnt signaling affects cell migration and segment polarity as well as segment patterning and addition in most arthropods [246]. Surveying and comparing the gene repertoire of conserved gene families within and between taxonomic groups is the first step towards understanding their function during development and evolution.

Here we curated gene models for the main components of the Wnt signaling pathway in the *F. occidentalis* genome (Additional file 1: Section 8.1, Table S8.1) [37, 38, 247–252] and confirmed their orthology by phylogenetic analysis. We found 9 Wnt ligand subfamilies, three Frizzled transmembrane receptor subfamilies, the co-receptor *arrow*, and the downstream components *armadillo/beta-catenin*, *dishevelled*, *axin*, and *shaggy/ GSK-3*. All of these genes, with the exception of the Frizzled family (three *fz-2* paralogs), were present in single copy in the assembly. Three Wnt genes, *wingless*, *Wnt6*, and *Wnt10*, were linked on the same scaffold, reflecting the ancient arrangement of Wnt genes in Metazoa. One of the Wnt ligands, *Wnt16*, has so far only been reported in the pea aphid *A. pisum* [253], the Russian wheat aphid *Diuraphis noxia* [254], and *O. fasciatus* [38]—adding *F. occidentalis* to this list suggests that the hemipteroid assemblage (clade Acercaria) has retained a Wnt ligand that was subsequently lost within the Holometabola.

#### Postembryonic development

**Fig. 6:**
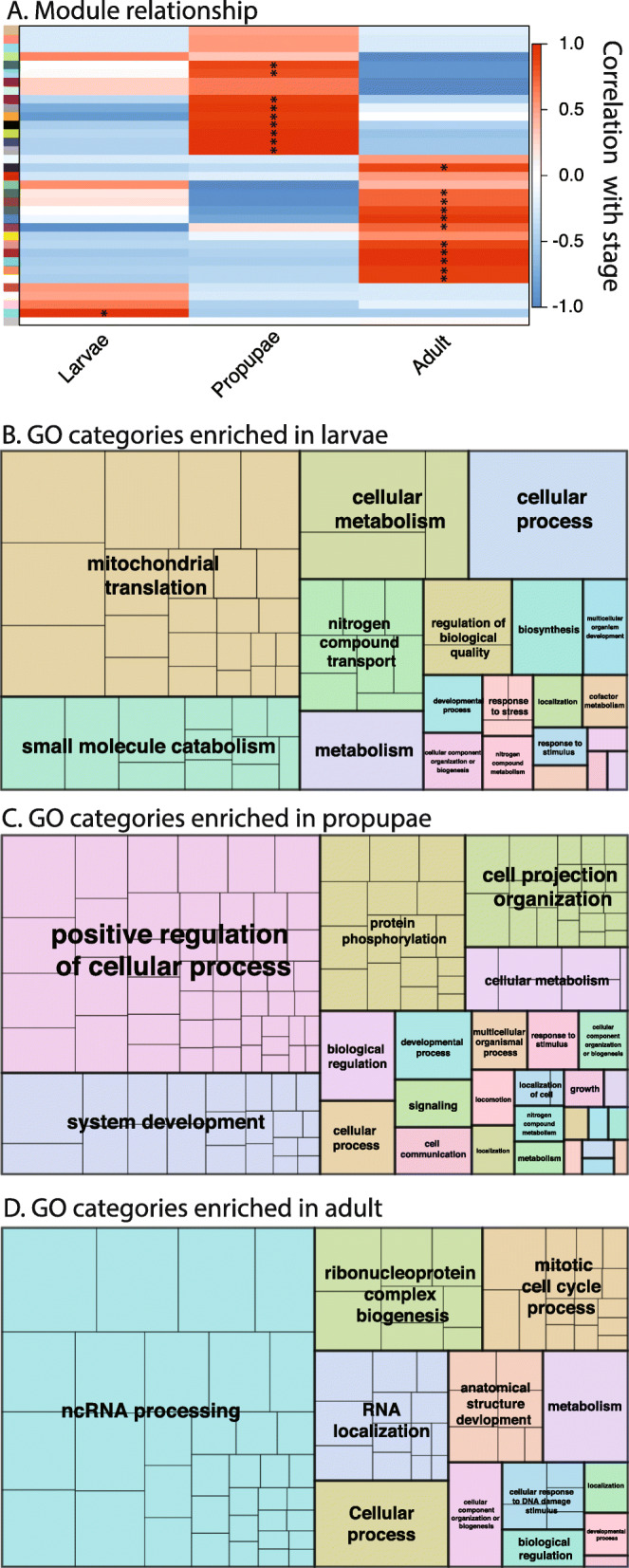
Identification of co-expressed genes (modules) and gene ontologies associated with three developmental stages of *Frankliniella occidentalis*. **a** Association between modules of co-expressed genes (colored boxes stacked on left of figure) and developmental stage, depicting the gene correlation network. Weighted gene co-expression network analysis [43] was performed on a matrix of normalized read counts (FPKM values) obtained from a published *F. occidentalis* RNA-seq study involving three biological replicates of healthy first-instar larvae, propupae, and adults (mixed males and females) [24]. Modules of co-expressed genes were determined by the dynamic tree cutting algorithm with a minimum of 20 genes per module. Modules that exhibited the highest correlation (red color) with a developmental stage are indicated by an asterisk (*). Transcript IDs of co-expressed genes within these significant stage-associated modules are presented in Additional file 3. **b–d** REVIGO (*RE*duce and *V*isualize *G*ene *O*ntologies, [255]) was used to visualize specific GO terms comprised of non-redundant sequences enriched in each developmental stage; sizes of delineated blocks indicate the number of genes within each GO category. Refer to Additional file 3 for more detailed REVIGO maps with identities of each GO term (block) indicated

Neometaboly is an atypical developmental adaptation that emerged independently in a few lineages of Paraneoptera, namely thysanopterans, Aleyrodoidea (whiteflies), and males of Coccomorpha (scales) [10]. Unlike most Hemimetabola, the transition from the penultimate juvenile stage, i.e., the second instar larva of *F. occidentalis,* to the adult stage involves at least one quiescent pupal stage; propupal (P1) and pupal (P2) stages in *F. occidentalis* (see Fig. 1a). These pupal stages mark a period of rapid dissolution of larval structures and dramatic regeneration of the muscle tissues, nervous system, digestive tract, and eyes [15]. Underscoring the morphological transition from larvae to adults, the global network analysis in the present study revealed stage-enriched suites (modules) of co-expressed genes in *F. occidentalis*. There were ~ 2000–3000 stage-associated genes (transcripts) and the network assembled into 35 modules, with one, nine, and 11 modules significantly associated (*P* < 0.05) with L1, P1, and adults, respectively (Fig. 6a, Additional file 3). Enrichment in particular gene ontologies (provisional functions) of the stage-associated transcript sequences exemplifies the biological separation between the three stages. In L1, there was an enrichment of gene ontologies associated with metabolism and growth processes (Fig. 6b), which was similarly reported for nymphal stages for *Oncopeltus fasciatus* [38]. The propupae varied widely from the larvae in that there was significant enrichment in processes associated with systems development, which included anatomical structure development, such as neuron recognition, photoreceptor cell development, and muscle structure, respiratory, and sensory system development—a reflection of the turbulent changes observed during morphogenesis of this non-feeding, quiescent stage [15] (Fig. 6c). Adult-enriched categories implicated genes involved in transcriptional and posttranscriptional regulation of gene expression (coding and non-coding RNA-associated processes, RNA localization and RNP biogenesis), cell division (mitosis), and anatomical structure development (Fig. 6d).

In a more targeted approach, we curated (Additional file 2: Table S21) and developmentally profiled expression (Additional file 2: Table S22) of molting and metamorphosis genes. These included genes associated with the juvenile hormone (JH) and ecdysone and related signaling pathways, as well as insulin signaling and myriad transcription factors associated with the regulation of various developmental processes. Postembryonic development in insects is largely controlled by the action of two developmental hormones, JH and ecdysone. During development, JH action prevents early metamorphosis by blocking the heterochronic expression of certain ecdysone-inducible genes. JH titers maintain the juvenile-juvenile transitions, and when JH titer drops at a developmentally appropriate time, the penultimate larva/nymph develops into the pupal stage (holometabolous) or directly into the adult (hemimetabolous). Ecdysteroids (ecdysone and its derivative, 20-hydroxyecdysone (20E)) control molting at each transition. In the *F. occidentalis* genome, JH and ecdysone pathway genes were determined to be generally conserved. The MEKRE93 pathway [256]—consisting of the JH action transcription factors Met, Kr-h1, and E93—was fully annotated, along with the pupal-specifying gene *Broad*. Together, this gene battery coordinately specifies distinct developmental stages. The antimetamorphic gene Kr-h1 in *F. occidentalis* was previously identified [257], and the published sequence is consistent with the genome annotation. In our dataset, Met expression was associated with L1 as expected for hemi- and holometabolous insects. E93, the specifier for adult development that is thus expected to increase in expression during late nymph or propupae stages [256], was indeed upregulated and enriched in the P1 stage. In contrast, while *Broad* showed low expression in L1 as previously reported [257], expression was exceptionally low in P1—a finding that may be explained by P1 age at time of sampling [257]—and appeared to be associated with the young adult (Additional file 2: Table S22). This finding differs from previous findings for *F. occidentalis* adults [257] and Holometabola [258]. It may be that the broad transcript quantified in our dataset was one of possibly multiple isoforms that play a role in other processes, such as nutritional or steroid signaling associated with reproduction reported for other insects [259], but this remains to be investigated. Three copies of xanthine dehydrogenase (*rosy*), a protein essential in mediating JH action in the developing abdominal epidermis of *D. melanogaster* [260], were identified. Of the three copies associated with *F. occidentalis*, xanthine dehydrogenase-2 was supported by expression data and was relatively more abundant in the adult stage. Finally, both Taiman, the steroid receptor coactivator (*AaFISC* in [261], *TcSRC* in [262]), and FtzF1, which serves as a physical bridge between the JH receptor machinery and ecdysone, were identified with their transcripts upregulated in the P1 stage, during which these two hormones coordinately promote metamorphosis. In *Aedes aegypti*, [263] Ftz-F1 recruits Taiman to the ecdysteroid receptor complex to upregulate 20E-inducible genes with developmental roles [264]. Taiman knockdown in mosquitoes likewise reduces expression of the ecdysone target genes E75A and E74B and impedes ecdysone-driven morphological development [264]. E75A plays a critical role at the onset of metamorphosis [265] and requires Ftz-F1 expression; several E75A enhancers were shown to be occupied by Ftz-F1 [266]. Therefore, Ftz-F1 and Taiman expression during the *F. occidentalis* propupal stage is concordant with hormone-driven developmental reprogramming during transitory pupal development.

Ecdysone-associated genes were identified with varying levels of expression during development. These included 13 ecdysone cascade genes and coactivators (Additional file 2: Table S21) and eight P450 (CYP) “Halloween” family genes, members of P450 clans 2 and mito that catalyze the biosynthesis or inactivation of 20E, were identified (Additional file 2: Table S13; Additional file 1: Section 6.1.3, Table S6.2) [176, 177]. The biosynthesis pathway for 20E includes several conserved P450s [176], and as expected, these evolutionarily conserved developmental CYP genes showed some of the highest amino acid conservation observed among the collection of P450s from the *F. occidentalis* genome versus P450s in other insect genomes. The P450 genes responsible for the synthesis of 20E, i.e., CYP307B1/A2, CYP306A1, CYP302A, CYP315A1, and CYP314A1, were located in the *F. occidentalis* genome (Additional file 1: Table S13), with four of six of these CYP transcripts differentially expressed in L1, P1, and adult stages (Additional file 2: Table S22). CYP18A1, a key enzyme involved in the inactivation of 20E and essential for metamorphosis in *D. melanogaster* [177], was also identified, exhibiting high expression in the P1 stage. Cyp18A1 expression in *Drosophila* was during the prepupal to pupal transition [267], and in *B. mori*, Cyp18A1 was highly expressed in late wandering silk glands through the white prepupal stage [268]. Therefore, Cyp18A1 inactivation of ecdysone via 26-hydroxilation is a conserved phenomenon that precedes pupation across insect taxa and suggests that the propupal of *F. occidentalis* shares transcriptional characteristics of the white prepupal stage in these holometabolous species. In addition to these 20E-associated P450s, two copies of CYP301A1, a conserved gene shown to play a key role in the formation of adult cuticle in *D. melanogaster* [178], were located in the thrips genome in close proximity (on the same scaffold), possibly an indication of a tandem duplication event.

JH and ecdysone titers are tightly regulated via the action of biosynthetic and metabolic genes. Mevalonate kinase, an enzyme in the mevalonate pathway involved in JH biosynthesis in *D. melanogaster* and other insects, was not identified in *F. occidentalis*. However, CYP15A1, a single-copy P450 gene in some insects involved in the synthesis of JH, was located in the genome, and similar to *A. pisum* [269], there are three copies; in the *F. occidentalis* genome, these genes (CYP15A1/P1/P2) occur on different scaffolds (Additional file 2: Table S13). With regard to JH degradation—which is performed by JH epoxide hydrolase (JHEH) and JH esterase (JHE) —a single obvious JHEH gene was identified in contrast to three orthologs in *D. melanogaster* and showed marked upregulation and enrichment in the L1 stage. The *F. occidentalis* genome, however, carries an additional four epoxide hydrolase orthologs, any of which may have JHEH activity—all four showed expression in L1s. Notably, several of the *F. occidentalis* carboxylesterase annotations meet a “diagnostic” criterion (GQSAG motif; A replaced by S in *F. occidentalis*) of functional JHE proteins [270] (Additional file 1: Section 8.2.1, Figure S8.1); however, based on the developmental expression profiles, only one of the putative JHE genes in the *F. occidentalis* genome is predicted as the true JHE (Additional file 2: Table S22). Three *apterous* (Ap) orthologs were identified, apparently the result of tandem duplications. The *apterous* mutation in *Drosophila* results in misregulated JH production, leading to female sterility. In light of this reproductive fitness cost, expression of Ap during *F. occidentalis* larval and adult life—during which JH is necessary for development and reproduction—is expected. In addition to its role in promoting JH synthesis, Ap is a homeodomain protein that establishes dorsoventral boundary in the developing wing disc and *Ap* misexpression has a range of developmental consequences on wing morphology [271]. It is therefore intriguing to ponder a role for *apterous* duplications in the context of thrips’ unique wing morphology.

Many of the annotated postembryonic genes belonged to the bHLH superfamily (Additional file 1: Section 8.2.2), transcription factors that regulate various developmental processes across all domains of life. In *F. occidentalis*, 45 bHLH-PAS/myc family members were conclusively annotated (Additional file 2: Table S21). This gene superfamily showed putative duplication events—three *Enhancer of split* (*E(spl)-bHLH*) paralogs, two *hairy* orthologs, two presumed paralogs of the *dimmed*, and similarly, *knot* (syn. *Collier*) (Additional file 1: Section 8.2.3) [252]—and their expression profiles may indicate stage-specific sub/neofunctionalization (Additional file 1: Table S22).

#### Cuticular proteins

Sequence motifs that are characteristic of several families of cuticle proteins [272] were used to search the genome of *F. occidentalis* for putative cuticle proteins. In total, 101 genes were identified, analyzed with CutProtFam-Pred, a cuticular protein family prediction tool described in Ioannidou et al. [273], and assigned to one of seven families (CPR, CPAP1, CPAP3, CPF, CPCFC, CPLCP, and TWDL) (Additional file 2: Table S23). As with most insects, the CPR RR-1 (soft cuticle), RR-2 (hard cuticle), and unclassifiable types, constituted the largest group of cuticle protein genes in the *F. occidentalis* genome (Additional file 1: Section 9, Table S9.1). The number of genes in the protein families CPR, CPAP1, CPAP3, CPCFC, and CPF were similar to the number in other insects [272]. However, the 10 genes in the TWDL family was greater than that found in most insect orders and is reminiscent of the expansion of this family observed in Diptera (Additional file 1: Section 9, Figure S9.1). Many of the cuticle protein-encoding genes (~ 40%) were arranged in clusters of 3 to 5 genes (Additional file 1, Table S9.2) that were primarily type-specific. However, the sizes of gene clusters were smaller than those observed in other insects, which are typically 3 to ~ 20 genes in size. Additionally, a larger portion (50–70%) of cuticle proteins is typically found in clusters in other insects—clustering of these genes could allow for the coordinated regulation of cuticle proteins and thereby facilitate the development of insecticide resistance.

#### Nuclear receptors

Nuclear receptors (NRs) play important roles in development, reproduction, and cell differentiation in eukaryotes. In insects, many are part of the ecdysteroid signaling cascade. Most of these NRs contain a highly conserved DNA-binding domain (DBD) and a more moderately conserved ligand-binding domain (LBD). These molecules have a very specific working mechanism, being simultaneously a transcription factor and a receptor for small amphiphilic molecules such as steroids, thyroids, vitamins, and fatty acids. In this way, they allow a direct response to certain hormone stimuli by controlling gene expression without requiring a complex cellular signaling cascade. The proteins in this superfamily are categorized into six major subfamilies (NR1-NR6) based on phylogenetic relationships, with an additional subfamily (NR0) containing non-canonical NRs usually lacking either a DBD or LBD [274, 275]. All expected nuclear receptor genes (21 in total) commonly found in insect species were identified in the *F. occidentalis* genome (Additional file 2: Table S24). All known insect members of the NR1-NR6 subfamilies were identified including the NR2E6 and NR1J1 genes that were previously reported to be missing in the hemipteran *A pisum*, the nearest relative to thrips and the first hemimetabolous insect to have its genome sequenced [129, 276]. In the NR0 group, three receptors were identified (Egon, Knirps, and Knirps-like), as was the case with other members of the hemipteroid assemblage (*A. pisum* and *P. humanis*) and *Drosophila*. It is possible that the three NR0 genes found in the *F. occidentalis* genome are orthologous to those in Hemiptera; however, phylogenies of the arthropod NR0 genes are notoriously difficult to resolve due to the lack of semi-conserved LBD and the high divergence between these different NRs.

### Reproduction

Curation and WGCNA of postembryonic developmental genes revealed members of JH, ecdysone, and insulin signaling pathways in *F. occidentalis* that are known to be required in other insects for vitellogenesis, functioning uniquely across taxonomic lines. For instance, ecdysone and JH have opposing functions in reproductive tissue maturation in *Tribolium* and *Drosophila*. In *F. occidentalis*, there were nine adult-stage, co-expressed genes implicated in oocyte development and reproductive biology (Additional file 2: Table S22)—*hydroxymethylglutaryl-CoA synthase 1* and *farnesoic acid O-methyltransferase are* involved in JH biosynthesis, while the others are involved in nutritional (e.g., insulin) and steroid signaling. One oddity that begs further research is the finding that *methoprene tolerant* (Met) was not upregulated in the sampled adult stage of *F. occidentalis*, since this JH receptor has roles in oocyte maturation and vitellogenesis, as well as accessory gland development and function, and in courtship behaviors. Of two lipase-3 like annotations, one was enriched in adults, while the other was enriched in larvae. Larval expression is likely related to nutritional signaling and feeding, whereas the adult transcript is likely required for reproduction.

#### Comparison of reproductive gene expression in male and female thrips

**Fig. 7:**
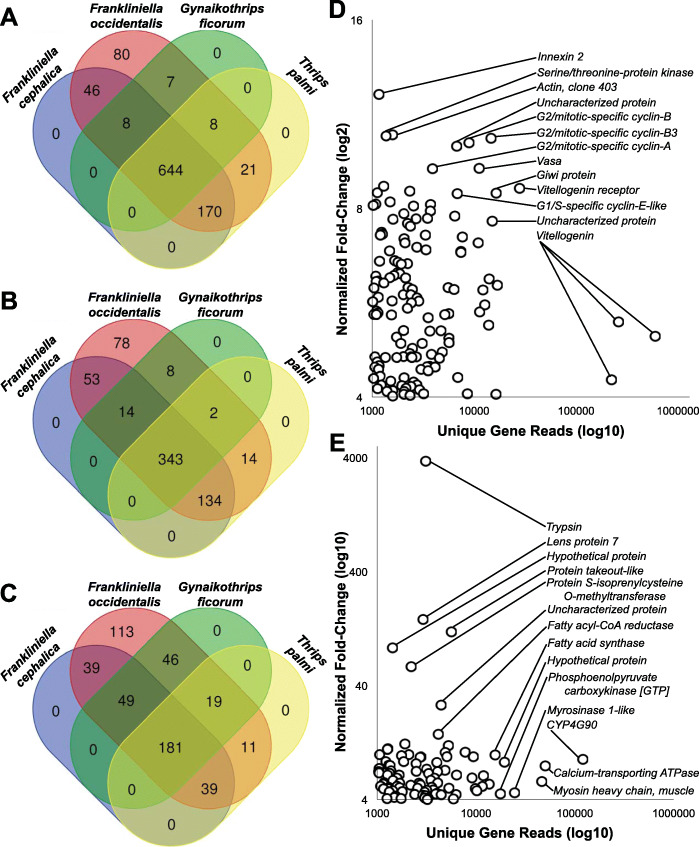
Conserved sex-specific gene expression in thrips. Genome-assembled transcripts derived from RNA-seq reads for females, males, and pre-adults (larval and pupal combined) of *Frankliniella occidentalis* (this study, PRJNA203209) were compared to transcripts generated de novo from publicly available RNA-seq data sets for *Frankliniella cephalica* (PRJNA219559), *Gynaikothrips ficorum* (PRJNA219563), and *Thrips palmi* (PRJNA219609). Venn diagrams depict the number of transcript sequences associated with **a** females, **b** males, and **c** pre-adult stages of thrips. Highly enriched sequences (> 1000 unique reads and > 4-fold difference) conserved in **d** female and **e** male thrips. Open circles in **d** and **e** represent highly enriched gene ontology (GO) terms; especially notable genes are labeled. See methods for enrichment criteria and Additional file 9 for sex-specific genes sets and associated normalized (TPM = transcripts per million) fold change values (relative to the other sex)

To identify male- and female-enriched genes, we performed a comparative RNA-seq analysis between females, males, and larvae (Additional file 9). Following the *F. occidentalis*-specific analysis, specific sets were compared to previous de novo assemblies for other thysanopteran species (Fig. 7). Based on these comparative analyses, 644 female-enriched, 343 male-enriched, and 181 larvae-enriched genes were identified in common among the thrips (Fig. 7a–c). These overlapping sets for females included many factors expected to be increased in this egg generating stage, including vitellogenin and vitellogenin receptors along with other factors associated with oocyte development (Fig. 7d, Additional file 9: Table S1). Males had enriched expression for many factors associated with sperm generation and seminal fluid production (Fig. 7e; Additional file 9: Table S2). Many of these male-associated genes are hypothetical and not characterized, which is common for seminal proteins [277]. One of the male-enriched transcripts included one “myrosinase-like” transcript. Insect-expressed myrosinases have been implicated in alarm pheromone signaling in aphids [278], and the byproduct of its activity (i.e., isothiocyanates) during predation has been shown to act synergistically with the alarm pheromone E-β-farnesene [279]. By analogy to aphids, thrips-expressed myrosinases may serve roles in volatile-mediated communication and aggregation on plants [278]. The larvae datasets were enriched for aspects associated with growth and development, such as cuticle proteins (Additional file 9: Table S3). Overall, these gene expression profiles provide putative male- and female-associated gene sets for future study*.*

### Gene family expansions

Focusing our curation efforts on selected gene sets of relevance to an herbivorous agricultural pest, we identified and characterized expansions of chemosensory receptors (ORs, GRs, IRs), vision genes (opsins), detoxification genes (CYPs, ABCHs, CCEs), innate immunity (PGRPs, GNBPs, PPOs), and cuticle-associated genes (TWDL family cuticle proteins). In order to contextualize these expansions with respect to the entire genome, we examined the outputs from the largest arthropod gene content evolution study to date [280]. Examination of Gene Ontology terms enriched among *F. occidentalis* gene family gains from Thomas et al. [280] revealed independent support for expansions of ORs (odorant binding, olfactory receptor activity), CYPs (oxidoreductase activity, acting on paired donors, with incorporation or reduction of molecular oxygen), ABCHs (transporter activity); PGRPs (peptidoglycan binding), GNBPs (1->3-beta-D-glucan binding), PPOs (monophenol monooxygenase activity), and TWDLs (chitin binding). PGRPs and ABCHs also appear among the 60 families with significantly rapid expansions in *F. occidentalis* [280]. GO terms and annotations of rapidly expanded families point to additional gains in gene families involved in lipopolysaccharide binding (putative toll receptors), inhibition of apoptosis, and chitin binding (cuticle-related). Other families with significantly rapid expansions were mostly of unknown function; however, they include several C2H2 zinc finger families, which in our analysis of transcription factors (Fig. 3), were determined to be the most numerous. In summation, genome-wide gains in gene families associated with chemosensation, detoxification, and innate immunity underscore the adaptive capacity of *F. occidentalis* to invade and thrive in diverse environments utilizing a wide array of plant hosts.

## Conclusions

The *F. occidentalis* genome resources fill a missing taxon in phylogenomic-scale studies of thysanopterans and hemipterans. On the ecological level, the genome will forge new frontiers for thrips genetics and epigenetics studies, genome-wide analyses of biotic and abiotic stress encountered by this pest in diverse environments, a deeper understanding of this insect’s ability to rapidly build pesticide resistance, and identification of genes and gene products associated with plant-microbe/virus-thrips vector interactions. Importantly, the availability of this genome may also provide a means to address the challenge of determining whether *F. occidentalis* is a single widespread, interbreeding gene pool or a series of weakly interbreeding (even non-interbreeding) gene pools (i.e., sibling species) (Mound, personal communication, [29]). From a pest management perspective, the genome provides tools that may accelerate genome-editing for development of innovative new-generation insecticides and population suppression of targeted thrips.

The first look at gene annotations presented here points to unique features underlying the ecological success of this herbivorous pest and plant virus vector, such as the repertoire of salivary gland proteins, the majority of which are thrips-specific. Salivary components play critical roles in insect vector-plant virus interactions, including feeding, modulation of plant defenses, and virus inoculation into new hosts. *Tomato spotted wilt orthotospovirus* progressively invades and replicates in multiple *F. occidentalis* organs, including the SGs from which the virus is inoculated during feeding [152]. This intimate relationship also provides an opportunity for virus infection to modulate gene expression in insect vector SGs, which in turn may regulate insect feeding and plant defenses mediating successful inoculation. It is likely that when virus infection of SGs alters gene expression, whether genes encode proteins that facilitate feeding or mount/suppress defense, plant, insect, and/or virus may accrue substantial benefits. As we attempt to harness host plant defenses against insects and viruses and create more sustainable host plant resistance, knowledge of the *F. occidentalis* salivary protein repertoire provided by this genome will reveal functional roles of salivary proteins and how interplay between virus and insect modulates plant defense, insect physiology, inoculation competence, and behavior. The *F. occidentalis* detoxification and chemosensory genes also likely play a large role in the generalist lifestyle of this insect species. We found thrips-specific expansions within these gene families and this is consistent with the known role of these genes in perception and acceptance in diverse hosts and processing secondary metabolites. Notably, comparative transcriptomic studies of diverse plant-associated organisms revealed common themes in host-specialized transcriptomes and document the enrichment of genes that are secreted and may function as effectors, nutrient assimilation genes, and others involved in detoxification [148]. The rich and detailed information provided by this genome analysis opens broad, new avenues of basic and translational research for *F. occidentalis* and other thysanopteran species that will deeply impact the community of scientists and practitioners engaged in understanding this insect’s systematics, ecology, and role as a direct pest and as a vector of plant viruses.

## Methods

### Thrips rearing and genomic DNA isolation

A 10th generation sibling-sibling line of *F. occidentalis* (Pergande) was inbred for genome homozygosity from a lab colony originating from a progenitor isolated from the Kamilo Iki valley on the island of O’hau, Hawaii [281]. Thirty-one males and females were singly paired in small 1-oz clear plastic cups with lids fitted with thrips-proof-screen, and each cup contained a small cut segment of surface-disinfested green bean pod serving as the rearing and oviposition substrate. To reduce the likelihood of parthenogenetic reproduction in subsequent generations—as unfertilized female *F. occidentalis* produce only male progeny—early second instar larvae (L2) developing from each mating pair were removed with a fine, water-moistened paintbrush and transferred as single pairs to individual cups with a fresh cut bean to develop to adulthood. Pairs that did not develop into male-female pairs were discarded from their lineage. By the 10th generation, four inbred lines were moved to larger colony-size, 12-oz deli cups to initiate amplification of the lines, of which one thrived to establish a healthy, reproductive colony. Pools of adult females from this colony served as the biological material for genomic DNA isolation. Genomic DNA (gDNA) was isolated from eight, 10-mg subsamples of CO_2_-anesthetized females (hundreds of individuals per subsample) that were flash-frozen in liquid nitrogen, pulverized by hand with Kontes pestles (DWK Life Sciences, Thermo Fisher Scientific, Inc) then processed using the OmniPrep Genomic DNA Isolation Kit (G-Biosciences, Geno Technology, Inc., Saint Louis, MO, USA) following the manufacturer’s instructions, with the added step of 15 min room-temperature incubation of the dissolved pellet with 1 μl of LongLife RNAse (G-Biosciences) to remove residual RNA. The concentration of gDNA determined by a Nanodrop spectrophotometer (Thermo Fisher Scientific Inc.) ranged from 48 to 89 μg of DNA. Gel electrophoresis (2% agarose gel) resolved single bands of gDNA of greater than 24 kb in size. The gDNA samples were sent to the Baylor College of Medicine Human Genome Sequencing Center (BCM-HGSC) for sequencing.

### Genome sequencing and assembly of Focc_1.0

*Frankliniella occidentalis* was one of 28 arthropod species sequenced and assembled as a part of a pilot project for the i5K arthropod genomes project at the Baylor College of Medicine Human Genome Sequencing Center [280]. As with the other i5k species, an enhanced Illumina-ALLPATHS-LG sequencing and assembly strategy for *F. occidentalis* enabled multiple species to be approached in parallel at reduced costs. We sequenced four libraries of nominal insert sizes 180 bp, 500 bp, 3 kb, and 8 kb. The amount of sequence generated from each of these libraries is noted in Additional file 2: Table S1 with NCBI SRA accessions.

To prepare the 180-bp and 500-bp libraries, we used a gel-cut paired end library protocol. Briefly, 1 μg of the DNA was sheared using a Covaris S-2 system (Covaris, Inc. Woburn, MA) using the 180-bp or 500-bp program. Sheared DNA fragments were purified with Agencourt AMPure XP beads, end-repaired, dA-tailed, and ligated to Illumina universal adapters. After adapter ligation, DNA fragments were further size selected by agarose gel and PCR amplified for 6 to 8 cycles using Illumina P1 and Index primer pair and Phusion® High-Fidelity PCR Master Mix (New England Biolabs). The final library was purified using Agencourt AMPure XP beads and quality assessed by an Agilent Bioanalyzer 2100 (DNA 7500 kit) determining library quantity and fragment size distribution before sequencing.

The long mate pair libraries with 3-kb or 8-kb insert sizes were constructed according to the manufacturer’s protocol (Mate Pair Library v2 Sample Preparation Guide art # 15001464 Rev. A PILOT RELEASE). Briefly, 5 μg (for 2- and 3-kb gap size library) or 10 μg (8–10-kb gap size library) of genomic DNA was sheared to desired size fragments by Hydroshear (Digilab, Marlborough, MA), then end repaired and biotinylated. Fragment sizes between 3 and 3.7 kb (3 kb) or 8–10 kb (8 kb) were purified from 1% low melting agarose gel and then circularized by blunt-end ligation. These size selected circular DNA fragments were then sheared to 400-bp (Covaris S-2), purified using Dynabeads M-280 Streptavidin Magnetic Beads, end-repaired, dA-tailed, and ligated to Illumina PE sequencing adapters. DNA fragments with adapter molecules on both ends were amplified for 12 to 15 cycles with Illumina P1 and Index primers. Amplified DNA fragments were purified with Agencourt AMPure XP beads. Quantification and size distribution of the final library was determined before sequencing as described above.

Sequencing was performed on Illumina HiSeq2000s (Casava Version 1.8.3_V3) generating 100 bp paired end reads. Reads were assembled using ALLPATHS-LG (v35218) [282] on a large memory computer with 1 TB of RAM and further scaffolded and gap-filled using in-house tools Atlas-Link (v.1.0) [283] and Atlas gap-fill (v.2.2) [284]. The Focc_1.0 assembly was deposited in the NCBI GenBank (GCA_000697945.1) on 06/04/2014.

### Automated gene annotation using a Maker 2.0 pipeline tuned for arthropods

The 28 i5K pilot genome assemblies including *F. occidentalis* were subjected to automatic gene annotation using a Maker 2.0 annotation pipeline tuned specifically for arthropods. The pipeline is designed to be systematic: scalable to handle 100s of genome assemblies, evidence-guided using both protein and RNA-seq evidence to guide gen models, and targeted to utilize extant information on arthropod gene sets. The core of the pipeline was a Maker 2 [285] instance, modified slightly to enable efficient running on our computational resources. The genome assembly was first subjected to de novo repeat prediction and CEGMA analysis to generate gene models for initial training of the ab initio gene predictors. Three rounds of training of the Augustus [286] and SNAP [287] gene predictors within Maker were used to bootstrap to a high-quality training set. Input protein data included 1 million peptides from a non-redundant reduction (90% identity) of Uniprot Ecdysozoa (1.25 million peptides) supplemented with proteomes from eighteen additional species (*Strigamia maritime, Tetranychus urticae, Caenorhabditis elegans, Loa loa, Trichoplax adhaerens, Amphimedon queenslandica, Strongylocentrotus purpuratus, Nematostella vectensis, Branchiostoma floridae, Ciona intestinalis, Ciona savignyi, Homo sapiens, Mus musculus, Capitella teleta, Helobdella robusta, Crassostrea gigas, Lottia gigantean, Schistosoma mansoni*) leading to a final non-redundant peptide evidence set of 1.03 million peptides. RNA-seq transcription data derived from one sample each of adult males, females, and mixed larval and pupal stages (Additional file 2: Table S1) was used judiciously to identify exon-intron boundaries but with a heuristic script to identify and split erroneously joined gene models. We used CEGMA models for QC purposes: of 1977 CEGMA single-copy ortholog gene models, 1952 were found in the Focc_1 assembly and 1922 in the final predicted gene set—a reasonable result given the small contig sizes of the assembly. Finally, the pipeline used a nine-way homology prediction with human, *Drosophila* and *C. elegans*, and InterPro Scan5 to allocate gene names. The automated gene sets are available from the BCM-HGSC website [288] as well as the National Agriculture Library (NAL) i5k workspace sequence repository, data-share, and curation site for all i5k projects [67, 289] where a web-browser of the genome, annotations, and supporting annotation data is accessible.

### RNA evidence used to support manual genome curation

Both newly obtained and published *F. occidentalis* transcriptome resources were used to aid in manual annotation efforts. Using the cloud computing resources of CYVERSE (formerly iPlant Collaborative Discovery Environment) [290] and the Focc_1 genome assembly, the paired end Illumina HiSeq RNA-seq reads generated by BCM-HGSC for regular-lab-colony females, males, and pre-adults (pool of L1, L2, P1, and P2 stages) (this study, described above) were trimmed and cleaned with Prinseq-lite (version 0.20.4, [291]) and aligned and mapped to the genome with Tophat2-PE (v2.1.0). Two de novo assemblies (contigs) from published studies with *F. occidentalis*—one comprised of 454 sequencing reads for mixed stages of TSWV-infected and non-infected insects [23], and the other of Illumina RNA-seq reads for salivary glands of adult females and males [151] (NCBI TSA accession GAXD00000000.1), were mapped to the genome using GMAP locally [292]. These transcriptome resources were shared as RNA evidence tracks at the i5k Workspace@NAL [67]. In addition, Trinity de novo-assembled contigs [171] from Illumina HiSeq single-end reads (DNA Core Facility, University of Missouri, USA) were obtained from larval, pupal, and adult stages of *F. occidentalis* (+/− TSWV) (NCBI Bioproject PRJNA454326) to locate and connect fragmented cytochrome p450 (CYP) gene models using the BLAST tool at the i5k workspace.

### Phylogenomic analysis of the official gene set

The OrthoDB v8 resource [293] was queried to find shared orthologs among *F. occidentalis* and another eight arthropods genomes; *Daphnia pulex*, *Pediculus humanus*, *Acyrthosiphon pisum*, *Cimex lectularius*, *Apis mellifera*, *Tribolium castaneum*, *Danaus plexippus*, and *Drosophila melanogaster.* Custom Perl scripts (Additional file 10) were used to compute the number of genes in each category shown in Fig. 2a. For the phylogenomic analysis, only the single-copy orthologs were used to build a concatenated protein sequence alignment from which to estimate the phylogenetic tree using RAxML version 7.6.6 [294]. Briefly, a multiple sequence alignment was performed using muscle version 3.8.31 [295] for each orthologous group separately. Then, the resulting alignments were trimmed using trimAl version 1.2rev59 [70] with parameters “-w 3 -gt 0.95 -st 0.01”. The trimmed alignments were concatenated using the “seqret” program from the EMBOSS suite version 6.6.0.0 [296]. This concatenated alignment was used to build the phylogeny using RAxML version 7.6.6 with the PROTGAMMA model of amino acid substitutions and 100 bootstrap replicates.

### Assessment of gene set completeness and genome assembly quality

For evaluating the completeness of the *F. occidentalis* official gene set, and genome assembly, we used Benchmarking Universal Single-Copy Orthologs (BUSCO) [297] of the Arthropoda gene set, which consists of 1066 single-copy genes that are present in at least 90% of selected representative arthropods (shown in Fig. 2b). BUSCO assessments were run with the default parameters.

### Community curation of the thrips genome

Seventeen groups were recruited from the i5k pilot project and thrips research community to manually curate MAKER-predicted models (Focc_v0.5.3) of gene sets of interest to thrips consortium members using the Focc_1 assembly. The consortium used Web Apollo/JBrowser tools, online training, and written guidelines made available by the National Agriculture Library (NAL) i5k workspace, along with the RNA evidence described above, to locate and correct 1694 genes, 1738 mRNAs, and 13 pseudogenes. Gene set members were located with BLAST queries of hemipteroid and *Drosophila* orthologs using customized parameters provided in Additional file 1, sections entitled “Gene set manual annotations—customized strategy and phylogenetic analysis”. At the completion of the community curation period, the manually curated models were exported in gff3 format and quality-checked for formatting and curation errors, and then integrated with the MAKER-predicted gene models (Additional File 2: Table S2) to generate a non-redundant official gene set (OGS v1.0) (Additional File 2: Table S3). After removal of bacterial sequence contamination, the OGS v1.0 includes 16,859 genes, 16,902 mRNAs, and 13 pseudogenes.

### Phylogenetic analyses of gene families

To identify cases where there is clear evidence of multiple gene duplications in *F. occidentalis* leading to expanded repertoires of certain gene (sub)families of special interest, we (i) invested heavily in manual curation of *F. occidentalis* family members; (ii) retrieved high-quality previously curated sequences from other taxa, intentionally choosing exemplar species where prior curation provides confidence that comparisons would be fair, and therefore, inferences of gene family expansions would be expected to be robust; (iii) built phylogenetic trees for each family and/or subfamily; and (iv) compared the counts and branching/clustering of genes from each species in each tree. Particular algorithms and parameters used to generate the trees are provided in Additional file 1, sections entitled “Gene set manual annotations—customized strategy and phylogenetic analysis”, for each gene family examined phylogenetically.

### Improved assembly contiguity

Well after community curation of OGSv1.0 and subsequent analyses with targeted genes sets, a second assembly was generated in an attempt to improve contig and scaffold contiguity. This assembly (Focc_2) was generated using Platanus [298] followed by haplotype collapse with Redundans [299], two tools designed for short-read genome assembly of polymorphic short-read datasets, as described by Thomas et al. [280]. While the genome assembly had better contiguity statistics, the biological utility was judged not to be as good by the community, based on attempts to transfer and identify gene models in the new assembly. Because of this, the community reverted to the original version with manually annotated gene models as described in this paper, and this version (Focc_3.0) has been designated GCA_000697945.4 in NCBI. Focc_2.0 was deposited in NCBI as GCA_000697945.3 on 12/15/17.

### Identification of transcription factors

We identified likely transcription factors (TFs) by scanning the amino acid sequences of predicted protein-coding genes for putative DNA-binding domains (DBDs), and when possible, we predicted the DNA-binding specificity of each TF using the procedures described in Weirauch et al. [300]. Briefly, we scanned all protein sequences for putative DBDs using the 81 Pfam [301] models listed in Weirauch and Hughes [302] and the HMMER tool [303], with the recommended detection thresholds of Per-sequence Eval < 0.01 and Per-domain conditional Eval < 0.01. Each protein was classified into a family based on its DBDs and their order in the protein sequence (e.g., bZIPx1, AP2x2, Homeodomain+Pou). We then aligned the resulting DBD sequences within each family using clustalOmega [127] with default settings. For protein pairs with multiple DBDs, each DBD was aligned separately. From these alignments, we calculated the sequence identity of all DBD sequence pairs (i.e., the percent of AA residues that are exactly the same across all positions in the alignment). Using previously established sequence identify thresholds for each family [300], we mapped the predicted DNA-binding specificities by simple transfer. For example, the DBD of FOCC004897 (OGS ID) is 98% identical to the *Drosophila melanogaster* mirr protein (Additional file 2: Table S5). Since the DNA-binding specificity of mirr has already been experimentally determined, and the cut-off for Homeodomain family of TFs is 70%, we can infer that FOCC004897 will have the same binding specificity as mirr.

### Bacterial scaffold detection method

Bacterial scaffolds in *F. occidentalis* genome were identified using a modified nucleotide-based pipeline developed by Wheeler et al. [48] and as described previously [38]. Briefly, 1 kbp DNA fragments from each scaffold were searched for bacterial homologs against an in-house bacterial database containing 2100 bacterial species using BLASTn algorithm (Additional File 2: Table S25). The bacterial database was masked for low complexity regions by NCBI Dustmasker [304], and similarity matches of bitscore above 50 were retained. To accurately determine the bacterial scaffolds, parameters including the number of bacterial matches per scaffold, proportion of the scaffold covered by bacterial matches, and total hit width (encompassing the distance between the leftmost and rightmost bacterial match in the scaffold) were considered. Candidate bacterial scaffolds were called based on a cut-off of ≥ 0.40 proportion bacterial hit width as this criterion with manual curation of the sequences. It should be noted, however, that represented in this set could be larger lateral gene transfers in relatively small scaffolds, as the latter cannot be readily identified without flanking eukaryotic sequences and/or further manual curation. The procedure for detecting bacterial scaffolds was performed twice with modifications—once during the early stages of the curation process (2015) using an “older” method [48] and a second time more recently as described above using this “new” method after the curation process ended and OGS v1.0 was frozen and submitted for publication.

### Lateral gene transfer detection method

Candidate bacterial lateral gene transfers were identified using the method above for detecting bacterial scaffolds [38, 48, 304]. Analysis was limited to scaffolds more than 100 kb due to the need for flanking sequences to properly evaluate candidate LGTs. We examined each bacterial match with a bitscore > 75 that also showed a bitscore = 0 in a reference eukaryote database. Fragments flanking the 1-kbp positive hits were examined and combined for LGT analysis.

Manual curation was conducted on candidates surviving the initial filtering steps. Each candidate was searched with BLASTn to the NCBI nr/nt database. Sequences homologous to insect genes with the exception of a possible LGT in the common ancestor of closely related insects were removed. In cases where the matches to other insects were sporadic, the candidate was retained, as our experience has indicated that these can be independent LGTs into different lineages. The region was additionally searched with BLASTx to the NCBI nr/nt database. Sequences with no hits to multiple insect proteins were identified as an LGT candidate. The best bacterial match to the candidate was called using the NCBI nr and protein databases, and flanking genes within the scaffold were examined to determine whether they are eukaryotic or bacterial. We examined whether the LGT region was associated with an annotated gene within the insect genome, and if RNA sequencing data showed evidence of transcriptional activity in the LGT region. In a few cases, polymerase chain reactions were also conducted using primers that bridge the LGT candidate and flanking eukaryotic-like sequences (Additional file 1: Section 2, Table S2.2).

### PCR of LGT candidate flanking regions

The overlapping primers used to verify whether the potential LGTs were present in the thrips genome were designed using DNAMAN 7.0 (Lynnon Biosoft, Quebec, Canada). The PCR was carried out in a 20-μL reaction volume containing 4 μL 5x Phusion HF Buffer or 5x Phusion GC Buffer (depending on the difficulty in amplifying the target fragment), 0.4 μL 10 mM dNTPs, 1.0 μL 10 mM each primer, 0.6 μL DMSO, 0.2 μL Phusion DNA High-Fidelity DNA Polymerase (Thermo Scientific), 1.0 μL genome DNA template, and 11.8 μL ddH_2_O. The cycling program was set at 98 °C for 30 s, and then 34 cycles of 98 °C for 10 s, and 72 °C for ~ 2 min (30 s/kb), followed by a final extension step of 72 °C for 10 min and 4 °C hold. The PCR products were checked on a 1.0% gel after electrophoresis and then purified using a Wizard SV Gel and PCR Clean-Up System (Promega). The purified fragments were cloned into pGEMT Easy vector (Thermo Scientific) and transformed into DH5α competent cells. The positive transformants were cultured for plasmid purification using the E.Z.N.A. Plasmid Mini Kit I, (V-spin) (Omega Bio-tek) and then sent for sequencing by LGC genomics (https://www.lgcgroup.com). Overlapping sequences obtained from the different amplification reactions were then re-assembled and used to verify if the LGT was indeed present in the thrips genome.

### Phylogenetic analysis and nucleotide sequence evolution of putative LGTs

The most promising LGT candidates were further analyzed by evaluating phylogenetic relationships and conducting branch specific synonymous and non-synonymous rate analysis with homologous references from NCBI to detect signatures of stabilizing or directional selection.

For phylogenetic analysis of protein sequences, the top proteins with the strongest bitscores (up to 50–60 proteins) to the translated region of the *F. occidentalis* proteins were aligned using muscle in MEGA. Protein alignments were assessed for misalignment and large in-del regions were removed. Phylogenetic trees were constructed by RAXML protein model “LG” with 1500 bootstrap replications. Outgroups were identified by identifying the bacterial species closest to the LGT in the constructed phylogeny and taking the bacteria protein and blasting to NCBI’s nr protein database restricted to the bacteria’s taxonomic group (e.g., family or order). Another phylogenetic tree including proteins within different genera from the bacteria taxonomic family was constructed.

To characterize nucleotide sequence evolution of the LGT and related sequences (e.g., synonymous and non-synonymous substitution rates), the translated nucleotide sequence were identified using the protein query from representative species throughout the protein tree and by comparing the similarity to the NCBI nucleotide database restricted to the specific species. The sequences obtained were then aligned using MUSCLE and large in-del regions were removed. A pruned version of the protein tree was created with the same representative sequences obtained for the nucleotide alignment. HYPHY’s BUSTED and BUSTEC (Branch-site Unrestricted Statistical Test of Episodic Diversification/Conservation), as described in [56], were both performed to validate the open reading frame using the nucleotide aligned sequences and the pruned protein tree by testing for positive and purifying selection respectively. The branch specific non-synonymous (dN) and synonymous (dS) were calculated using PAML CODEML free-model rate with fixed-branch lengths corresponding to the condensed protein tree and using the representative nucleotide alignment. Protein trees in Newick format used for the PAML, BUSTEC, and BUSTED analysis with NCBI accession numbers (Example XP_026279074.1: *Frankliniella occidentalis*) and corresponding branch lengths are provided in Additional file 1: Section 2.

### Use of expression data and published transcriptomes to infer provisional functionality of genes involved in selected processes of interest

#### Identification of salivary gland (SG)-associated genes and enriched expression

Using methods developed by Telleria et al. [305] and Ribeiro et al. [306], with modifications, a comparative analysis of transcript-level expression in SG tissues to whole bodies was performed on existing RNA-seq datasets to unambiguously identify SG-associated genes in the *F. occidentalis* genome and to identify genes/contigs exhibiting SG-enriched expression. Because feeding behaviors, tissue damage caused by feeding, and virus inoculation efficiency appear to be sexually dimorphic traits in *F. occidentalis*, we capitalized on male and female RNA-seq data sets that were generated to assist in gene prediction for this genome project (Additional file 2: Table S1, NCBI SRA Accession = SRX897632 and SRX897634), and from salivary gland tissues (principal and tubular combined) of males and females (SRS549985, SRS549981, SRS549977, SRS549984, SRS549980, SRS54997) as previously published [151].

RNA-seq datasets were individually mapped to predicted genes from the *F. occidentalis* genome project using CLC Genomics Workbench 11.0 based upon settings previously described [305] with the exception that transcripts per million (TPM) was used as a proxy for gene expression. Fold changes were determined as the TPM for the salivary gland RNA-seq sets divided by the TPM for whole-body datasets. Baggerly’s test (*t*-type test statistic) [307] followed by a false discovery rate (FDR) at 0.05 [308] was used to identify genes with significant enrichment in the salivary glands. Enriched genes were removed, and mapping and expression analyses were repeated to ensure low expressed genes were not missed. In addition, the RNA-seq data sets were mapped to the transcriptome previously generated from the salivary gland RNA-seq datasets [151], enriched contigs were identified as before, and a second mapping and analysis following removal of initially enriched contigs was utilized to identify low expressed salivary gland-enriched contigs. This secondary analysis was conducted to identify transcripts that were not predicted in the genome or may have not been present on an assembled scaffold.

Contigs and genes were compared to reduce overlap to a combined final SG-enriched sequence set that was generated. Briefly, blastn comparison was utilized to match sequences and only the longest sequence was retained if 100% matched was noted. After merging the non-overlapping predicted genes and contigs, the SG-enriched sequence was searched (BLASTx) against multiple NCBI non-redundant proteins databases including those for arthropods, hemipterans, viral, bacterial, plant, *Drosophila*, and the complete nr set with an expectation value (E-value) of at least 0.001. For transcripts with a blast hit with an E-value above 0.001, the identification was based upon the best match that included previously assigned biological function (lipase, cellulase, etc.). Following this process, transcripts with enriched expression in the salivary glands of male, females, and combined (males and females) relative to the entire body were compared to determine those that overlap between each set. Publicly available bioinformatics tools were used to make in silico predictions of structural features in the SG peptide sequences—SignalP (v.5.0) software [309] to determine the presence of a eukaryotic signal peptide cleavage site on the N terminus of the protein; TMHMM (v.2.0) software [310] to identify transmembrane domains of 18 amino acids or greater; and DeepLoc (v.1.0) [311] to predict cellular localization of the protein. SG proteins that were determined to contain a canonical signal peptide, to have an extracellular or cytoplasmic localization pattern, and/or a transmembrane domain associated with outer membranes were considered putative secretory proteins.

Real-time quantitative reverse-transcription PCR (qRT-PCR) was performed on a subset of putative SG gene transcripts to validate the comparative RNA-seq approach (Additional file 1: Section 5). *Frankliniella occidentalis* females were obtained from a colony originally collected from the same Hawaiian isolate used in this study, and maintained on green bean pods [4]. Females were collected 48 h post-eclosion and salivary glands were surgically removed as previously described [151] to achieve three sample groups: salivary glands (PSG and TSG combined), head (minus SG), and body (i.e., carcass). Each sample group consisted of five females to ensure adequate quantities of total RNA, and six biological replications of dissection and total RNA was isolated for each sample group. Dissections were conducted at the same time of day within a 2-h window to minimize experimental variation (error) between sample groups.

Total RNA was extracted from thrips tissues using the Arcturus® PicoPure® RNA isolation kit (Life Technologies, USA), yields were determined by Nanodrop ND-1000 (Thermo Scientific, DE, USA), and 2 ng of total RNA templates was used to synthesis first-strand cDNA with 0.5 M of gene-specific reverse primers of the target gene and reference gene (see Additional file 1: Table S5.1) with the Verso™ cDNA synthesis Kit (Thermo Scientific, DE, USA), including the RT enhancer to remove residual DNA, in 10 μL reaction volumes for 60 min incubation at 45 °C, followed by a 10-min denaturation step at 85 °C. No-RT controls were also included in the cDNA step to ensure that possible contribution of DNA contamination to real-time PCR values (Cq) was negligible. Real-time PCR reaction mixtures (10 μl volumes) were prepared with 2 μL of cDNA, 0.2 μM of forward and reverse gene-specific primers, and SsoAdvanced™ Universal SYBR® Green Supermix (Bio-Rad Laboratories, CA), and run on a CFX96™ real-time PCR detection system (Bio-Rad Laboratories, CA) with an amplification protocol as follows: 95 °C for 30 s, 40 cycles of 95 °C for 10 s, 55 °C for 10 s, and 60 °C for 25 s, followed by a melting protocol to evaluate product quality (absence of primer-dimer and single peaks). Each plate was run with one biological replicate of each sample group (SGs, heads, bodies), four primer pairs (two putative SG genes and two reference genes) in separate wells, and three technical replicates of each PCR reaction. The two reference genes selected were *F. occidentalis* actin (Act, [154], and cytochrome oxidase 1 (COX [155]). Normalized expression of each of the four SG-enriched transcripts was calculated using the 2^−ΔCq^ method [156] and the geometric mean of Act/COX reference Cqs. Normalized expression was log transformed prior to one-way ANOVA followed by Tukey’s multiple comparison tests with GraphPad Prism software.

#### Comparative analysis of innate immune gene transcripts in three thrips vector species

One key finding of the *F. occidentalis* genome annotation was the apparent absence of an IMD gene—a core signal transducing protein in one of the evolutionarily conserved pathways involved in the production of antimicrobial peptides in insects—and the downstream FADD gene. With the availability of transcriptome sequences of three thrips vector species, we performed comparative analyses to mine for IMD pathway members and other core innate immunity-associated genes. A genome-enabled, transcriptome assembly representing TSWV-infected and non-infected adult *F. occidentalis* (PRJNA454326, [24]) and two de novo-assembled transcriptomes, one representing mixed stages of TSWV-infected and non-infected *F. fusca* (PRJNA385691, [25] and the other representing CaCV-infected and non-infected adult *Thrips palmi* (PRJNA498538, [26] were annotated against a custom made database of arthropod innate immunity genes downloaded from ImmunoDB [312] using the blastx algorithm in local BLAST+ v. 2.8.1 with an E-value cut-off of 10^− 5^. Blast annotations were filtered to retain annotations with highest bit score, lowest E-value, and longest alignment length.

Transcripts encoding immune-related genes in *F. occidentalis*, *F. fusca*, and *T. palmi* were translated in six frames using TransDecoder (Version5.5.0, [221]) with a minimum peptide length of 100 amino acids. Translated transcripts were annotated against the UniProt database downloaded on April 9, 2019, using Blastp with an E-value cut-off of 10^− 5^. Redundant translated proteins were removed by k-mer analysis to develop an initial training set for constructing Markov Model to discriminate between coding and non-coding regions. Predicted proteins homologous to proteins in the UniProt database were retained for further analysis. Orthologous innate immunity genes between these three species were identified by Orthovenn2, a web-tool used to identify orthologous and paralogous genes, with a pairwise sequence similarity cut-off of 10^− 5^ and an inflation of 1.5 to define orthologous cluster structure [222]. Orthologous clusters were analyzed by UniProt search and GO Slim for functional annotation.

#### Gene expression patterns associated with postembryonic development

To aid in annotation of molting and metamorphosis genes, notably those with multiple copies and putative duplicates in the genome, and to globally characterize gene expression associated with progression from *F. occidentalis* larvae to adulthood, we performed weighted correlation network analysis (WGCNA) on normalized read counts (FPKM values) generated for healthy first-instar larvae (L1), propupae (P1), and adults (mixed males and females) across three biological replicates per developmental stage from a previously published RNA-seq study [24] (NCBI Bioproject PRJNA454326, SRA accessions: SRX4015378, SRX4061765, SRX4061766, SRX4159448, SRX4159449, SRX4159450, SRX4159451, SRX4159452). WGCNA is a clustering method that places highly correlated transcripts into colored modules based on similar patterns of expression across samples [43]. The resulting modules are used to form a correlation network to identify correlated genes in transcriptome-wide expression data sets (e.g., RNA-seq count data) and to identify the strength of associations (statistically significant correlations) between these groups of co-expressed genes (modules) and external sample traits of interest (developmental stage in the present study). Genes of zero variance were filtered out from the RNA-seq data in preparation for WGCNA. A scale-free topology threshold of 0.8 was used to identify the proper soft power of 16 for analysis. Adjacency matrix was calculated for signed network construction. Modules were determined by the dynamic tree cutting algorithm with a minimum of 20 genes per module. After relating modules to external sample traits, modules with the highest correlations were selected for further analysis using REVIGO [255] on stage-associated genes and gene ontologies (GO terms) to filter out functional redundancies between gene ontology (GO) terms (e.g., genes enriched in child terms that are counted in their associated parent terms) prior to visualization of semantically similar GO clusters for each developmental stage. Using a targeted approach, we repeated the WGCNA analysis on the curated molting and metamorphosis gene set using the same parameters, validating 60–85% clustering of these genes into modules discriminated by the global network analysis.

#### Identification of sex-specific genes

To generate a repertoire of sex-specific genes and to identify genes associated with *F. occidentalis* reproduction, we performed differential gene expression analyses on RNA-seq data for male, female, and pre-adult (larvae and pupae mixed) samples that were generated to assist in gene prediction for this genome project (Additional file 1: Table S1, NCBI Bioproject: PRJNA203209, SRA accessions: SRX897632, SRX897634, SRX897633) using a previously described strategy [313, 314]. Additional thrips species sequenced as a part of the 1KITE project (*Frankliniella cephalica*, PRJNA219559; *Gynaikothrips ficorum*, PRJNA219563; *Thrips palmi*; PRJNA219609) were used for identification of conserved sex- and stage-specific genes between these thrips species.

The *F. occidentalis* RNA-seq set was assessed with FastQC and trimmed with CLC Genomics. Reads were permitted to match up to five locations with only two mismatches and required at least 90% similarity at 70% of transcript length. Transcript levels were normalized to transcripts per million (TPM) and significant enrichment was determined with Baggerly’s test (beta-binomial distribution statistic) followed by Bonferroni correction (at 0.01) and a two-fold difference between samples. Gene identification was obtained through BLASTx searches against an NCBI non-redundant protein arthropod database with an E-value cut-off of 0.001. RNA-seq sets for the other thrips species were BLAST-searched against the finalized female, male, and pre-adult *F. occidentalis* sets (E-value ≤ 1.00 × 10^− 20^). Venn diagrams [315] (http://bioinformatics.psb.ugent.be/webtools/Venn/) were used to depict the number of shared genes in female, male, and pre-adult thrips. Fold change and the number of unique gene reads from the conserved female and male thrips genes were used to identify highly enriched genes (> 1000 unique reads, > 4-fold difference) in female and male thrips.

## Supplementary information

**Additional file 1.** Supplementary notes, figures and small tables.

**Additional file 2.** Workbook of large supplementary tables.

**Additional file 3.** Weighted correlation network analysis (WGCNA) modules of co-expressed gene sets of developmental stages.

**Additional file 4.** Chemosensory receptor sequences.

**Additional file 5.** Neuropeptide and protein hormone peptide sequences with highlighted features.

**Additional file 6.** Complete and partial cytochrome p450 (CYP) sequences.

**Additional file 7.** Complete ATP-binding cassette transporter (ABC) and carboxylesterase sequences.

**Additional file 8.** Supporting tables for Orthovenn comparison of innate immunity-associated transcripts in thrips vector species.

**Additional file 9.** Supporting tables of RNAseq expression outputs for conserved sex-specific genes in thrips species.

**Additional file 10.** Custom Perl scripts used to compute the number of genes in each category of the phylogenomic analysis of the official gene set shown in Fig. 2a.

## Data Availability

The datasets supporting the conclusions of this article are publicly available at the NCBI, Bioproject PRJNA203209, and in the United States Department of Agriculture’s National Agricultural Library (NAL), AgData Commons repository [316–318]. Also, scaffolds, gene models, and genome browser are available at the NAL i5k workspace (https://i5k.nal.usda.gov/Frankliniella_occidentalis). Previously published transcriptome data used for particular analyses are publicly available at the NCBI, Bioprojects: PRJNA454326 [subset: SRX4015378, SRX4061765, SRX4061766, SRX4159448, SRX4159449, SRX4159450, SRX4159451, SRX4159452]; PRJNA219559; PRJNA219563; PRJNA219609; PRJNA234733 [subset: SRS549985, SRS549981, SRS549977, SRS549984, SRS549980, SRS54997]; PRJNA385691 [subset: SRS2169626, SRS2169627, SRS2169625]; and PRJNA498538 [subset: SRS3983472, SRS3983473, SRS3983474].
