# Correction to: Genome-enabled insights into the biology of thrips as crop pests

**DOI:** 10.1186/s12915-020-00915-z

**Published:** 2020-11-16

**Authors:** Dorith Rotenberg, Aaron A. Baumann, Sulley Ben-Mahmoud, Olivier Christiaens, Wannes Dermauw, Panagiotis Ioannidis, Chris G. C. Jacobs, Iris M. Vargas Jentzsch, Jonathan E. Oliver, Monica F. Poelchau, Swapna Priya Rajarapu, Derek J. Schneweis, Simon Snoeck, Clauvis N. T. Taning, Dong Wei, Shirani M. K. Widana Gamage, Daniel S. T. Hughes, Shwetha C. Murali, Samuel T. Bailey, Nicolas E. Bejerman, Christopher J. Holmes, Emily C. Jennings, Andrew J. Rosendale, Andrew Rosselot, Kaylee Hervey, Brandi A. Schneweis, Sammy Cheng, Christopher Childers, Felipe A. Simão, Ralf G. Dietzgen, Hsu Chao, Huyen Dinh, Harsha Vardhan Doddapaneni, Shannon Dugan, Yi Han, Sandra L. Lee, Donna M. Muzny, Jiaxin Qu, Kim C. Worley, Joshua B. Benoit, Markus Friedrich, Jeffery W. Jones, Kristen A. Panfilio, Yoonseong Park, Hugh M. Robertson, Guy Smagghe, Diane E. Ullman, Maurijn van der Zee, Thomas Van Leeuwen, Jan A. Veenstra, Robert M. Waterhouse, Matthew T. Weirauch, John H. Werren, Anna E. Whitfield, Evgeny M. Zdobnov, Richard A. Gibbs, Stephen Richards

**Affiliations:** 1 Department of Entomology and Plant Pathology, North Carolina State University, Raleigh, NC 27695 USA; 2 Virology Section, College of Veterinary Medicine, University of Tennessee, A239 VTH, 2407 River Drive, Knoxville, TN 37996 USA; 3 Department of Entomology and Nematology, University of California Davis, Davis, CA 95616 USA; 4 Laboratory of Agrozoology, Department of Plants and Crops, Ghent University, Coupure Links 653, 9000 Ghent, Belgium; 5 Institute of Molecular Biology and Biotechnology, Foundation for Research and Technology-Hellas, Vassilika Vouton, 70013 Heraklion, Greece; 6 Department of Genetic Medicine and Development, University of Geneva Medical School, and Swiss Institute of Bioinformatics, Geneva, Switzerland; 7 Institute of Biology, Leiden University, 2333 BE Leiden, The Netherlands; 8 Institute for Zoology: Developmental Biology, University of Cologne, 50674 Cologne, Germany; 9 Department of Plant Pathology, University of Georgia - Tifton Campus, Tifton, GA 31793-5737 USA; 10 National Agricultural Library, USDA-ARS, Beltsville, MD 20705 USA; 11 Department of Plant Pathology, Kansas State University, Manhattan, KS 66506 USA; 12 Department of Biology, University of Washington, Seattle, WA 98105 USA; 13 Chongqing Key Laboratory of Entomology and Pest Control Engineering, College of Plant Protection, Southwest University, Chongqing, China; 14 International Joint Laboratory of China-Belgium on Sustainable Crop Pest Control, Academy of Agricultural Sciences, Southwest University, Chongqing, China and Ghent University, Ghent, Belgium; 15 Department of Botany, University of Ruhuna, Matara, Sri Lanka; 16 Human Genome Sequencing Center, Department of Human and Molecular Genetics, Baylor College of Medicine, One Baylor Plaza, Houston, TX 77030 USA; 17 Department of Biological Sciences, University of Cincinnati, Cincinnati, OH 45221 USA; 18 IPAVE-CIAP-INTA, 5020 Cordoba, Argentina; 19 Department of Biology, Mount St. Joseph University, Cincinnati, OH 45233 USA; 20 Department of Biology, University of Rochester, Rochester, NY 14627 USA; 21 Queensland Alliance for Agriculture and Food Innovation, The University of Queensland, QLD, St. Lucia, 4072 Australia; 22 Department of Biological Sciences, Wayne State University, Detroit, MI 48202 USA; 23 School of Life Sciences, University of Warwick, Gibbet Hill Campus, Coventry, CV4 7AL UK; 24 Department of Entomology, Kansas State University, Manhattan, KS 66506 USA; 25 Department of Entomology, University of Illinois at Urbana-Champaign, Urbana, IL 61801 USA; 26 INCIA UMR 5287 CNRS, University of Bordeaux, Pessac, France; 27 Department of Ecology and Evolution, Swiss Institute of Bioinformatics, University of Lausanne, 1015 Lausanne, Switzerland; 28 Center for Autoimmune Genomics and Etiology, Divisions of Biomedical Informatics and Developmental Biology, Cincinnati Children’s Hospital Medical Center, Cincinnati, OH 45229 USA; 29 Department of Pediatrics, University of Cincinnati, College of Medicine, Cincinnati, OH 45229 USA

**Correction to: BMC Biology 18, 142 (2020)**

**https://doi.org/10.1186/s12915-020-00862-9**

Following publication of the original article [1], the authors would like to remove the phrase ‘vertically transmitted’ from the last sentence in the fourth paragraph under the heading **Background**.

The sentence originally read:

In addition to serving as crop disease vectors, thrips support vertically transmitted, facultative bacterial symbionts that reside in the hindgut [27, 28].

The sentence should read:

In addition to serving as crop disease vectors, thrips support facultative bacterial symbionts that reside in the hindgut [27, 28].

The original article [1] has been corrected.
