# Supplementary material for: Genome-enabled insights into the biology of thrips as crop pests
Source: BMC Biol. 2020 Oct 19;18:142. doi: 10.1186/s12915-020-00862-9 (PMC7570057; doi:10.1186/s12915-020-00862-9)
Supplement: Supplementary file 1 — Additional file 1. Supplementary notes, figures and small tables. [file 12915_2020_862_MOESM1_ESM.docx]

*Frankliniella occidentalis* (FOCC) genome paper

Additional file 1: Supplementary methods, annotation notes, small tables and phylogenetic trees contributed by the FOCC genome annotation consortium.

The contents of this supplement provide supporting details that were too extensive to include in the primary paper. Analysis of gene sets and expression data are provided by section (see table of contents), and supplemental tables and figures are numbered sequentially by section number and referred to as such in the primary paper. The type, amount and style of information presented in each section may differ due to the priorities set by the different members of the FOCC genome annotation consortium.

Note: The gene model identifiers (FOCC) in this supplement are the original MAKER gene model identifiers PRIOR to consortium curation of the selected gene sets and reassignment of OGS v1.0 identifiers. The OGS v1.0 identifiers that correspond to the MAKER identifiers in this supplement can be found in Additional file 2: Table S2.

**Table of Contents**

[1. Homeodomain transcription factor gene clusters (Hox, Iro-C) and Synteny 4](#_Toc46083386)

[1.1. Abstract 4](#_Toc46083387)

[1.2. Cluster reconstruction 4](#_Toc46083388)

[1.3. Gene structure and protein coding sequence divergence in thrips 4](#_Toc46083389)

[2. Lateral Gene Transfers 6](#_Toc46083390)

[2.1. Abstract 6](#_Toc46083391)

[2.2. Potential Bacterial Scaffold Detection 7](#_Toc46083392)

[2.3. Lateral gene transfers in *F. occidentalis* 7](#_Toc46083393)

[2.3.1. O-methyltransferase 7](#_Toc46083394)

[2.3.2. Mannanase 7](#_Toc46083395)

[2.3.3. Levanase 8](#_Toc46083396)

[3. Chemosensory receptors 15](#_Toc46083397)

[3.1. Gene set manual annotations – customized strategy and phylogenetic analysis 15](#_Toc46083398)

[3.2. Gene modeling difficulties 15](#_Toc46083399)

[3.3. The OR family 17](#_Toc46083400)

[3.4. The GR family 18](#_Toc46083401)

[3.5. The IR family 20](#_Toc46083402)

[4. Vision genes 23](#_Toc46083403)

[4.1 Background 23](#_Toc46083404)

[4.2 Gene set manual annotations – customized strategy and phylogenetic analysis 23](#_Toc46083405)

[4.3 Vision genes 23](#_Toc46083406)

[5. Validation of salivary gland-enriched transcripts 25](#_Toc46083407)

[5.1. Abstract 25](#_Toc46083408)

[5.2. Results and Discussion 25](#_Toc46083409)

[6. Detoxification genes 29](#_Toc46083410)

[6.1. Cytochrome P450s 29](#_Toc46083411)

[6.1.1. Abstract 29](#_Toc46083412)

[6.1.2. Gene set manual annotations – customized strategy and phylogenetic analysis 29](#_Toc46083413)

[6.1.3. Results and Discussion 30](#_Toc46083414)

[6.2 ATP binding cassette and Carboxylesterase genes 34](#_Toc46083415)

[6.2.1 Gene set manual annotations – customized strategy and phylogenetic analysis 34](#_Toc46083416)

[6.2.2. Results and Discussion 35](#_Toc46083417)

[7. Innate Immune genes 45](#_Toc46083418)

[**7.1. Abstract** 45](#_Toc46083419)

[**7.2. Background** 45](#_Toc46083420)

[**7.3. Innate immunity-associated genes** 46](#_Toc46083421)

[**7.4. Comparison of innate immunity transcripts of *F. occidentalis*, *F. fusca* and *T. palmi*** 46](#_Toc46083422)

[7.5 RNAi pathway genes 49](#_Toc46083423)

[8. Embryonic and Post-embryonic genes 56](#_Toc46083424)

[8.1 *Wnt* Signaling Pathway 56](#_Toc46083425)

[8.1.1. Abstract 56](#_Toc46083426)

[8.1.2. Gene set manual annotations – customized strategy 56](#_Toc46083427)

[8.1.3. Results and Discussion 57](#_Toc46083428)

[8.2 Molting and Metamorphosis 61](#_Toc46083429)

[8.2.1. Juvenile hormone esterase (JHE) 61](#_Toc46083430)

[8.2.2. bHLH PAS and bHLH Myc family member proteins 61](#_Toc46083431)

[8.2.3. bHLH super family protein 62](#_Toc46083432)

[9. Cuticular Proteins 65](#_Toc46083433)

[9.1. Results 65](#_Toc46083434)

[10. REFERENCES CITED 68](#_Toc46083435)

# 1. **Homeodomain transcription factor gene clusters (Hox, Iro-C) and Synteny**

*Contributed by Kristen A. Panfilio and Iris M. Vargas Jentzsch*

## 1.1. Abstract

The Hox and Iroquois Complex (Iro-C) gene clusters encode highly conserved homeodomain transcription factors with essential roles in development. The Hox cluster is conserved across the Bilateria (Krumlauf, 1992), and the Iro-C is found throughout the Insecta (Cavodeassi et al., 2001, McNeill et al., 1997). Annotation of the genes in these clusters provides an indicator of draft genome quality and an opportunity to assess synteny among species. In *Frankliniella occidentalis* we could construct single copy gene models for all expected orthologs. In terms of synteny, we could reconstitute the small Iro-C and there is partial assembly of the larger Hox cluster.

### 1.2. Cluster reconstruction

We were able to find and annotate gene models for all ten Hox cluster genes, split across four different scaffolds (**Figure S1.1A**). All linked Hox genes occurred in the expected order and with the expected, shared transcriptional orientation. While these findings would suggest that the current draft assembly is correct but simply incomplete, a note of caution arises from an assessment of estimated cluster size. Assuming direct concatenation of these four scaffolds, the Hox cluster would span a region of 5.9 Mb in a genome with a total size of 415 Mb, which is disproportionately large (3.5-fold larger relative cluster size compared to previously analyzed i5k pilot species and the beetle *Tribolium castaneum*) and suggests incorrect assembly of the non-Hox portions of some of these scaffolds. For example, the scaffold regions upstream of *Hox1/labial* and of *Hox4/Deformed* are surprisingly large. (Note that while the Hox genes are numbered in ascending order in the 5' to 3' direction, the genes are in fact transcribed on the opposite strand. Hence, “upstream” of *Hox1* refers to the genomic region between *Hox1* and *Hox2*.)

Assembly limitations are also manifest in that only partial gene models could be created for *Ultrabithorax* and *Abdominal-B*, where the missing coding sequence includes the highly conserved homeobox, which encodes the key functional domain of the DNA-binding homeodomain (**Figure S1.1B**).

For the small Iroquois Complex, clear, single copy orthologues of both *iroquois* (*iro*) and *mirror* (*mirr*) are indeed linked in the current assembly (**Figure S.1.1C**). As expected by conservation, the genes occur in the same transcriptional orientation, with *iro* upstream of *mirr*, and with no intervening non-Iro-C genes. Also, unlike the Hox cluster estimation, the *Frankliniella* Iro-C is conserved for the ratio of cluster size to genome size, despite gaps on the relevant scaffold.

### 1.3. Gene structure and protein coding sequence divergence in thrips

Although all ten *Frankliniella* Hox genes could be identified and their orthology is clear, they are in some features rather divergent compared to other insects (*Zootermopsis nevadensis, Cimex lectularius, Oncopeltus fasciatus, Pediculus humanus corporis, Tribolium castaneum, Anoplophora glabripennis*, and *Drosophila melanogaster*). Specifically, *Focc-zerknüllt* encodes the largest protein among these orthologs (439 aa compared to a mean of 300 aa), while *Focc-Antennapedia* and *Focc-abdominal-A* encode larger proteins than all other species except for *Drosophila* (~14% larger than the mean, excluding *Drosophila*). Meanwhile, three of the *Frankliniella* Hox genes – *Deformed, fushi tarazu,* and *abdominal-A* – have uniquely acquired additional introns that interrupt coding sequence exons in what are otherwise highly conserved gene structures across the Insecta (>300 myr divergence time). While the gene locus is incomplete, the partial model for *Abdominal-B* has also acquired additional introns, which interrupt exons that encode the 5' UTR.


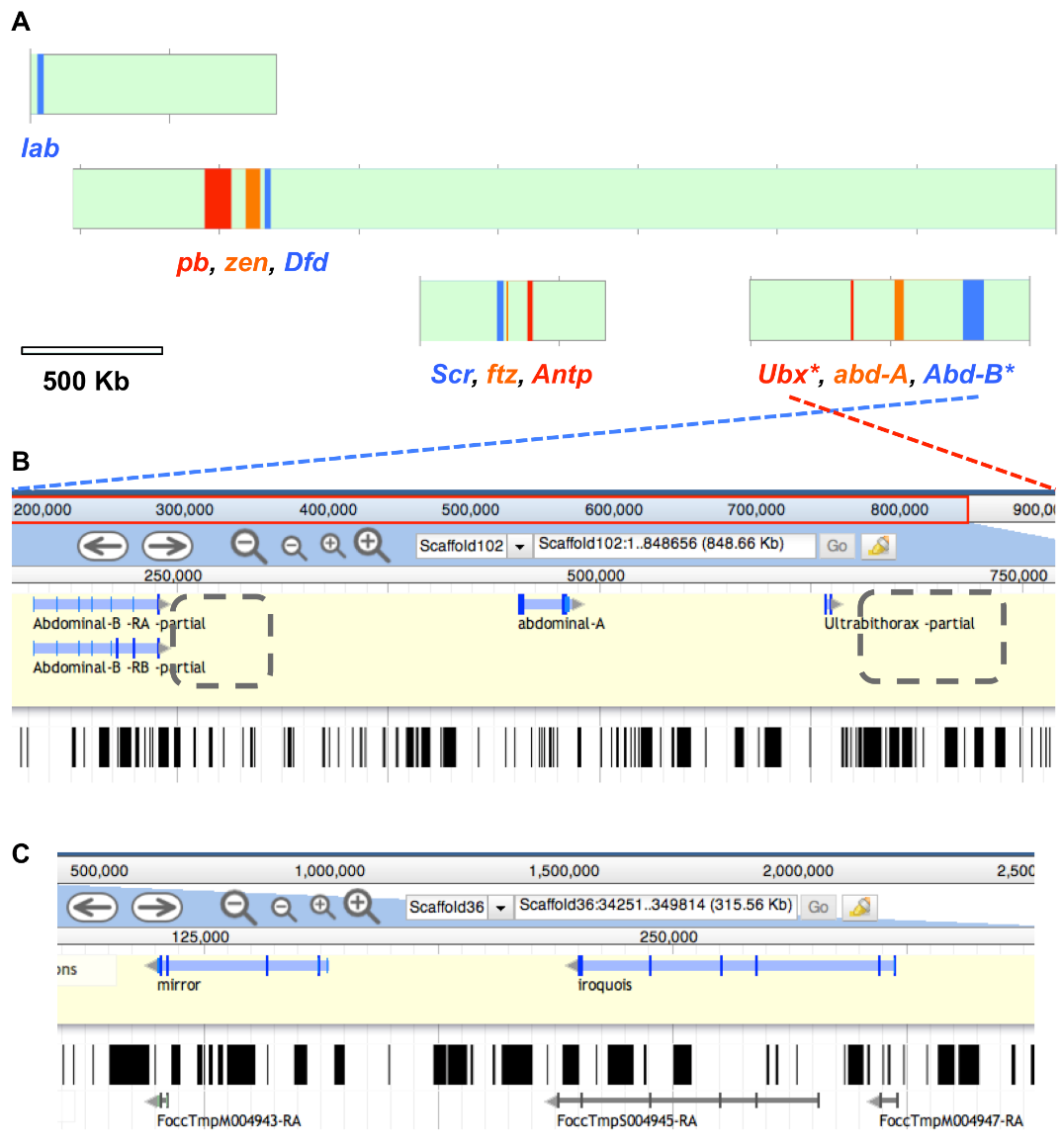


**Figure S1.1. *Frankliniella* Hox and Iro-C clusters.** (A) Scaffolds containing Hox genes are shown to scale (green), with Hox gene loci indicated. Asterisks indicate incomplete gene models. (B) Genome browser screenshot for the posterior Hox genes. Dashed gray boxes indicate where missing coding sequence exons would be expected, and gaps in the assembly are indicated below by black bars. (C) Genome browser screenshot of the Iro-C.

**Table S1.1.** Positional information for the annotated homeobox genes. Incomplete gene models are marked with an asterisk (*).

| **Gene** | **Scaffold: start..end** | **Locus length (nt)** | **Protein length (aa)** | **Number of CDS exons** |
| --- | --- | --- | --- | --- |
| *labial* | Scaffold124:24063-47398 | 23,336 | 368 | 2 |
| *proboscipedia* | Scaffold11:2956730-3052532 | 95,803 | 733 | 4 |
| *zerknüllt* | Scaffold11:2852821-2905256 | 52,436 | 439 | 2 |
| *Deformed* | Scaffold11:2814925-2836826 | 21,902 | 447 | 3 |
| *Sex combs reduced* | Scaffold161:275917-299792 | 23,876 | 352 | 2 |
| *fushi tarazu* | Scaffold161:312168-313263 | 1,096 | 255 | 3 |
| *Antennapedia* | Scaffold161:385185-404098 | 18,914 | 367 | 2 |
| *Ultrabithorax** | Scaffold102:633776-637749 | 3,974 (partial) | 219  (partial) | 2 |
| *abdominal-A* | Scaffold102:452477-485096 | 32,620 | 394 | 5 |
| *Abdominal-B** | Scaffold102:165158-239410 | 74,253 (partial) | 238  (partial) | 1 or 3  (two isoforms) |
| *iroquois* | Scaffold36:225,058..310,105 | 85,048 | 806 | 7 |
| *mirror* | Scaffold36:113,167..159,432 | 46,266 | 591 | 5 |

##

# 2. Lateral Gene Transfers

*Contributed by John H. Werren, Sammy Cheng, Clauvis N.T. Taning, Dong Wei and Guy Smagghe*

## 2.1. Abstract

Lateral gene transfers (LGT) can contribute to adaptations in insects especially by including the ability to utilize plant resources. We have identified three very interesting LGTs in the *F. occidentalis* genome, which have the hallmarks of ancient lateral gene transfers into the insect genome that have subsequently undergone gene family expansions, and that show some signatures of having evolved function in the insect. These include two carbohydrate metabolism genes (mannanase and levanase) of bacterial origin, and an O-methyltransferase gene derived from bacteria.

### 2.2. Potential Bacterial Scaffold Detection

There were 102 potential bacterial contaminating scaffolds located in the genome sequence (**Additional file 2: Table S26**). The two largest contaminating bacterial scaffolds (scaffold 122 and scaffold 83) corresponded to parts of two bacterial associate genomes of *F. occidentalis* previously described in Facey et al., (Facey et al., 2015), BioProject PRJNA234511: Bfo1 ((SAMN03389132 ID: 3389132) and Bfo2 (SAMN03389135 ID: 3389135) respectively. Nucleotide sequences from this NCBI BioProject were found using targeted searches.

## 2.3. Lateral gene transfers in *F. occidentalis*

Growing evidence suggests that such LGTs can contribute to adaptations in insects, including the ability to utilize plant resources (Wybouw et al., 2016). We have focused our attention on three very interesting LGTs in the *F. occidentalis* genome, which have the hallmarks of ancient lateral gene transfers into the insect genome that have subsequently undergone gene family expansions with evolved functions in insects. These include two carbohydrate metabolism genes, mannanase and levanase; and an O-methyltransferase gene all derived from bacteria.

### 2.3.1. O-methyltransferase

O-methyltransferase is involved in methylation of small molecules and is known to affect diverse biological processes in bacteria, plants and animals, including cell signaling and catalytic activities (Liscombe et al., 2012). Originally found on scaffold 147, an O-methyltransferase also showed negligible similarity within insecta compared to the bacteria at both nucleotide and protein level. The sister group consisted of a bootstrap score of 85, which may suggest that Silvanigrellales bacterium could be a potential sister group to the O-methyltransferase. However, the bacterial source of the O-methyltransferase gene remains obscure, likely due to incomplete sampling of associated bacteria in insects. It clusters most closely with an O-methyltransferase gene sequence from the Silvanigrellales bacterium RF1110005 (bootstrap value 86, **Figure S2.1**) isolated from Lake Sanaru Japan; related sequences also come from environmental samples, such as a Bdellovibrionales bacterium assembled from a metagenomic sample obtained from the groundwater in Utah, USA.

Two other copies of the O-methyltransferase were found and all three can be seen to cluster together on the tree which indicates gene duplication events after transfer from the bacterium. Out of the three copies of O-methyltransferase, none show significant purifying selection by BUSTEC analysis (Table S3.1) compared to either a neutral or positive selection background. However, one copy (XP_026277179.1) shows significant positive selection. Examination of the NCBI Transcriptome Sequence Assembly database (<https://www.ncbi.nlm.nih.gov/genbank/tsa/>) for Thysanoptera taxid:30262 by tblastn detected the O-methyltransferase LGT in diverse species, including *Thrips palmi*, *Orothrips kelloggi*, *F. occidentalis*, *Franklinothrips vespiformis*, *Megalurothrips sjostedti* and *Gynaikothrips* (Phlaeothripidae). The distribution indicates that this LGT occurred prior to the divergence of the thrips suborders Terebrantia and Tubulifera approximately 260 MYA (Johnson et al., 2018).

### 2.3.2. Mannanase

Mannanase in bacteria hydrolyzes the endo- β1,4 glycosidic bond in carbohydrates (Wang, 2013). The mannanase was originally found on scaffold 197 and homology searched showed high similarity to bacterial references at both nucleotide and protein level. There were two other copies of the gene indicating subsequent gene duplications, based on their phylogenetic position (**Figure S2.2**). The *F. occidentalis* mannanase proteins are clearly embedded among bacterial sequences, although the actual sister group cannot be confidently identified due to low bootstrap support for adjacent bacterial sequences in the tree. The BUSTEC test for all three mannanase genes showed significant purifying selection p-value of 0.0066, 0.0000, 0.0016 for XP_026276666.1, XP_026285291.1, and XP_026285289.1 respectively, with no significant directional selection indicated for any of the branches (**Table S2.1**). The dN/dS values are consistent with maintenance of the open reading frame during purifying selection. Examination of the NCBI TSA database detected homologs of this mannanase only in *F. occidentalis*, *F. cephalica*, *Thrips palmi*, and *Megalurothrips sjostedti*, all members of the Thripidae. The distribution suggests that the LGT was acquired after divergence of the family Thripidae, approximately 175 MYA (Johnson et al., 2018).

### 2.3.3. Levanase

Levanase (GH32) is involved in sugar metabolism (Wanker et al., 1991). Initially, scaffold 31 and scaffold 54 indicated a levanase similar bacterial genes with a high homology to bacterial nucleotide references. Protein blasts of the thrips levanase LGT also gives very strong matches to bacterial proteins, particularly Streptomyces and Massilia genus. Matches to insect references are sporadically distributed indicating likely independent LGTs. In addition, the other insect candidates match to different bacterial sources than the levanase found in the thrips, suggesting that they are likely derived from independent LGTs. For example, an ancient LGT of a bacterial levanase derived from genus *Bacillus* has been found in Lepidoptera has been described (Sun, B. F. et al., 2013). The data suggest that levanases may be prone to retention and functional evolution after lateral transfer.

Reconstruction of the phylogenetic tree for the thrips levanase reveals that the two LGTs cluster together in the same clade, and therefore are paralogs that duplicated after the ancestral LGT event (**Figure S2.3**). The phylogenetic reconstruction after removal of bacterial gene duplicates suggests the sister group to be Streptomyces *sp*, however, the origin of the LGT cannot be unambiguously resolved at this time. BUSTEC test on the 2 LGT duplicates showed both have undergone significant purifying selection, while neither showed significant positive selection on the BUSTED test (**Table S2.1).** Examining the NCBI TSA database for Thysanoptera by tblastn reveals that this LGT is of ancient origin. It is found in members of the two major suborders of Thysanoptera (Terebrantia and Tubulifera), supporting the conclusion that the levanase was acquired prior to their divergence approximately 260 MYA (Johnson et al., 2018). Examples include *F. occidentalis*, *F. cephalica*, *Thrips palmi*, *Phlaeothripidae sp*. AD-2014, *Megalurothrips sjostedti*, and *Orothrips kelloggi*.

**Figure S2.1: Phylogenetic analysis of *Frankliniella occidentalis* O-methyltransferase**

**
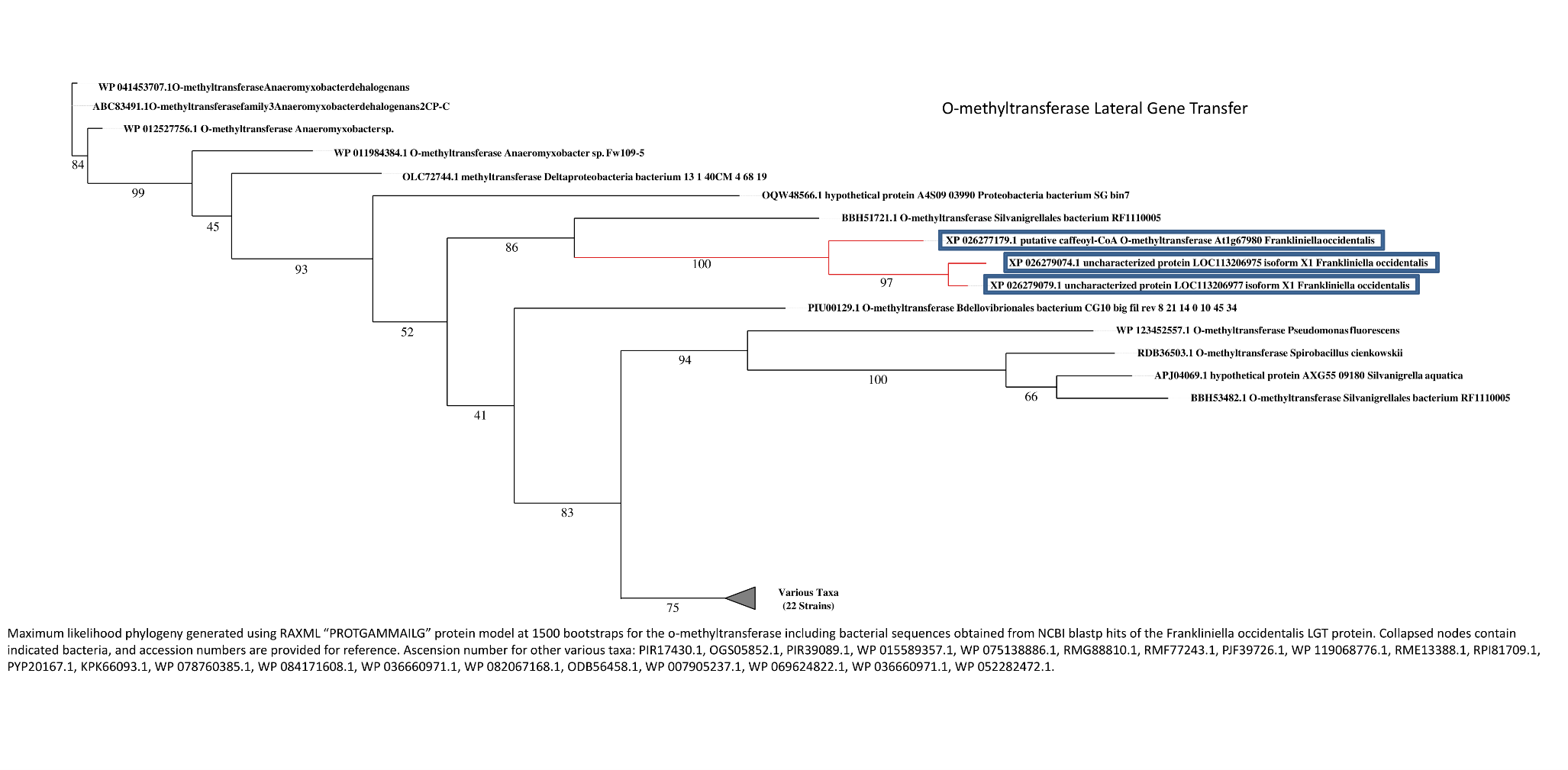
**

**Figure S2.2: Phylogenetic analysis of *Frankliniella occidentalis* mannanase**

**
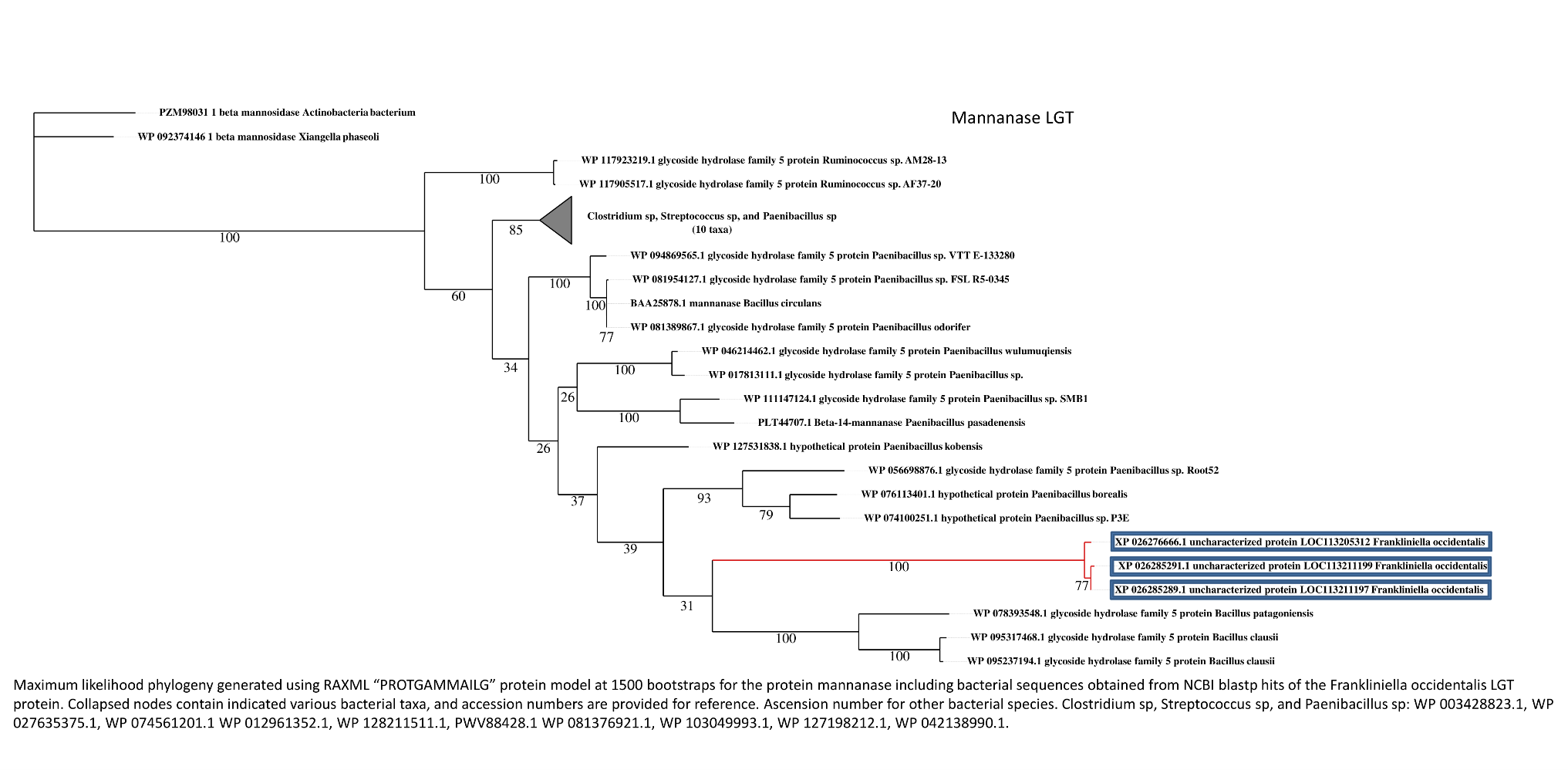
**

**Figure S2.3: Phylogenetic analysis of *Frankliniella occidentalis* Levanase**

**
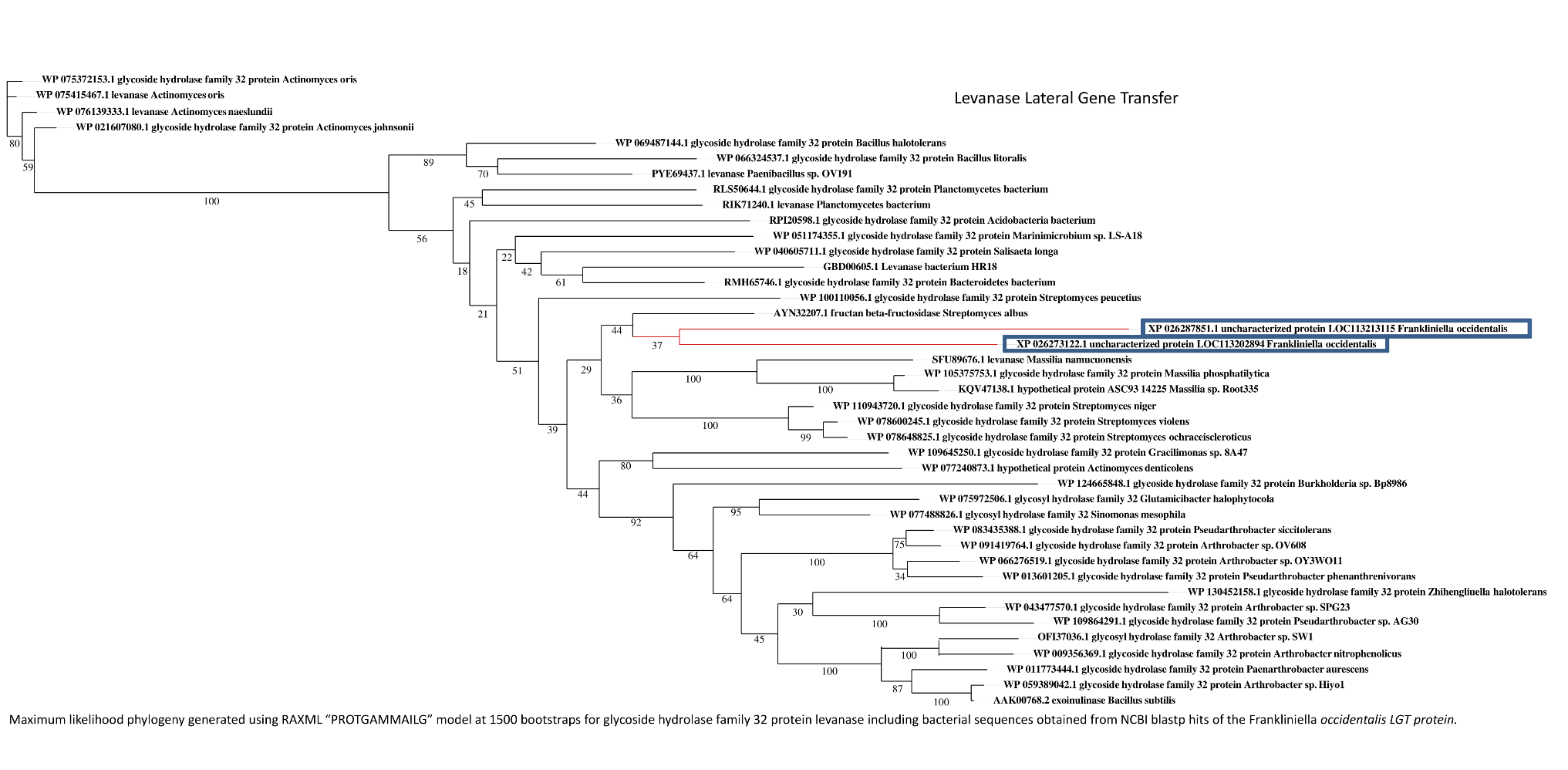
**

**Table S2.1: Lateral Gene Transfers identified in *Frankliniella occidentalis* genome**

| **Scaffold** | **NCBI protein sequence accession** | **BUSTEC-Purifying Selection (P-value)** | **BUSTED Positive Selection (P-value)** | **dN/dS** | **dN** | **dS** | **Expression (FPKM)^1^** | | |
| --- | --- | --- | --- | --- | --- | --- | --- | --- | --- |
|  |  | **Root to Tip** | | | | | **L1** | **P1** | **Adult** |
| **Levanase (glycoside hydrolase)** | | | | | | | | | |
| **Scaffold54** | XP_026287851.1 | Yes (0.0000) | No | 0.2249 | 0.2385 | 1.0608 | 0.68 | 1.5 | 119.2 |
| **Scaffold31** | XP_026273122.1 | Yes (0.0002) | No | 0.1866 | 0.1604 | 0.8595 | 9.17 | 0.16 | 9.94 |
| **Mannanase (endoglucanase)** | | | | | | | | | |
| **Scaffold96** | XP_026276666.1 | Yes (0.0066) | No | 0.3421 | 0.1575 | 0.4604 | 1.1 | 0.04 | 0.88 |
| **Scaffold197** | XP_026285289.1 | Yes (0.0016) | No | 0.3406 | 0.1578 | 0.4633 | 0 | 0 | 0.08 |
| **Scaffold197** | XP_026285291.1 | Yes (0.0000) | No | 0.3425 | 0.1597 | 0.46623 | 11.77 | 0.05 | 3.9 |
| **O-methyltransferase family 3** | | | | | | | | | |
| **Scaffold147** | XP_026279074.1 | No | No | 0.3806 | 0.1865 | 0.4901 | 0.04 | 0 | 0 |
| **Scaffold147** | XP_026279079.1 | No | No | 0.4146 | 0.1852 | 0.4467 | 2 | 2.26 | 8.34 |
| **Scaffold3388** | XP_026277179.1 | No | Yes (0.000) | 1.2196 | 0.2878 | 0.2360 | 31.01 | 1.8 | 52.86 |

^1^Average normalized read counts (FPKM) across four biological replications (data obtained from Schneweis et al., 2017); L1 = first instar larvae, P1 = propupae, Adult = females + males combined. PAML calculations were performed to obtain dN/dS, dN, and dS values using free-model rate with fixed branch lengths corresponding to the condensed protein topology. BUSTEC/BUSTED (Branch-site unrestricted statistical test of episodic conservation/Diversification) were used to test for purifying and positive selection respectively.

The Newick trees presented on the following page provide the NCBI accession numbers for each species along with the corresponding branch lengths used in the PAML and BUSTED/BUSTEC analysis.

Protein trees in Newick format used for the PAML, BUSTEC, and BUSTED analysis with NCBI accession numbers and corresponding branch lengths (Example XP_026279074.1: *Frankliniella occidentalis*):

O-methyltransferase Newick Tree
(((((BBH51721.1: 0.47036, ((XP_026279074.1:0.07289, XP_026279079.1:0.03747):0.22977, XP_026277179.1:0.18174):0.48919):0.24424, ((((WP_015589357.1: 0.60390, WP_075138886.1: 0.66211): 0.72888, (RME13388.1: 0.94520, (WP_082067168.1: 0.000001, ODB56458.1: 0.32160): 1.459072): 0.13838): 0.200289, (WP_123452557.1: 0.66496, APJ04069.1: 0.73901): 0.24336): 0.20522, PIU00129.1: 0.52133): 0.12866): 0.49045, WP_011984384.1: 0.23201): 0.20078, WP_012527756.1: 0.02863): 0.03006, ABC83491.1: 0.000001);

Mannanase Newick Tree
(WP_117905517.1: 0.00393, WP_117923219.1: 0.00514, ((WP_027635375.1: 0.27339, (WP_074561201.1: 0.39715, (PWV88428.1: 0.08969, (WP_081376921.1: 0.11634, WP_127198212.1: 0.06994): 0.03972): 0.03192): 0.05979): 0.08440, ((WP_046214462.1: 0.21086, (WP_127531838.1: 0.17558, ((XP_026276666.1:0.01013, (XP_026285291.1:0.00636, XP_026285289.1:0.000001050002909):0.01242):0.67791, WP_095237194.1: 0.39440): 0.21436): 0.06690): 0.04961, (WP_094869565.1: 0.02827, (WP_081389867.1: 0.000001, BAA25878.1: 0.000001): 0.02837): 0.11136): 0.06989): 0.10941);

Levanase Newick Tree
(WP_075372153.1: 0.03347, (WP_076139333.1: 0.03176, (RLS50644.1: 0.52357, (((((AYN32207.1: 0.26418, (XP_026287851.1:0.97733,XP_026273122.1:0.691288):0.10137): 0.068262, (SFU89676.1: 0.61089, WP_110943720.1: 0.39446): 0.06659): 0.07510, ((WP_013601205.1: 0.52527, (((WP_011773444.1: 0.16421, AAK00768.2: 0.12909): 0.06585, WP_009356369.1: 0.28759): 0.22529, WP_109864291.1: 0.55667): 0.07961): 0.06082, WP_075972506.1: 0.44854): 0.31827): 0.06151, WP_100110056.1: 0.52549): 0.09109, WP_040605711.1: 0.51741): 0.09500): 1.31041): 0.03285);

**Table S2.2: Primer sequences that bridge the LGT candidate and flanking eukaryotic-like sequences.**

| **Scaffold** | **Distance from neighboring thrips gene (kb)^1^** | **PCR Primer ID** | **Fragment size (kb)** | **Primer sequence**  **(5^՚^ → 3^՚^)** | | **Amplification result** |
| --- | --- | --- | --- | --- | --- | --- |
| **O-methyltransferase family 3** | | | | | | |
| **Scaffold147** | 80 kb | 147.51 | 6.5 | TGTCTCTGAAACTGCCCA | positive | |
|  |  | 147.31 |  | ATGTAATGCCCGTCGTGT |  |  |
|  |  | 147.52 | 1.66 | CCAGTATTACAGTCGGGTTCTT | positive | |
|  |  | 147.32 |  | CTTGGCGTTCGTAAGTGAA |  |  |
| **Mannanase/endoglucanase** | | | | | | |
| **Scaffold197** | 35 kb | 197.F1 | 4.80 | ATGTTCGTTGAGGTGGTGC | negative | |
|  |  | 197.R1 |  | TTGGTTGGTCAGAGGATGC |  |  |
|  |  | 197.F2 | 3.67 | AAGAAGAAGGCAAAGAGCG | positive | |
|  |  | 197.R2 |  | TAGACCAACCCGTCAATGA |  |  |
| **Levanase (Glycoside hydrolase)** | | | | | | |
| **Scaffold31^2^** | 300k | 31.F1 | 4.56 | GAGCAGCCCTATCAGTGTT | positive | |
|  |  | 31.R1 |  | GATGGCGTAGATTTCAGAGA |  |  |
|  |  | 31.F2 | 3.65 | CTCGCTTATCAGGGTCTTTAC | positive | |
|  |  | 31.R2 |  | CTTCGTTCGTTCACTCATTC |  |  |
|  |  | 31.F3 | 3.63 | ACCAGTGAGGTCAACAATGA | positive | |
|  |  | 31.R3 |  | TGATTAGATGTGCGAGGAAC |  |  |
| **Scaffold54** | 45k | 54.F1 | 3.09 | CGGACCTAACTTCAGGAACA | positive | |
|  |  | 54.R1 |  | CGTGTGATGTGTGCGAAT |  |  |
|  |  | 54.F2 | 6.09 | GGTTTATCGGCACTGACCA | negative | |
|  |  | 54.R2 |  | CCTTTACTCGTCTTCCACCCT |  |  |
|  |  | 54.F3 | 2.87 | CACACACACACACACCTCAT | positive | |
|  |  | 54.R3 |  | GTGCTCTGCTTGAAGTTCC |  |  |

^1^Linkage between the putative LGT and neighboring scaffold sequence (linker) that putatively ‘connects’ the LGT to the nearest *Frankliniella occidentalis* (FOCC) gene model indirectly, and vice versa; however, the distances between the LGT and nearest FOCC thrips gene on the scaffold was too long to confirm direct linkage between the LGT and gene model.

^2^Linkage confirmed between LGT and nearest FOCC gene (zinc carboxypeptidase-like), complete confirmation

# 3. **Chemosensory receptors**

*Contributed by Hugh M. Robertson*

## 3.1. Gene set manual annotations – customized strategy and phylogenetic analysis

The three major chemoreceptor gene families were manually annotated as for several other hemipteroids (Armisén et al., 2018, Benoit et al., 2016, Kirkness et al., 2010, Mesquita et al., 2015, Panfilio et al., 2019, Smadja et al., 2009, Terrapon et al., 2014) and many other insects and other arthropods using the Apollo genome browser at the i5k Workspace@NAL (Poelchau et al., 2015). Sensitive TBLASTN searches (E = 1000 and Word Size = 2) with other hemipteroid proteins as queries were used to identify loci and gene models built using a combination of the V0.5.3 gene models as well as Augustus and Snap models, supported by some spliced RNAseq reads, and knowledge of the expected gene structures supported by splice site predictions from the Splice Site Prediction by Neural Network webserver at the Berkeley Drosophila Genome Project website (http://www.fruitfly.org/seq_tools/splice.html). Iterative searches with each newly identified gene/protein were employed to search exhaustively for additional members of each family. Encoded proteins, including conceptual translation of pseudogenes using Z for stop codons and X for frameshifts and intron boundary mutations, were aligned with those from other hemipteroids and *D. melanogaster* representatives using ClustalX v2.0 (Larkin et al., 2007), and gene models were refined in light of these alignments. The final alignments included the families from the human body louse *Pediculus humanus* (Kirkness et al., 2010), the pea aphid *Acyrthosiphon pisum* (Smadja et al., 2009), and the bedbug *Cimex lectularius* (Benoit et al., 2016). The IR family was not described in these publications for the first two species, but was partially described in Croset et al. (Croset et al., 2010) with additional genes and refinement of gene models provided in Terrapon et al. (Terrapon et al., 2014). Additional available protein sets for these three families from the assassin bug *Rhodnius prolixus* (Mesquita et al., 2015), the milkweed bug *Oncopeltus fasciatus* (Panfilio et al., 2019) and a waterstrider *Gerris buenoi* (Armisén et al., 2018) do not contribute to the diversity of receptors known from heteropterans beyond those of the bedbug, so were not included in this analysis. Representatives of conserved proteins from *D. melanogaster* (Benton et al., 2009, Robertson et al., 2003), as well as a few other endopterygote species, were including in the GR and IR analyses for comparison. The alignments were trimmed to remove regions with mostly gaps using TrimAl v1.4 (Capella-Gutiérrez et al., 2009), with the “gappyout” option for the OR and GR families that have generally uniform lengths, and the “strict” option for the IRs, which vary enormously in the length and sequence of their N-terminal regions, effectively removing most of this from the alignment. Phylogenetic analysis was performed using maximum likelihood analysis with PhyML v3.0 (Guindon et al., 2010) at the ATGC webserver (http://www.atgc-montpellier.fr/phyml/). The resultant trees were prepared with FigTree v1.4.2 (http://tree.bio.ed.ac.uk/software/figtree/) and Adobe Illustrator.

### 3.2. Gene modeling difficulties

This draft genome assembly for *F. occidentalis* has many gaps as well as some misassemblies. These caused considerable difficulties for modeling of these chemoreceptor genes, especially for sets of closely related genes as well as divergent genes that are not well enough expressed to have RNAseq reads in the available datasets, all of which are from whole animals. RNAseq support allowed resolution of some misassemblies, as well as discovery of exons missing in gaps, which were manually built using raw genome reads from the Sequence Read Archive (SRA) at the National Center Biotechnology Information. These three gene families were initially manually annotated in 2015, and revisited in 2018 with assistance from additional RNAseq information in the SRA. Nevertheless, many gene models remain incomplete, especially in large recent expansions such as the IR102-268 gene set. Examples of these difficulties are exemplified by the IR family detailed below, where among the first 15 intron-containing genes just four are intact in the assembly, (gene name suffixes are F – assembly repaired, J – model joined across scaffolds, N – N-terminus unidentified).

Ir8aF – Assembly has an 18bp deletion removing the front of the last exon, repaired with RNAseq and raw genomic reads

Ir21aJ – N-terminal and C-terminal exons on the ends of a large scaffold and a 3kb contig.

Ir25aF – Three internal exons missing, built from RNAseq and raw genomic reads

Ir40a – Intact.

Ir68aJ – N-terminal and C-terminal exons on the ends of two large scaffolds.

Ir76bF – Just three exons present, flanked by large gaps – RNAseq used to find four C-terminal exons not in assembly, plus single N-terminal exon misassembled downstream in next contig after a gap, supported by spliced RNAseq reads.

Ir93aJ – Mostly in a large scaffold, but N-terminal exons in a 19kb scaffold that must belong in a short gap within the large scaffold.

Ir75aNJ – Most exons are in a 16kb scaffold that apparently belongs in a gap in a much larger scaffold that has the final two exons. N-terminus missing in gap, but no RNAseq to help find it.

Ir75bJ – N-terminal and C-terminal exons are in middles of two large scaffolds, so some kind of misassembly, connection supported by spliced RNAseq reads.

Ir75cJF – Three central exons are in a 1kb scaffold that belongs in a gap in a much larger scaffold, and two exons in this larger scaffold are in inverted order.

Ir75d – Intact.

Ir75e – Intact.

Ir75fN – In a 16kb scaffold but N-terminal exon unidentified.

Ir7gNJ – Four central exons are in a 1kb scaffold that belongs in a gap in a much larger scaffold, but N-terminus not identified in absence of RNAseq.

Ir75hNJ – Joined across two scaffolds on basis of RNAseq. Can’t find N-terminal exon.

Ir101 – Intact.

Ir102-243 – Largely mostly intronless genes, but 52 (one third) have one or both ends missing in gaps, presumably because they are so similar to each other the assembly was unable to build them completely. There are many more fragments not included in the named genes.

Ir244-268 – Three-exon genes, and 4 have parts missing in assembly gaps.

### 3.3. The OR family

The OR family is unique to insects (Brand et al., 2018, Missbach et al., 2014, Robertson, 2019) and usually consists of a single Orco gene and a set of “specific” ORs that mediate specificity and sensitivity of most of insect olfaction (Joseph and Carlson, 2015). The expected single conserved Orco gene has the first two exons in a separate 1.6 kb scaffold that can confidently be placed in a gap within 1.4Mbp Scaffold63 based on both sequence conservation and spliced RNAseq reads. Amongst the 84 “specific” ORs, 43 are intact full-length genes (15 of which required repair of the assembly and one was joined across two scaffolds). Of the remaining 41 genes, just two are apparent pseudogenes, while the rest have parts missing in gaps, or might also be pseudogenes, but are assumed to be intact in the genome. As shown by the tree in **Figure S3.1**, all of these thrips ORs form a distinctive species-specific clade, commensurate with the generally rapid sequence divergence of ORs in insects and the phylogenetic divergence of thrips from other hemipteroid orders represented here. Like all other hemipteroid insects to date, no ligand specificities are known for any of the specific ORs, and their enormous divergence from those of endopterygote insects with known ligand specificities preclude any inferences of ligand specificity and hence specific roles in thrip biology, however they are inferred to mediate the specificity and sensitivity of most thrips olfaction, in particular sensing host plant volatiles (Cao et al., 2014, de Kogel, W.J. and Koschier, E.H, 2002, Koschier et al., 2000, Mainali and Lim, 2011, Silva et al., 2016, Teulon et al., 1993) as well as pollen for food (Abdullah et al., 2014). They likely also mediate perception of alarm (de Bruijn et al., 2006, Teerling et al., 1993), aggregation (Hamilton et al., 2005), and sex pheromones (De Kogel, Willem Jan and Van Deventer, 2003, Kirk and Hamilton, 2004, Olaniran et al., 2013).

**Figure S3.1. Phylogenetic relationships of the odorant receptors of hemipteroid insects.** The Orco lineage was declared the outgroup to root the tree, based on its basal position in the OR family in analyses of the entire insect chemoreceptor superfamily (Missbach et al., 2014, Robertson et al., 2003). The scale bar is substitutions per site, and the filled dots are approximate likelihood ratio test (aLRT) values from PhyML ranging from 0-1. The list of FOCC OGS v1.0 models for OR genes are found in **Additional file 2: Table S7.**

### 3.4. The GR family

The GR family is far older than the OR family, which evolved from within it (Brand et al., 2018, Missbach et al., 2014, Robertson et al., 2003), and has evolved multiple divergent lineages since its origin in basal animals (Eyun et al., 2017, Robertson, 2015, Robertson, 2019, Saina et al., 2015). The most prominent of these are the sugar and carbon dioxide receptor subfamilies, which are distantly related to each other. This thrips has a considerable expansion of genes in each of these two subfamilies, with 18 candidate sugar receptors and 30 members of the carbon dioxide receptor subfamily. The sugar receptors are characterized by a glutamic acid (E) after the TY in the conserved TYhhhhhQF motif of the transmembrane 7 domain replacing a normally hydrophobic amino acid (h in the motif) (Kent and Robertson, 2009). This position, by inference from the three- dimensional structure of Orco (Butterwick et al., 2018), is alongside the ion channel of the receptor tetramer. Of the 18 FoccGrs in the sugar receptor subfamily 16 have this TYE, the two exceptions being TYA and TYI in Gr37/38, respectively. It is unclear how this expansion of sugar receptors, compared for example with two in honey bees (Jung et al., 2015), 8 in *D. melanogaster* (Robertson et al., 2003) , 8-13 in mosquitoes (Kent and Robertson, 2009), and 16 in the flour beetle *Tribolium castaneum* (Richards et al., 2008), might be involved in their utilization of flowers as host plants, in part because we have yet to fully understand how the 8 Drosophila sugar receptors are deployed to sense diverse sugars (Fujii et al., 2015).

The large expansion of 30 genes in the carbon dioxide receptor subfamily is comparable to a similar expansion of this subfamily in the dampwood termite *Zootermopsis nevadensis* (Terrapon et al., 2014) and the German cockroach *Blattella germanica* (Robertson et al., 2018), but not all are expected to be involved in perception of this gas. FoccGr1-3 are most closely related to the Gr1-3 lineage of carbon dioxide receptors in endopterygote insects (Robertson and Kent, 2009) (**Figure S3.2**), and so might indeed detect this gas, while the others presumably represent the larger subfamily from which the carbon dioxide receptors evolved, a subfamily that is at least as old as odonates (Ioannidis et al., 2017).

A distinct lineage of GRs has evolved to detect the sugar fructose, exemplified by the Gr43a protein in *D. melanogaster* (Miyamoto et al., 2012). Like most insects, this lineage is represented in other hemipteroids by a single gene, however this thrips has five genes (Gr49-53), while the lineage is expanded up to 10 genes in *T. castaneum* (Richards et al., 2008). This gene lineage evolved from within a far larger evolutionary assemblage of GRs, most of which in *D. melanogaster* are implicated in detecting “bitter” compounds, typically from plants (Weiss et al., 2011).

The remaining 49 GRs in this thrips are highly divergent from the other hemipteroid GRs, perhaps consistent with a similar role of detecting “bitter” plant defensive compounds. They form three clades in the phylogenetic analysis (**Figure S3.2**), the largest consisting of 40 genes. The latter includes a recent expansion of GR54-67. This is not quite a complete catalog of the GR family in this genome, because despite successfully repairing the assembly for 22 genes, 23 genes remain incomplete models with exons missing in gaps, while two are clear pseudogenes. At least 8 more fragments that might well represent intact genes in the genome were detected, but not named and analyzed as they could not be built into reasonable length models.

**Figure S3.2. Phylogenetic relationships of the gustatory receptors of hemipteroid insects.** The sugar and carbon dioxide receptor subfamilies were together declared the outgroup to root the tree, based on their position in phylogenetic analysis of the GR family throughout animals (Robertson, 2015). The three major subfamilies are highlighted with background colors. Other details as for Figure S3.1. The list of FOCC OGS v1.0 models for GR genes are found in **Additional file 2: Table S7.**

### 3.5. The IR family

The ionotropic receptor family is a divergent lineage of the ancient family of ionotropic glutamate receptors (Benton et al., 2009) and evolved in protostomes (Croset et al., 2010, Eyun et al., 2017). The family has been reviewed recently (Rimal and Lee, 2018, Rytz et al., 2013). In most insects there are two highly conserved co-receptors, named Ir8a and 25a for their *D. melanogaster* orthologs, with lengths and sequences very similar to the glutamate receptors. A third co-receptor is the far shorter Ir76b protein. In addition, *D. melanogaster* has four proteins expressed in antennae that mediate perception of temperature and humidity in conjunction with some of the coreceptors, Ir21a, 40a, 68a, and 93a (Knecht et al., 2016). Most insects have single orthologs for each of these seven genes, and this thrips is no exception (**Figure S3.3**). In addition there is a clade of receptors known as the Ir75 clade with seven members in *D. melanogaster*, most of which are involved in perception of acids and amines (Prieto-Godino et al., 2017), that is also commonly expanded in other insects, usually independently of the fly expansions. *F. occidentalis* has 6 members of this clade, all independently duplicated not only from the fly genes, but from the relatives in other hemipteroids (Figure S3). As described above, just three of these 14 genes were intact in the genome assembly, the remainder requiring repairs to the assembly to encode full-length proteins. Many insects including more basal lineages like termites (Terrapon et al., 2014) and cockroaches (Robertson et al., 2018) also have relatives of the Ir41a and related proteins in *D. melanogaster*, however this thrips does not.

The remaining IRs in *D. melanogaster* form a divergent grouping, most of which fall into a large clade called the IR20a clade, and these have been implicated in gustation in both larvae and adults (Koh et al., 2014, Sánchez-Alcañiz et al., 2018, Stewart et al., 2015). In other insects these divergent IRs typically fall into two groups, a group with several introns and an “intronless” clade, and post Croset et al. (Croset et al., 2010), these genes have been given numbers from 101 upwards (e.g. (Robertson et al., 2018, Terrapon et al., 2014)) to avoid confusion with the *D. melanogaster* genes which are numbered up to 100a because they were named for their cytological locations (Benton et al., 2009). This thrips has just one of the multiple-intron genes, Ir101, compared with a handful in the other hemipteroids (whose sequences for *P. humanus* and *A. pisum* were updated in (Terrapon et al., 2014)). The remaining “intronless” IRs form a greatly expanded thrips-specific clade of at least 167 genes, many of which are partial models with parts missing in assembly gaps, and there are many more small fragments in the assembly that might represent intact genes in the genome. Eight of these are clearly pseudogenic. This expansion is comparable to one of 93 “intronless” genes in *Z. termopsis* (Terrapon et al., 2014), and 755 in *B. germanica* (Robertson et al., 2018). A comparable large expansion of IRs was found in the deer tick *Ixodes scapularis* (Josek et al., 2018). A few of these genes do have one and sometimes two introns, however these have been idiosyncratically gained at different locations after the expansion of the clade from an intronless ancestor. By analogy with *Drosophila* flies, these IRs are likely to function in gustation, and like the divergent GRs, might be involved in perception of diverse host plant chemicals.

**Figure S3.3.** **Phylogenetic relationships of the ionotropic receptors of hemipteroid insects.** The Ir8a and 25a lineages were together declared the outgroup to root the tree, based on their close relationship to the ancestral glutamate receptors (Croset et al., 2010, Eyun et al., 2017, Terrapon et al., 2014). The major conserved lineages are highlighted with background colors. Other details are similar to Figure S4.1. The list of FOCC OGS v1.0 models for IR genes are found in **Additional file 2: Table S7.**

# 4. Vision genes

*Contributed by Markus Friedrich and Jeffery W. Jones*

## 4.1 Background

Vision supports many aspects of insect biology and is mediated by a variety of light-sensing receptor proteins. In thrips, vision has been shown to be involved in plant host finding. This capacity is most likely mediated by homologs of the opsin gene family, expressed in the photoreceptors of the moderately sized pair of compound eyes in thrips. Opsins constitute a deeply conserved class of light-sensitive protein G-protein coupled receptors. Three major subfamilies have been identified across the animal kingdom: Ciliary opsins (c-opsins), rhabdomeric opsins (r-opsins) and RGR/Go opsins (Cronin and Porter, 2014). R-opsins have most extensively diversified in insects (Cronin and Porter, 2014, Feuda et al., 2016, Hering et al., 2012), resulting in paralog groups with differential wavelength sensitivity maxima. This includes the long wavelength-sensitive subfamily (LWS-opsin), a blue or short wavelength sensitive opsin (SWS-B-opsin), and the ultraviolet-short wavelength sensitive opsin (SWS-UV-opsin), all of which are mainly expressed in the photoreceptors of the peripheral visual system, i.e. the compound eyes and the median eyes. In addition, the r-opsin subfamily also includes paralog groups whose members have been found to be predominantly expressed in non-retinal tissues: Rh7 opsins and Arthropsins (Eriksson et al., 2013, Ni et al., 2017). In some species, the extraretinal r-opsins are complemented by the presence of homologs of the c-opsin subfamily in insects, which are likewise expressed in non-retinal tissues (Velarde et al., 2005). Further light-sensitive proteins expressed in non-retinal tissues includes cryptochromes and photolyases (Porter, 2016).

## 4.2 Gene set manual annotations – customized strategy and phylogenetic analysis

We searched the *F. occidentalis* genome draft in the i5k Workspace by tBLASTn using the protein sequences of characterized light sensitive genes from *Drosophila* and the red flour beetle *Tribolium castaneum* as queries (Altschul et al., 1990, Poelchau et al., 2015). For *Drosophila*, this included (6-4)-photolyase (phr6-4: CG2488), cryptochrome (cry: CG3772), Rhodopsin-1 (Rh1: CG4550), Rhodopsin-2 (Rh2: CG16740), Rhodopsin-3 (Rh3: CG10888), Rhodopsin-5 (Rh5: CG5279), Rhodopsin-6 (Rh6: CG5192), and Rhodopsin-7 (Rh7: CG5638). For *Tribolium*, this included cryptochrome 2 (cry2: XP_008200421) and ciliary opsin (c-opsin: XP_001816446). Orthology relationships of candidate homologs were scrutinized by reciprocal BLAST (Wall et al., 2003). For members of the opsin gene family, subfamily relationships were explored by gene tree reconstruction. Protein sequences were aligned with Clustal Omega (Sievers et al., 2011). Ambiguous alignment regions were filtered using Gblocks applying least stringent setting (Castresana, 2000). Bootstrapped maximum likelihood tree was estimated with RAxML as implemented on the CIPRES platform (Miller et al., 2010, Stamatakis, 2014).

## 4.3 Vision genes

Our searches detected seven opsin genes, two cryptochromes, and a singleton homolog of phr6-4 in the *F. occidentalis* genome (**Additional File 2: Table S8**). The opsin gene homologs represented five homologs of subfamilies expressed in the peripheral visual system, which included singleton homologs of each the UV- and B-opsin subfamily, complementing three tandem-duplicated LW opsins on scaffold 18. Gene tree analysis revealed that the *F. occidentalis* LW opsin cluster represents an independent expansion of LW opsins in relation to the LW opsin clusters found in hemipteran species (**Fig. S4.1**) (Armisén et al., 2018, Panfilio et al., 2019, Sparks et al., 2020). In addition to these opsins that are most likely expressed in the peripheral visual system, we detected singleton homologs of c-opsin (Velarde et al., 2005) and the Rh7 opsin (Ni et al., 2017). We failed to detect sequence conservation evidence for Arthropsins in *F. occidentalis* (Eriksson et al., 2013), although this opsin gene family has been found in a variety of hemipteran species (Armisén et al., 2018, Panfilio et al., 2019, The International Aphid, Genomics Consortium, 2010).


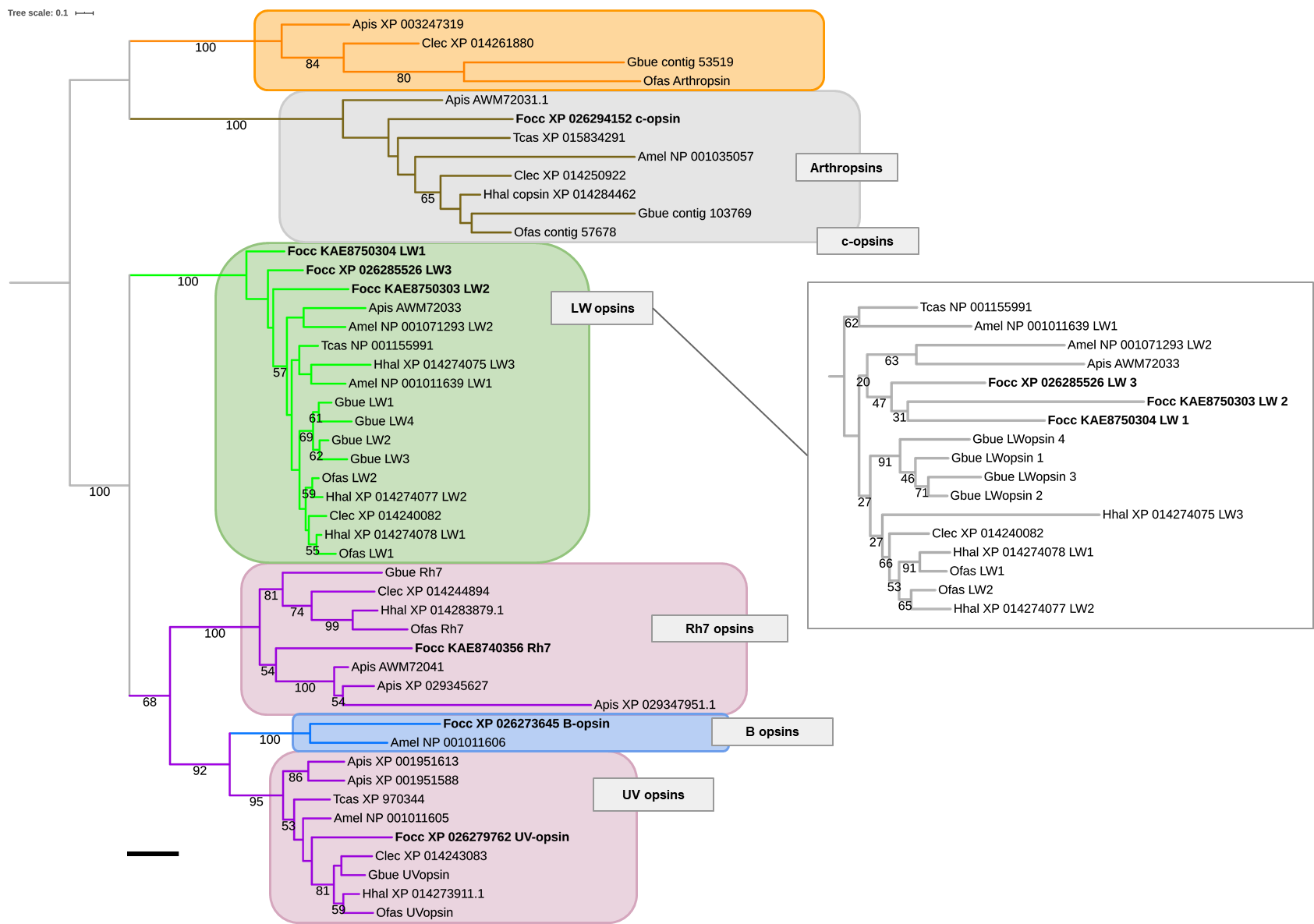


**Figure S4.1: Nonparametric bootstrap maximum likelihood tree of relationships between opsin homologs from hemipteran and other arthropod species.** Species abbreviations: Amel = *Apis mellifera*, Apisum = *Acyrthosiphon pisum*, Clec = *Cimex lectularius*, Focc = *Frankliniella occidentalis*, Gbue = *Gerris buenoi*, Hhal = *Halyomorpha halys*, Ofas = *Oncopeltus fasciatus*, Tcas = *Tribolium castaneum*. Sequences and alignment available on request. Numbers at branches represent non-parametric bootstrap support higher than 75. Scale bar corresponds to 0.2 substitutions per site. Inset shows LW opsin gene tree reconstructed with LW opsins only and rooted by the Tribolium and honeybee LW opsin homologs that are expressed in the main retina. Note moderate the support for a monophyletic *F. occidentalis* LW opsin gene cluster.

# 5. Validation of salivary gland-enriched transcripts

*Contributed by Sulley Ben-Mahmoud, Joshua Benoit, Dorith Rotenberg and Diane Ullman*

## 5.1. Abstract

The salivary glands (SG) of the *Frankliniella occidentalis* (Pergande), the western flower thrips, are critical to the insect’s competence in transmitting viruses in the genus *Orthotospovirus,* including the type member*, Tomato spotted wilt virus* (Montero-Astuá et al., 2016). Sequencing of the *F. occidentalis* genome provides an exciting opportunity to reveal, at the molecular level, how salivary gland components function in insect-plant and insect-virus interactions, including identification of effectors and proteins involved in virus replication, retention and transmission. Comparison of RNAseq data from *F. occidentalis* SGs and whole bodies identified 123 genes expected to be enriched in the salivary gland (**Additional file 2: S11**). We present evidence validating that four of these genes [Maker IDs: FOCC009158 (F9158), FOCC007547 (F7547), FOCC003700 (F3700), and a pancreatic triacylglycerol lipase-like gene FOCC003652 (F3652)] are enriched in the SG of adult female *F. occidentalis*, relative to the insect head or body. These genes are predicted to be secretory proteins. These findings are an important step towards elucidating organ-specific functions of genes in *F. occidentalis*.

## 5.2. Results and Discussion

*Frankliniella occidentalis* females were obtained from a colony originally collected from the same Hawaiian isolate used in this study, and maintained on green bean pods as described in Ullman et al., 1992. Using primer pairs reported in **Table S5.1** for real-time quantitative reverse transcription-PCR analysis of expression, comparisons of mean normalized transcript abundance in the head, body, and salivary glands for four genes predicted to be enriched in the salivary gland (F9158, F7547, F3700 and F3652) showed that transcript abundance was always significantly higher in the salivary gland than the head or body **(Figure S5.1)**. One gene, F3700 was detected solely in the SG (**Figure S5.1C)**. Among the three genes that were also expressed in the head and body, F9158 and F3652 did not differ statistically between the head and body, while transcript levels in the salivary gland were significantly higher (*P <* 0.001) (**Figure S5.1A and S5.1D)**. F7547 was differentially expressed in the head, body, and salivary gland with the lowest mean transcript abundance in the head, followed significantly higher abundance in the body, and then the highest abundance in the salivary gland (*P <* 0.05) (**Figure S5.1B)**.

**Table S5.1.** Primers used for real-time quantitative reverse-transcription PCR^a^ of four candidate SG transcripts in *F. occidentalis.*

| **Gene Notation in Figure S5.1** | **Maker ID** | **Annotation** | **Primer ID** | **Sequence (5' - 3')** | **Primer pair efficiency (%)** | **Expected amplicon size (bp)** |
| --- | --- | --- | --- | --- | --- | --- |
| Actin^b^ | F0CC008191 | FoccTmpA008191-RA | FoccAct1_f FoccAct1_r | GGTATCGTCCTGGACTCTGGTG GGGAAGGGCGTAACCTTCA | 98 | 69 |
| COX^b^ | FOCC005842 | FoccTmpM005842-RA | FocCOX_f FocCOX_r | CGTTACCAGTTTTAGCAGGAG TCCTCTCGGATCAAAGAAGG | 102 | 80 |
| F9158 | FOCC009158 | Putative SG Protein 21 | Foc26Srs3_f Foc26Srs3_r | CCACTGAAGACCTGACTGAT ATAACGTGTTCCTTGGGAGT | 107 | 109 |
| F7547 | FOCC007547 | Putative SG Protein 22 | Foc7547_f Foc7547_r | GAACTGTGACCATGTCTGTG CTTGGTGGTGATGTTCTTGG | 102 | 80 |
| F3700 | FOCC003700 | Putative SG Protein 23 | Foc3700b_f Foc3700b_r | AGAGGAAAAGAAGGACAGCG CAGAAGAGAGGGGATTGCTG | 103 | 80 |
| F3652 | FOCC003652 | Pancreatic triacylglycerol lipase-like | Foc3652a_f Foc3652a_r | CGGCGGCATATTAGGAATTG CTCAATGGCGTTGATGTACG | 100 | 148 |

^a^Refer to methods in the FOCC genome paper for sample preparation, RNA isolation, cDNA synthesis and real-time qRT-PCR parameters, protocols and analysis of gene expression (Livak and Schmittgen, 2001)

^b^actin and cytochrome oxidase subunit 1 (COX) served as internal reference genes (transcripts) to normalize SG candidate transcript expression (Boonham et al., 2002; Wang et al., 2014); E = PCR efficiency of the primer pair

**Fig. S5.1. Transcript abundance of SG enriched genes in the head, body and salivary glands of female adult *Frankliniella occidentalis.*** Transcript abundance (normalized with actin (Act) and cytochrome oxidase I (COX)) of SG enriched genes with original maker model ID: FOCC009158-RA (F9158), FOCC007547-RA (F7547), FOCC003700-RA (F3700), and FOCC003652-RA (F3652) was determined for six replicates each of total RNA extracted from the head, body and salivary glands of pools of five 48-hour old female adult WFTs. Symbols (replicates) of the same shape for all SG enriched gene represent the same group of 5 female adults. Lines represent the mean of the six replicates ± the standard error of the mean (SEM). One-way ANOVA: Tukey’s multiple comparison tests, revealed significant differences in the abundance in the SG relative to the head, and body, in all four genes.

These data support our comparative analysis of the sialotransciptome, whole body transcriptome and *F. occidentalis* genome sequence databases, providing strong additional evidence that F9158, F7547, F3700 and F3652 are enriched, or more highly expressed in the salivary gland than other parts of the thrips body. For every respective biological replicate (pool of 5 female adults), and for every gene quantified in this study, the transcript abundance value (normalized against actin and cytochrome oxidase) was highest in the salivary gland. In the case of F3700, there was negligible expression in the head, and the body as the qRT-PCR primers designed to measure this gene failed amplify the expected product in these body regions. To control for possible starvation effects, we gave thrips access to green bean pods *ad libitum,* but we were not certain if each individual was fully fed before it was dissected.

Several predictive tools: SignalP4.1, Phobius, and MultiLoc (Höglund et al., 2006, Käll et al., 2007, Petersen et al., 2011), suggest that all of the four genes could be secreted proteins playing important function in thrips-plant interactions, as they have signal peptides at the N-terminal region of their sequences. Their potential importance in this regard drove our choice to use these genes for validation of SG-enriched genes. Our bioinformatics analysis designated F3652 as a pancreatic triacylglycerol lipase-like gene and one of the genes likely to be involved in feeding or digestion **(Table S11, Excerpt E)**. While we do not know its function in thrips, a pancreatic-lipase of unknown function was also found in the SG of the mosquito *An.* Stephensi (Valenzuela et al., 2003). Notably, this mosquito species is known to feed on plant-sugars. Members of the pancreatic lipase gene family are predicted to hydrolyze galactolipids (Aoki et al., 2007) which make up the bulk of photosynthetic membranes (Dörmann, 2013), and may help the insects to access plant sugars. As *F. occidentalis* feeds on parenchymal and mesophyll cells rich in such membranes **(plant cells are shown in Fig. 1C of FOCC genome paper)**, F3652 may play an important role in feeding, digestion and plant defense. Analysis of F9158, F7547 and F3700 showed that these genes did not encode proteins that match known proteins from any other organism. While the signal peptides at the N-terminal region of their sequences suggest they are secreted and as such may play a role as effectors in plant defense or in other feeding or digestive roles, we do not have any other indications to suggest their actual functions (see details about their expression and location by searching for their Maker IDs in Table S11).

This validation of SG-enrichment supports our selection of 123 SG-enriched genes and supports future studies on how the salivary proteins these genes encode may manipulate plant defenses and processes surrounding TSWV retention and replication in *F. occidentalis*, as well as inoculation to plants. SG-enriched proteins may enable entry and egress of tomato spotted wilt virus components into/out of the SG. Furthermore, the secreted proteins may function to help this thrips circumvent plant defenses, and TSWV to colonize plant tissues (Hogenhout et al., 2009). As high-throughput screening methods are developed for use with thrips, this analysis of SG-enriched genes will provide a foundation for deeper functional annotation and understanding

# 6. Detoxification genes

## 6.1. Cytochrome P450s

*Contributed by Jonathan Oliver, Derek Schneweis, Dorith Rotenberg and Anna Whitfield*

*Special thanks to David Nelson, University of Tennessee, USA, for kindly assigning CYP nomenclature and clan to each gene model using his in-house pipeline*

### 6.1.1. Abstract

Cytochrome P450s (P450s, CYPs) are a large superfamily of enzymes. P450 enzymes have been identified in all domains of life where they are involved in the metabolism of multiple substrates with prominent roles in hormone synthesis and breakdown, development, and detoxification (Feyereisen, 1999, Heidel-Fischer and Vogel, 2015). In agricultural systems, *F. occidentalis* has shown a propensity for developing resistance to insecticides commonly utilized to manage this species, and P450s have been specifically implicated in the detoxification of insecticides by *F. occidentalis* (Cifuentes et al., 2012, Yan et al., 2015). Within the *F. occidentalis* genome, a relatively large number of P450s were identified, including numerous members of CYP families frequently associated with the breakdown of toxic plant products and insecticides (Cifuentes et al., 2012).

## 6.1.2. Gene set manual annotations – customized strategy and phylogenetic analysis

P450s in the genome of *F. occidentalis* were identified based upon similarity to known P450s in other insect species. Initially, 130 P450 gene models were annotated across 88 different scaffolds (Additional file 2: Table S13), with clustering of P450 genes on some scaffolds as has been noted to occur in other insect genomes including *D. melanogaster* and *T. castaneum* (Chung et al., 2009, Zhu et al., 2013). Based upon alignments with other insect protein orthologs and *de novo* transcriptome evidence from RNAseq (Schneweis, 2017), these 130 CYP gene models were determined to represent at least 89 unique CYP gene sequences (some fragmented across different scaffolds) as well as 23 additional partial sequences. A CYP gene was counted as "complete" that: (1) started with methionine, (2) had the domains necessary to be recognizable as a CYP using blastp, and (3) was approximately as long as a CYP should be (so greater than ~350 amino acids. P450 genes were assigned to families and named on the basis of overall amino acid sequence identity using the blastp function – with P450s showing greater than 40% amino acid identity receiving the same number designation (CYP family) and greater than 55% identity receiving the same letter designation (CYP subfamily) (Nelson, 1998) through comparisons with annotated CYPs from the genomes of other insects (including *Drosophila melanogaster*, *Pediculus humanus corporis*, *Tribolium castaneum*, *Acyrthosiphon pisum*, *Cimex lectularius*, *Zootermopsis nevadensis*, and *Diaphorina citri*)*.* CYP gene sequence classifications (family and clan) were independently confirmed or modified accordingly by D. Nelson using his up-to-date in-house pipeline on July 5, 2019. Those family and clan assignments are listed in Table S6.1 below and in Additional file 2: Table S13 for each CYP sequence.

Amino acid sequence alignments and phylogenetic analysis was performed on the *F. occidentalis* CYP genes (97 in the alignment) alongside the most similar P450 sequences (388 in total) from other insect species found in GenBank (https://www.ncbi.nlm.nih.gov/genbank/) as of 8-25-16 using MEGA X (Kumar et al., 2018) (refer to Figure S6.1 for analysis parameters). The FOCC OGS v1.0 gene models and other insect species P450 GenBank accessions included in the resulting tree are listed in Additional file 2: Table S14.

### 6.1.3. Results and Discussion

Characterization of the unique P450s from *F. occidentalis* indicated that representatives of at least 24 different CYP families are present in the genome (**Table S6.1**). Overall, more than 40% of the total number of CYP genes annotated in the *F. occidentalis* genome were assigned to the CYP4 and CYP6 gene families. Phylogenetic characterization of *F. occidentalis* P450s versus those found in other insect species has been completed (**Figure S6.1**), and phylogenetic analysis suggested expansion within the CYP 3 and CYP 4 clans of *F. occidentalis*. The majority of annotated *F. occidentalis* P450s showed relatively low identity to other insect P450s. This is in agreement with the findings of Scott and Wen (Scott and Wen, 2001) that the majority of P450s in insect genomes show very limited amino acid identity (30-50%) to P450 genes in other insect species.

All told, a diverse array of P450s were annotated within the *F. occidentalis* genome. Given the already described importance of P450s in insecticide resistance (Cifuentes et al., 2012, Yan et al., 2015), the importance of insecticides in the management of thrips species (Cifuentes et al., 2012), and the multitude of plant defense compounds encountered during the thrips’ phytophagous lifestyle (Heidel-Fischer and Vogel, 2015), knowledge of the diversity of P450s present within the *F. occidentalis* genome is likely essential for optimizing management of this important agricultural pest. The annotation of these P450 genes will enable future functional studies in *F. occidentalis* related to the detoxification of insecticidal and plant defense compounds.

**Table S6.1** Family assignments for annotated *F. occidentalis* P450 genes. Families in bold are new families, thrips-specific.

| Clan | Family | Total | Complete | Partial |
| --- | --- | --- | --- | --- |
| 2 | CYP15 | 3 | 3 | 0 |
| 2 | CYP18 | 1 | 1 | 0 |
| 2 | CYP303 | 1 | 1 | 0 |
| 2 | CYP304 | 3 | 3 | 0 |
| 2 | CYP305 | 1 | 1 | 0 |
| 2 | CYP306 | 1 | 1 | 0 |
| 2 | CYP307 | 2 | 2 | 0 |
| 3 | CYP6 | 26 | 20 | 6 |
| 3 | **CYP3652** | 1 | 1 | 0 |
| 3 | **CYP3653** | 2 | 2 | 0 |
| 3 | **CYP3654** | 1 | 1 | 0 |
| 4 | CYP4 | 20 | 16 | 4 |
| 4 | **CYP3655** | 15 | 11 | 4 |
| 4 | **CYP3656** | 2 | 2 | 0 |
| 4 | **CYP3657** | 9 | 8 | 1 |
| 4 | **CYP3658** | 4 | 3 | 1 |
| 4 | **CYP3659** | 2 | 1 | 1 |
| 4 | **CYP3660** | 1 | 1 | 0 |
| 4 | **CYP3661** | 3 | 0 | 3 |
| 4 | Unassigned | 1 | 0 | 1 |
| mitochondrial | CYP301 | 2 | 2 | 0 |
| mitochondrial | CYP302 | 2 | 2 | 0 |
| mitochondrial | CYP314 | 1 | 1 | 0 |
| mitochondrial | CYP315 | 1 | 1 | 0 |
| mitochondrial | CYP3118 | 7 | 5 | 2 |
|  | **Totals** | **112** | **89** | **23** |


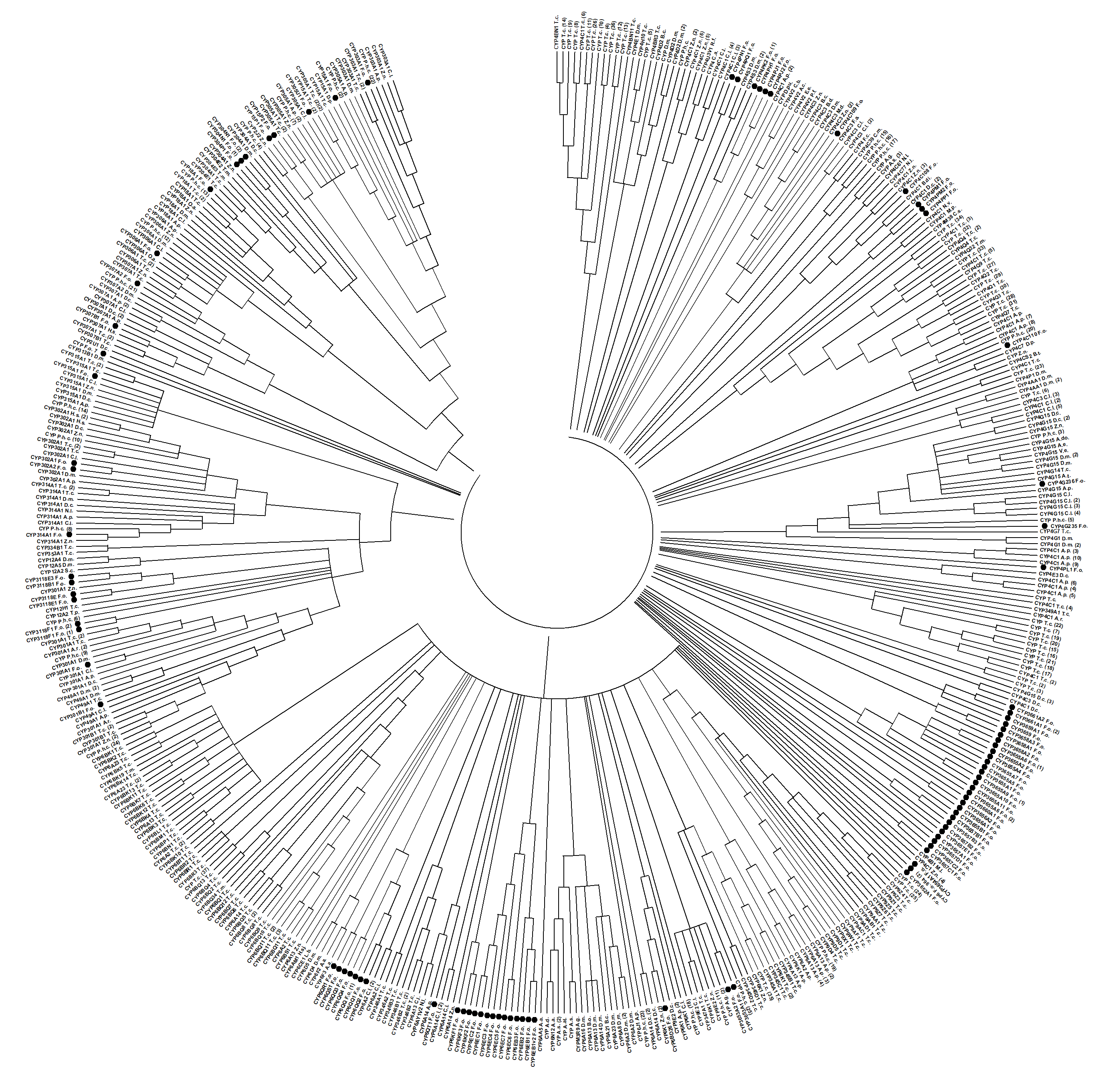


**Clan 3**

**Clan 2**

**mito**

**Clan 4**

**Figure S6.1** Amino acid phylogenetic tree showing *F. occidentalis* P450 genes alongside the most similar P450 sequences from other insect species found in GenBank ( <https://www.ncbi.nlm.nih.gov/genbank/>) as of 8-25-16. Black circles indicate the sequences from *F. occidentalis*. The evolutionary history was inferred using the Neighbor-Joining method (Saitou and Nei, 1987). The bootstrap consensus tree inferred from 1000 replicates is taken to represent the evolutionary history of the taxa analyzed (Felsenstein, 1985). Branches corresponding to partitions reproduced in less than 50% bootstrap replicates are collapsed (Felsenstein, 1985). The evolutionary distances were computed using the Poisson correction method (Zuckerkandl and Pauling, 1965) and are in the units of the number of amino acid substitutions per site. The rate variation among sites was modeled with a gamma distribution (shape parameter = 5). The analysis involved 485 amino acid sequences All ambiguous positions were removed for each sequence pair. There were a total of 1421 positions in the final dataset. Evolutionary analyses were conducted in MEGA X (Kumar et al., 2018). The FOCC OGS v1.0 gene models and other insect species P450 GenBank accessions included in this tree are listed in **Additional file 2: Table S14**.

Cytochrome P450s in development

A steroid hormone, 20-hydroxyecdysone (20E), and juvenile hormone (JH) are known to play essential roles in the insect growth and development process, and the biosynthesis pathway for 20E includes several conserved P450s (Iga and Kataoka, 2012). The P450 genes responsible for the synthesis of 20E include CYP307A1/A2, CYP306A1, CYP302A, CYP315A1, and CYP314A1. Given the importance of 20E in development, knockouts of these genes in *D. melanogaster* has been shown to produce striking phenotypes and has led these P450s to be called “spook/spookier”, “phantom”, “disembodied”, “shadow”, and “shade”, respectively. In addition, CYP18A1 is known to be a key enzyme involved in the inactivation of 20E and is essential for metamorphosis in *D. melanogaster* (Guittard et al., 2011). In the *F. occidentalis* genome, corresponding homologs for each of these genes were identified (**Table S6.2**). Not unexpectedly, these evolutionarily conserved P450 genes showed some of the highest amino acid conservations observed among the P450s from the *F. occidentalis* genome versus P450s in the genomes of other sequenced insects; nonetheless, these identities were modest, ranging from 33-71%.

**Table S6.2.** P450 genes known to be involved in 20E biosynthesis or inactivation, their location within the *F. occidentalis* genome and their identity at the amino acid level versus orthologs in the genomes of other insect species.

| P450 gene (Dm name)^z^ | Locations of *F. occidentalis*  P450 20E pathway orthologs | Amino acid identity versus other insect species^zy^ | | | | | | | |
| --- | --- | --- | --- | --- | --- | --- | --- | --- | --- |
|  |  | *Phc* | *Ap* | *Cl* | *Dc* | *Zn* | *Tc* | *Dm* |  |
| CYP302a1 (Disembodied) | Scaffold54:583491-586227 | 45% | 45% | 41% | 45% | 47% | 42% | 45% |  |
|  | Scaffold117:731027-733573 | 45% | 43% | 41% | 45% | 46% | 45% | 43% |  |
| CYP306a1 (Phantom) | Scaffold261:103212-126374 | 47% | 36% | 46% | 33% | 50% | 45% | 44% |  |
| CYP307a1/a2 (Spook/Spookier) | Scaffold2:2913680-2929983 | 59% | 51% | 45% | 52% | 60% | 61% | 49% |  |
|  | Scaffold144:498693-502969 | 44% | 50% | 54% | 46% | 41% | 52% | 36% |  |
| CYP314a1 (Shade) | Scaffold820:12383-33648 | 63% | 58% | 59% | 58% | 67% | 44% | 48% |  |
| CYP315a1 (Shadow) | Scaffold156:439070-441178 | 42% | 35% | 38% | 36% | 49% | 44% | 37% |  |
| CYP18a1 | Scaffold261:97159-101980 | 71% | 55% | 59% | 58% | 69% | 66% | 60% |  |

^z^Organism used for comparisons are as follows: *Dm* = *D. melanogaster*, *Tc* = *T. castaneum*, *Phc* = *Pediculus humanus corporis*, *Ap* = *A. pisum*, *Dc* = *D. citri*, *Cl* = *C. lectularius*, *Zn* = *Z. nevadensis*

^y^The full *F. occidentalis* P450 protein sequence was compared to the most similar P450 protein sequence from each respective genome.

In addition to having important roles in the 20E pathway, CYPs are also known to be involved in other aspects of development. CYP301A1 is conserved within the genome of other insects and has been shown to play a key role in the formation of adult cuticle in *D. melanogaster* (Sztal et al., 2012). In *F. occidentalis*, two P450s (Scaffold74:870617-877535 and Scaffold74:876952-880140) (**Additional file 2: Table S13**) corresponding to CYP301A1 showed relatively high similarity (47-68%) to CYP301A1 orthologs in other insect genomes. It would be suspected that these CYPs may also be involved in thrips development in a similar manner to that observed in other insect species.

## 6.2 ATP binding cassette and Carboxylesterase genes

*Contributed by Wannes Dermauw, Simon Snoeck, and Thomas Van Leeuwen*

## 6.2.1 Gene set manual annotations – customized strategy and phylogenetic analysis

ATP-binding Cassette Transporter (ABC) gene family

The ABC genes of *F. occidentalis* were identified as previously described (Dermauw, Wannes et al., 2013, Sturm et al., 2009). Briefly, highly conserved nucleotide binding domains (NBD) of *D. melanogaster* ABC proteins were used as queries in tBLASTn searches (E-value threshold <E^-5^) against the genome sequence assembly of *F. occidentalis* (Altschul et al., 1990). *F. occidentalis* ABC gene models were refined or created based on homology and available RNA-seq data. Full-length ABC genes and those genes with a sequence length larger than 70% of the average sequence length of full-length ABC genes per ABC subfamily, were considered as putative ABC genes. All *F. occidentalis* ABC gene sequences that have been annotated in this study can be found in **Additional file 2: Table S15**. Assignment of *F. occidentalis* ABC proteins to the different ABC subfamilies (A-H) was assessed by a BLASTp search against *D. melanogaster* ABC protein sequences and the NCBI protein database (Altschul et al., 1990). In addition, we also performed a phylogenetic analysis of the nuclear binding domains (NBDs) of all annotated *F. occidentalis* ABC genes (encoded by either putative or incomplete/pseudo ABC genes). NBDs were extracted from the ABC protein sequences of *F. occidentalis* using the ScanProsite facility (de Castro et al., 2006) and the Prosite profile PS50893. The alignment of the NBDs of *F. occidentalis*, *Drosophila melanogaster*, *Daphnia pulex* and *Homo sapiens* was performed with MAFFT v7 with 1000 iterations and the options “E-INS-I” and “reorder” (Katoh et al., 2002). A phylogenetic analysis was performed on the Cipres web portal using RAxML v8 HPC2-XSEDE (Stamatakis, 2006) with the automatic protein model assignment algorithm using maximum likelihood criterion and 250 bootstrap replicates. The LG likelihood with empirical base frequencies was chosen as the best scoring model by RAxML. We also performed a phylogenetic analysis for the ABCA, ABCC, ABCG and ABCH subfamilies, using the same methodology as described above except that complete ABC protein sequences were used instead of NBDs and more arthropod species were included (*Anopheles gambiae, Apis mellifera*, *Bemisia tabaci, Bombyx mori, Lygus hesperus, Laodelphax striatellus, Plutella xylostella, Tribolium castaneum* and *Tetranychus urticae*) (Dermauw, W. and Van Leeuwen, 2014, Hull et al., 2014, Qi et al., 2016, Sun, H. et al., 2017, Tian et al., 2017). In addition, only *F. occidentalis* ABC proteins encoded by putative ABC genes (see above for definition) were included, except for FoABCC-12, FoABCG-18 and FoABCH-06 (<70% average length), being highly conserved in other insects. In all four analyses, The LG model with empirical base frequencies was chosen as the best scoring model by RAxML. All phylogenetic trees were visualized, optimized and mid-point rooted with MEGA6 (Tamura et al., 2013) and edited in Corel-DRAW Home & Student x7.

Carboxyl/cholinesterase (CCE) gene family

*D. melanogaster* and *Acyrthosiphon pisum* CCE protein sequences were used as queries to perform tBLASTn (E-value threshold <E 10^-5^) searches against the genome sequence assembly of *F. occidentalis.* CCE gene models were refined or created based on the homology and available RNA-seq data. Full length CCE genes and incomplete genes that had a sequence length larger than 70% of the average sequence length of complete CCEs were considered as putative CCEs and were included in a phylogenetic analysis. All *F. occidentalis* CCE gene models that have been annotated in this study can be found in **Additional file 2: Table S16**. CCE sequences of *F. occidentalis*, *Drosophila melanogaster*, *Apis mellifera*, *Acyrthosiphon pisum* and *B. tabaci* MEAM1 (Chen et al., 2016, Claudianos et al., 2006, Ramsey et al., 2010) were aligned using MAFFT v7 with 1000 iterations and the options “l-INS-i” and “reorder” (Katoh et al., 2002). Only sequences larger than 400 AA were retained for further analysis (~ 70% length average CCE *F. occidentalis*). The Cipres web portal was used to perform the phylogenetic analysis (Miller et al., 2010) using RAxML v8 HPC2-XSEDE (Stamatakis, 2014) with the automatic protein model assignment algorithm using maximum likelihood criterion and 250 bootstrap replicates. The LG likelihood with fixed base frequencies was chosen as the best scoring model by RAxML. The resulting tree was visualized, midpoint rooted and optimized with MEGA6 (Tamura et al., 2013) and edited in Corel-DRAW Home & Student x7.

## 6.2.2. Results and Discussion

ABC gene family

The ABC protein family is one of the largest protein families and present in all kingdoms of life. The majority functions as primary active transporters, hydrolyzing ATP to transport substrates across membranes Some ABC proteins, however, are receptors or are involved in translation. ABC proteins are divided into eight (A to H) groups in Metazoa, of which the ABCB full transporters [also named P-glycoproteins, P-gps, multidrug resistance proteins (MDRs)], ABCCs [also named multi drug resistance associated proteins (MRPs)] and ABCGs have been linked to xenobiotic resistance (Dermauw, W. and Van Leeuwen, 2014). We annotated 45 putative ABC genes in the genome of *F. occidentalis* (**Table S6.3**). This number is similar to those found in *D. melanogaster* (Dermauw, W. and Van Leeuwen, 2014) but less than those identified in other insect genomes, including those of *B. tabaci* and *L. hesperus* (Hull et al., 2014, Tian et al., 2017, Xie et al., 2018), member of the Hemiptera, a sister group of the Thysanoptera (Johnson et al., 2018). For those ABC proteins that are considered as conserved in metazoan species (ABCB half transporters, ABCDs, ABCE, ABCFs, FoABCC-04, FoABCG-11 and FoABCG-11; (Dermauw, W. and Van Leeuwen, 2014); **Table S6.3, Figure S6.2, Figure S6.4 and Figure S6.5**), we found orthologues in *F. occidentalis*. In many cases, we also detected *F. occidentalis* orthologues of ABC proteins that are conserved across most insects (FoABCA-01, FoABCG-01, FoABCG-02, FoABCG-04, FoABCG-05, FoABCG-08, FoABCG-13, FoABCG-21, FoABCH-01, FoABCH-03 and FoABCH-06 (Dermauw, W. and Van Leeuwen, 2014), **Figure S6.3, Figure S6.5, Figure S6.6**). We also found a clear *F. occidentalis* ortholog (FoABCC-12) for the *D. melanogaster* sulfonylurea receptor (sur) within the ABCC subfamily (**Figure S6.4**). In contrast to *T. castaneum* and *T. urticae* we did not identify lineage specific expansions within the ABCC subfamily, a family well known for their role in multidrug resistance (Schinkel and Jonker, 2012). However, similar to *T. urticae*, *D. pulex*, *P. xylostella* and *B. tabaci* (belonging to the Hemiptera, a sister-group of the Thysanoptera) we identified a lineage specific expansion of ABCH genes within the *F. occidentalis* genome (**Figure S6.6**)*.* The physiological functions of lineage-specific ABCH expansions remain, however, largely uncharacterized. Differential expression of ABCH genes has been reported for *T. urticae* females in diapause (Bryon et al., 2013) and some ABCH genes were overexpressed in insecticide/acaricide resistant strains of *T. urticae* (Dermauw, W. et al., 2013) and *P. xylostella* (Qi et al., 2016) suggesting that these lineage-specific ABCHs in *F. occidentalis* might play a role in response to environmental change or exposure to xenobiotic compounds.

**Table S6.3. Gene numbers in ATP-binding cassette (ABC) gene subfamilies of nine arthropod species and *Homo sapiens****

| Species | Order | A | B-FT | B-HT | C | D | E | F | G | H | Total |
| --- | --- | --- | --- | --- | --- | --- | --- | --- | --- | --- | --- |
| *Homo sapiens* | Mammalia: Monotremata | 12 | 4 | 7 | 12 | 4 | 1 | 3 | 5 | 0 | 48 |
| *Daphnia pulex* | Crustacea: Cladocera | 4 | 2 | 5 | 7 | 3 | 1 | 4 | 24 | 15 | 65 |
| *Tetranychus urticae* | Arachnida: Acari: Trombidiformes | 9 | 2 | 2 | 39 | 2 | 1 | 3 | 23 | 22 | 103 |
| *Drosophila melanogaster* | Insecta: Diptera | 10 | 4 | 4 | 14 | 2 | 1 | 3 | 15 | 3 | 56 |
| *Apis mellifera* | Insecta: Hymenoptera | 3 | 3 | 4 | 9 | 2 | 1 | 3 | 15 | 3 | 43 |
| *Tribolium castaneum* | Insecta: Coleoptera | 10 | 2 | 4 | 35 | 2 | 1 | 3 | 13 | 3 | 73 |
| *Plutella xylostella* | Insecta: Lepidoptera | 15 | 7 | 7 | 21 | 3 | 1 | 3 | 19 | 6 | 82 |
| *Bombyx mori* | Insecta: Lepidoptera | 6** | 5 | 4 | 15 | 2 | 1 | 3 | 13 | 3 | 52 |
| *Lygus hesperus**** | Insecta: Hemiptera: Heteroptera | 11 | 3 | 3 | 12 | 2 | 1 | 3 | 19 | 11 | 65 |
| *Bemisia tabaci* Q | Insecta: Hemiptera: Sternorrhyncha | 8 | 0 | 3 | 6 | 2 | 1 | 3 | 23 | 9 | 55 |
| ***Frankliniella occidentalis*** | **Insecta: Thysanoptera** | **3** | **1** | **4** | **11** | **2** | **1** | **3** | **14** | **6** | **45** |

* numbers were derived from (Dermauw, W. and Van Leeuwen, 2014, Hull et al., 2014, Qi et al., 2016, Tian et al., 2017)

** a BLASTp analysis of *B. mori* ABCA proteins against the current *B. mori* annotation (January 2019) in the NCBI database revealed that the old *B. mori* ABCA gene models (BGIBMGA accessions) were incorrect and that some old models should be merged or updated, resulting in only six *B. mori* ABCA genes, instead of nine as previously reported

*** based on transcriptomic data

CCE gene family

The carboxyl/cholinesterase (CCE) enzyme family catalyzes the hydrolysis of carboxylesters and plays role in many biological processes, such as neuron signaling, development and detoxification of xenobiotics, including insecticides (Claudianos et al., 2006, Després et al., 2007, Oakeshott et al., 2005). Within the CCEs 13 clades can be distinguished, which in turn can be grouped into 3 classes: the dietary/ detoxification enzymes (clades A–C), the pheromone/hormone processing enzymes (clades D–G) and the neurodevelopmental CCEs (clades I–M, the majority being non catalytic esterases) (Claudianos et al., 2006, Oakeshott et al., 2005). We annotated 50 putative full-length CCE genes and 16 incomplete/pseudogenes in the *F. occidentalis* genome (**Table S6.4; Additional file 2: Table S16**). This number is similar to what is found in *B. tabaci* MEAM1 (51) and *T. castaneum* (49) but higher than those in *D. melanogaster* (35) and *Acyrthosiphon. pisum* (29) (**Table S6.4**, (Chen et al., 2016, Oakeshott et al., 2010, Ramsey et al., 2010, Yu et al., 2009)).

**Table S6.4. Number of carboxyl/choline esterase (CCE) genes in insect species from different insect orders^*, **^**

| CCE classes and clades | *Dm* | *Bm* | *Am* | *Tc* | *Ap* | *Bt* MEAM1*** | ***Fo*** |
| --- | --- | --- | --- | --- | --- | --- | --- |
| Detoxification/Dietary | 13 | 55 | 8 | 26 | 5 | 7 | **28** |
| Pheromone/hormone processing | 8 | 8 | 5 | 11 | 17***** | 17**** | **7****** |
| Neuro/Developmental (total) | 14 | 13 | 11 | 12 | 7 | 11 | **15** |
| Clade H - Glutactins | 4 | 0 | 0 | 1 | 0 | 1 | **2** |
| Clade J - Acetylcholinesterase | 1 | 2 | 2 | 2 | 2 | 2 | **2** |
| Clade K - Gliotactin | 1 | 1 | 1 | 1 | 1 | 1 | **1** |
| Clade L - Neuroligins | 4 | 6 | 5 | 5 | 3 | 6 | **7** |
| Clade M - Neurotactin | 2 | 2 | 1 | 2 | 0 | 0 | **1** |
| Clade I - Uncharacterized CCEs | 2 | 2 | 2 | 1 | 1 | 2 | **2** |
| Total | 35 | 76 | 24 | 49 | 29 | 36 (51) | **50** |

*numbers were derived from Ramsey et al., 2010; Yu et al., 2009; Oakeshott et al., 2010; Chen et al., 2016 and this study

**abbreviations: *Dm, Drosophila melanogaster*, *Bm, Bombyx mori*, *Am*, *Apis mellifera*, *Tribolium castaneum*, *Acyrthosiphon pisum, Bt, Bemisia tabaci* and *Fo*, *Frankliniella occidentalis*

*** assignment of *B. tabaci* MEAM1 CCEs [having a clear BLASTp hit with arthropod CCEs in the NCBI database and being larger than 400 amino acids (Bta09993/Bta08457 and Bta00353/Bta03441/Bta04865/Bta05217/Bta06364/Bta08029/Bta08783/Bta08786/Bta09351, Bta09992/Bta10442/Bta11683/Bta12234 did not fulfill the first and second criterium, respectively): 36 out of 51 CCEs in Chen et al. 2016] to different classes and/or clades was based on Figure S6.7; the total number of *B. tabaci* CCEs is (51) shown between brackets

**** *F. occidentalis* and *B. tabaci* MEAM1 CCEs that did not cluster into the Detoxification/Dietary class nor the Neuro/Developmental clades in Figure S6.7 were designated as members of the Pheromone/hormone processing class

********* only 13 *Acyrthosiphon pisum* CCEs of the Pheromone/hormone Processing class were larger than 400 AA and were included in the phylogenetic analysis shown in Figure S6.7.

Based on a phylogenetic analysis with CCEs from *D. melanogaster*, *A. mellifera, A. pisum and B. tabaci*, the *F. occidentalis* CCEs could be assigned to the different CCE classes and/or clades (Claudianos et al., 2006, Oakeshott et al., 2005): 28 within the dietary/detoxification class, 7 within the pheromone/hormone processing class and 15 within the neurodevelopmental class. In several cases (mainly within the neurodevelopmental class) clear orthologous relationships were identified between *F. occidentalis* CCEs and those of other insect species (**Figure S6.7**) [FoCCE-28 and FoCCE-57 (clade J, AChE), FoCCE-12 (clade K, gliotactin), FoCCE-04, FoCCE-13, FoCCE-06/FoCCE-15, FoCCE-19/FoCCE-25 and FoCCE-39 (clade L, neuroligins), FoCCE-34 (clade M, neurotactins), FoCCE-03 and FoCCE-35 (clade I)]. Clade J contains CCE genes coding for acetylcholinesterase (AChE), a key enzyme in the central nervous system and in which mutations are known to confer organophosphate and/ or carbamate resistance. Contrary to *D. melanogaster*, most insects have two AChEs and it was hypothesized that the two genes were derived from an old duplication before the split of the Arthropoda (Huchard et al., 2006). In line with hypothesis, two AChEs [FoCCE-57, ortholog of Ace1, and FoCCE-28, ortholog of Ace2] could be identified in the *F. occidentalis* genome. Furthermore, we found three *F. occidentalis* CCEs (FoCCE-16, FoCCE-18, FoCCE-47) that clustered with high bootstrap support with *A. mellifera* GB15327 and GB10820, previously characterized as juvenile hormone esterase-like enzymes (Mackert et al., 2008) (**Figure S6.7**). Finally, in line with other species included in our analysis, we found a lineage-specific expansion of *F. occidentalis* CCEs within the dietary/detoxification enzyme class. Such expansions have also been reported for other species (**Table S6.4, Figure S6.7** (Yu et al., 2009)), but except for the herbivorous *B. mori,* this is the largest expansion of dietary/detoxification CCEs reported for an insect species (Table S6.4). Future work should confirm whether *F. occidentalis* CCEs are indeed detoxification CCEs and whether their expansion might be related to the polyphagous nature and/or fast resistance development of *F. occidentalis* (Jensen, 2000)*.*


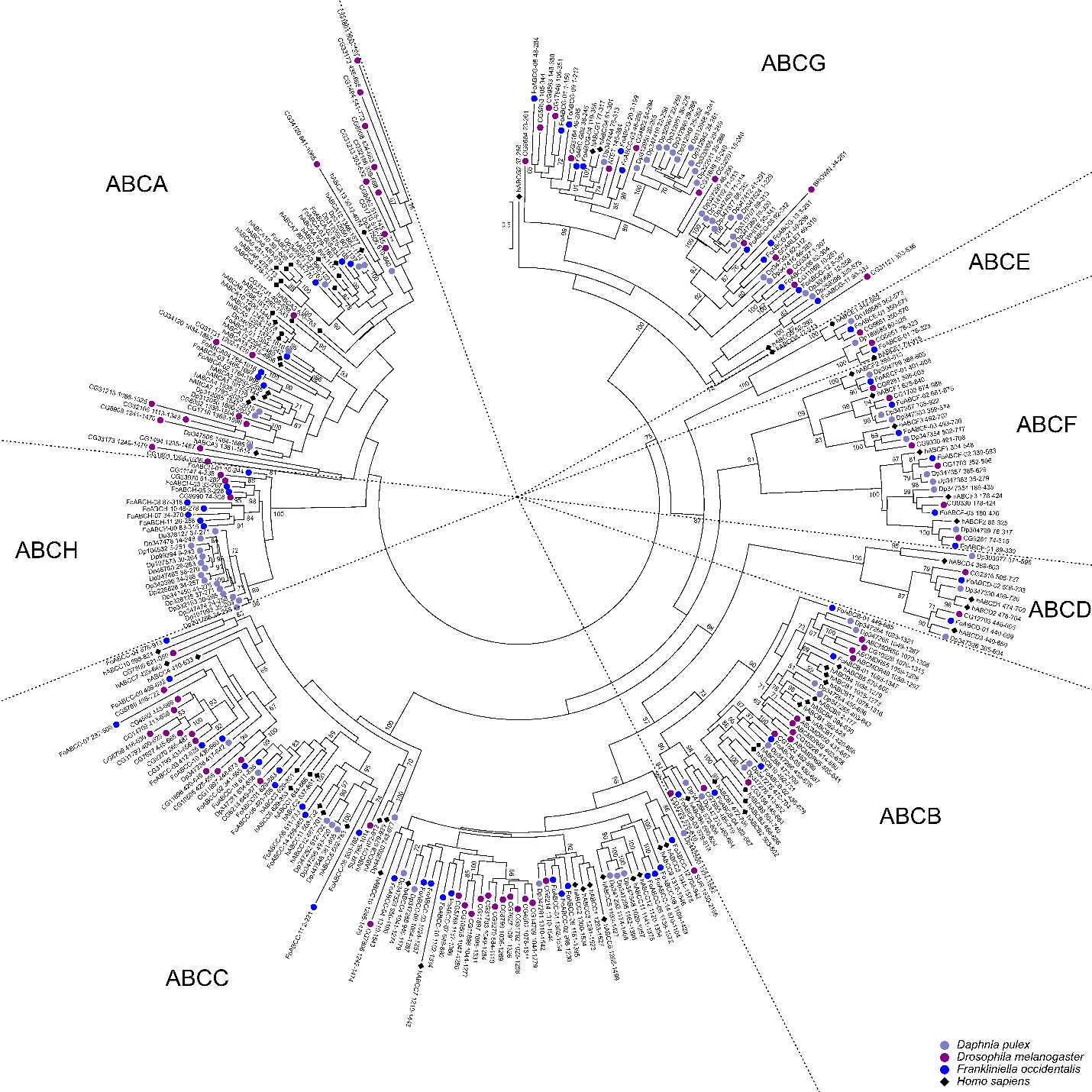


**Figure S6.2 - Maximum likelihood phylogenetic analysis of the NBDs of ABC proteins.** Maximum likelihood phylogenetic analysis of the NBDs of ABC proteins of *Daphnia pulex*, *Drosophila melanogaster*, *Frankliniella occidentalis* and *Homo sapiens.* The scale bar represents 0.5 amino-acid substitutions per site. Numbers behind the accession ID or name of an ABC genes indicate the position of the NBD in the ABC protein sequence. The different metazoan ABC protein subfamilies (A-H) are labelled.


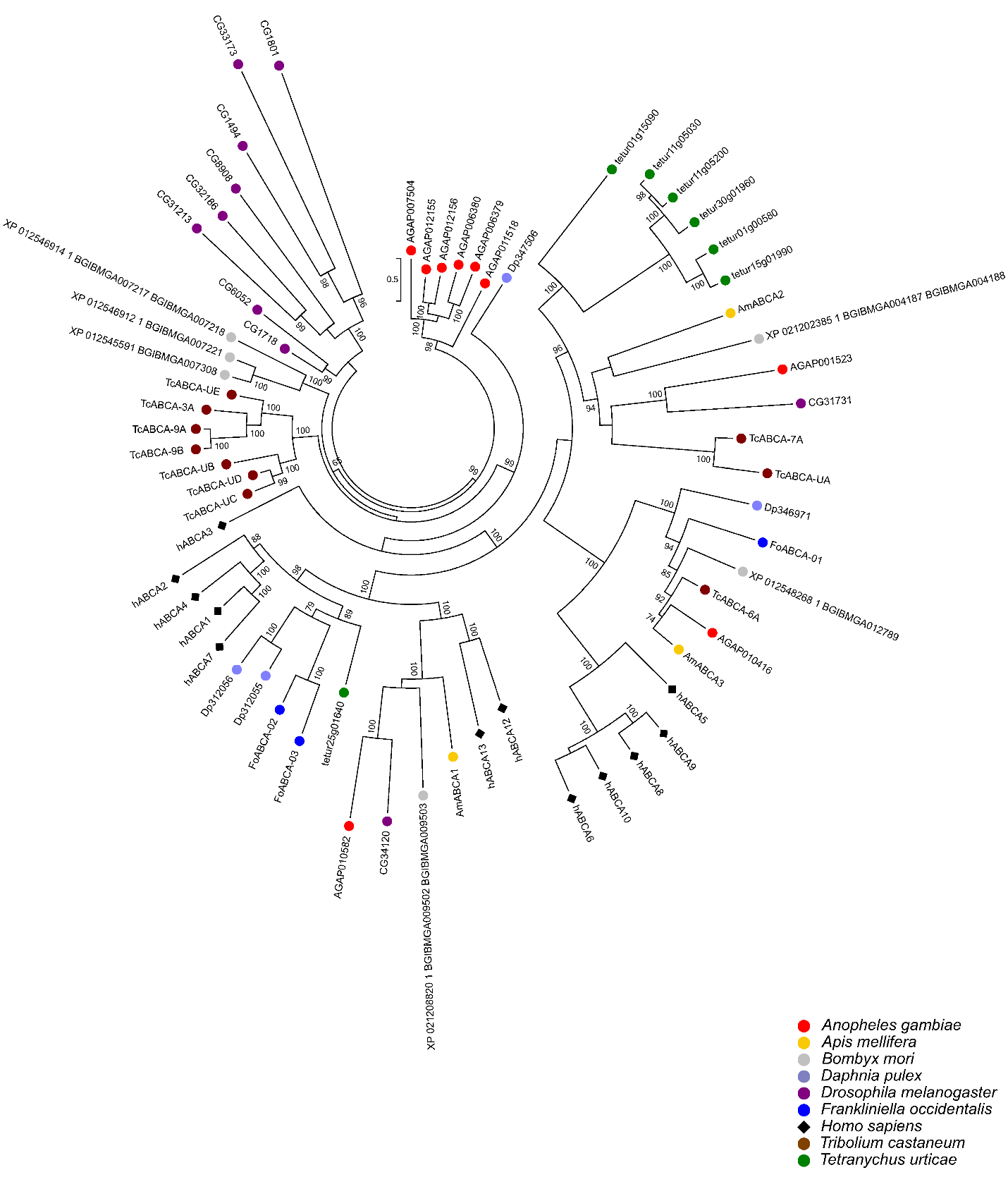


**Figure S6.3** - **Maximum likelihood phylogenetic analysis of arthropod ABCA proteins.** Maximum likelihood phylogenetic analysis of the ABCA proteins of *Anopheles gambiae*, *Apis mellifera*, *Daphnia pulex*, *Drosophila melanogaster*, *Frankliniella occidentalis*, *Homo sapiens*, *Tribolium castaneum* and *Tetranychus urticae.* The scale bar represents 0.5 amino-acid substitutions per site.


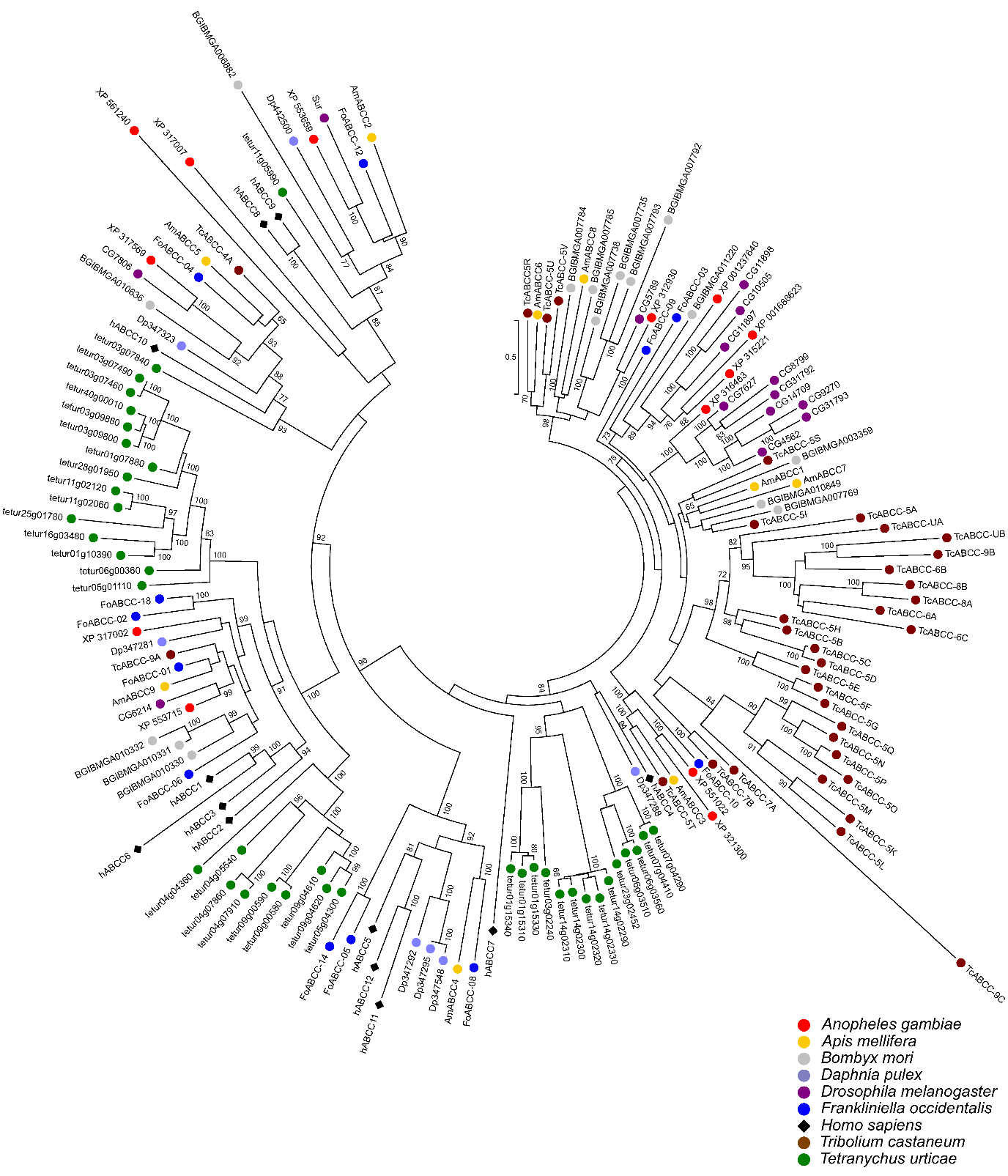


**Figure S6.4** - **Maximum likelihood phylogenetic analysis of arthropod ABCC proteins.** Maximum likelihood phylogenetic analysis of the ABCC proteins of *Anopheles gambiae*, *Apis mellifera*, *Bombyx mori*, *Daphnia pulex*, *Drosophila melanogaster*, *Frankliniella occidentalis*, *Homo sapiens*, *Tribolium castaneum* and *Tetranychus urticae.* The scale bar represents 0.5 amino-acid substitutions per site.


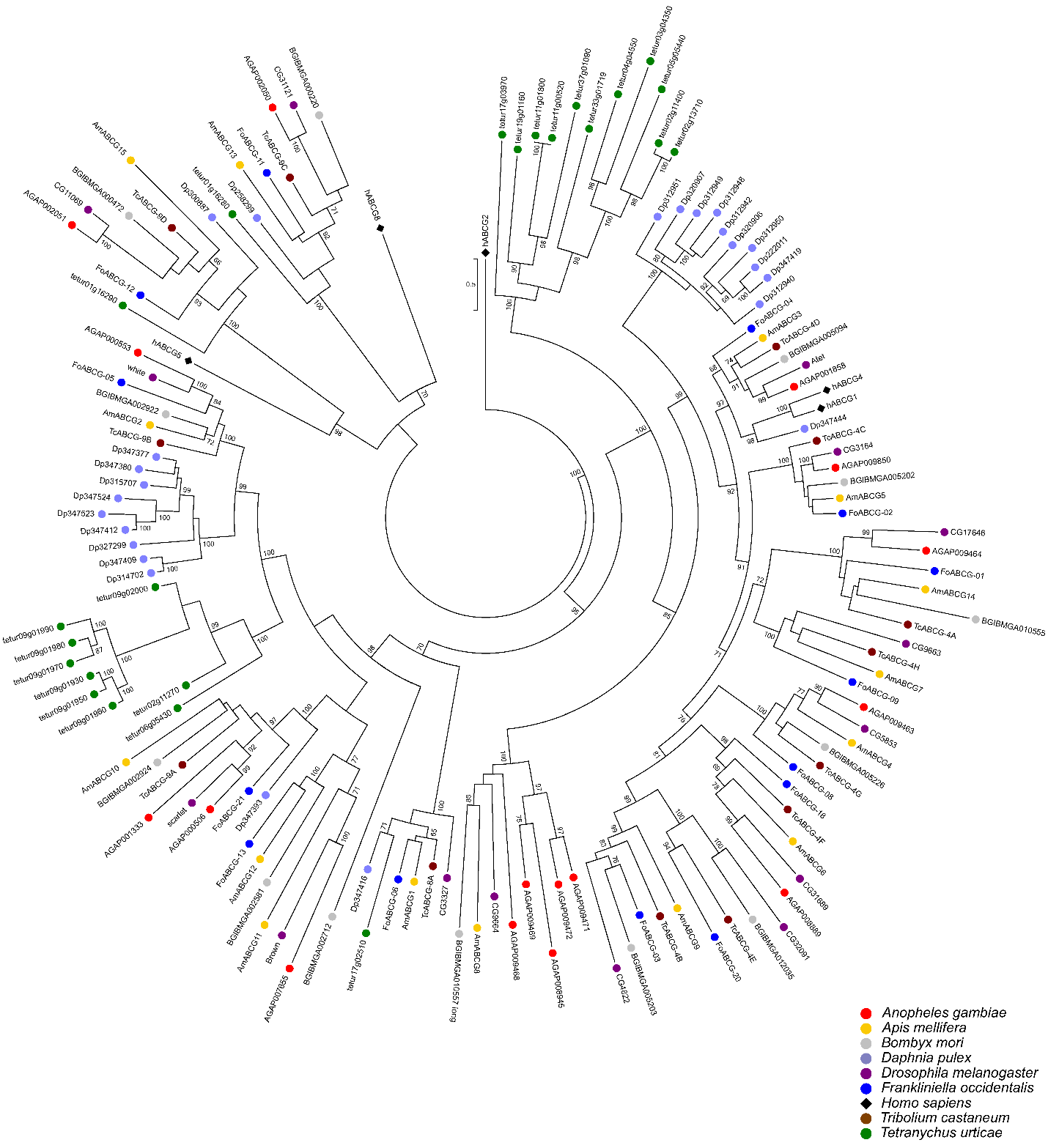


**Figure S6.5** - **Maximum likelihood phylogenetic analysis of arthropod ABCG proteins.** Maximum likelihood phylogenetic analysis of the ABCG proteins of *Anopheles gambiae*, *Apis mellifera*, *Bombyx mori*, *Daphnia pulex*, *Drosophila melanogaster*, *Frankliniella occidentalis*, *Homo sapiens*, *Tribolium castaneum* and *Tetranychus urticae.* The scale bar represents 0.5 amino-acid substitutions per site.


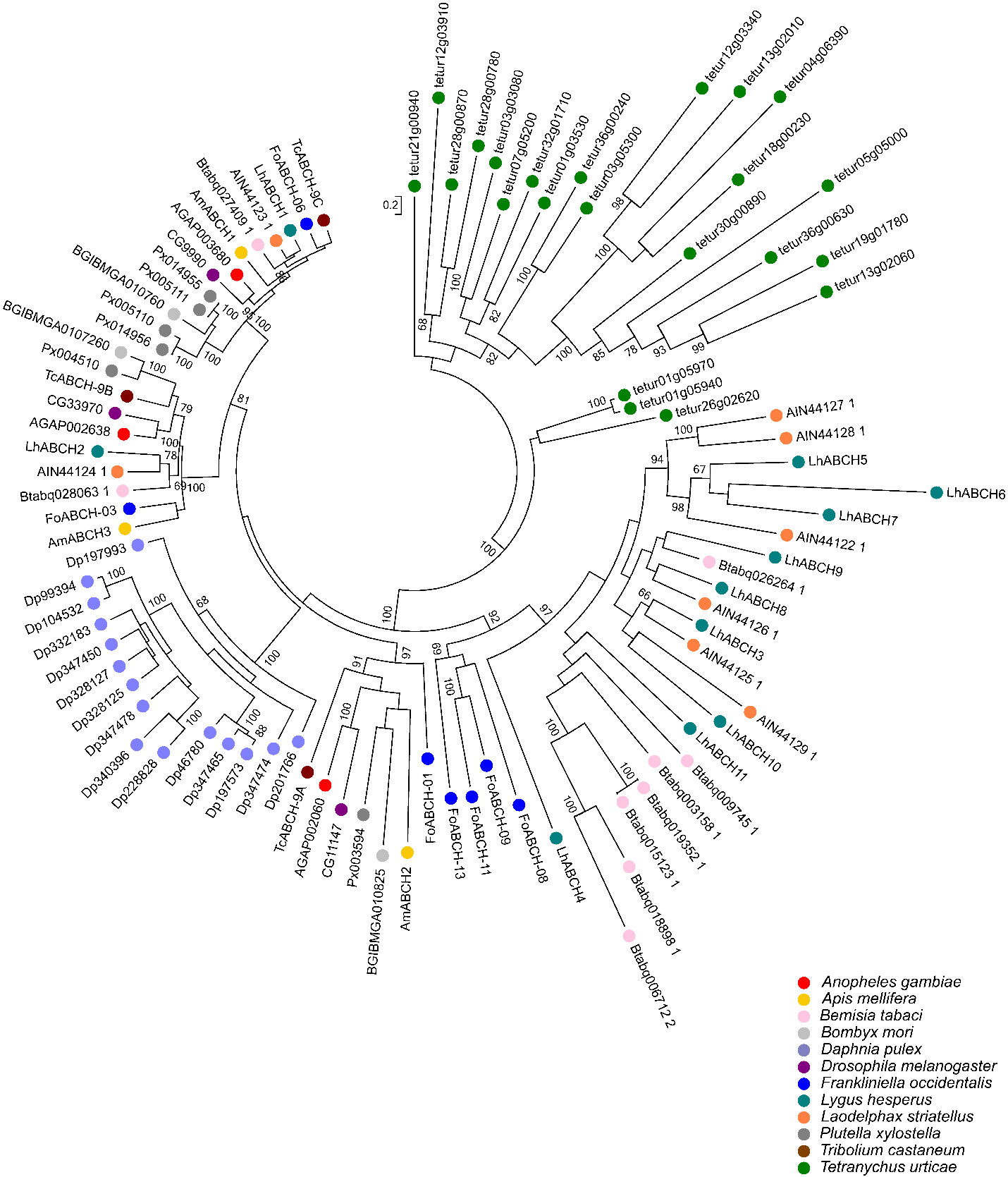


**Figure S6.6** - **Maximum likelihood phylogenetic analysis of arthropod ABCH proteins.** Maximum likelihood phylogenetic analysis of the ABCH proteins of *Anopheles gambiae*, *Apis mellifera*, *Bemisia tabaci*, *Bombyx mori*, *Daphnia pulex*, *Drosophila melanogaster*, *Frankliniella occidentalis*, *Lygus hesperus*, *Laodelphax striatellus*, *Plutella xylostella*, *Tribolium castaneum* and *Tetranychus urticae.* The scale bar represents 0.2 amino-acid substitutions per site.


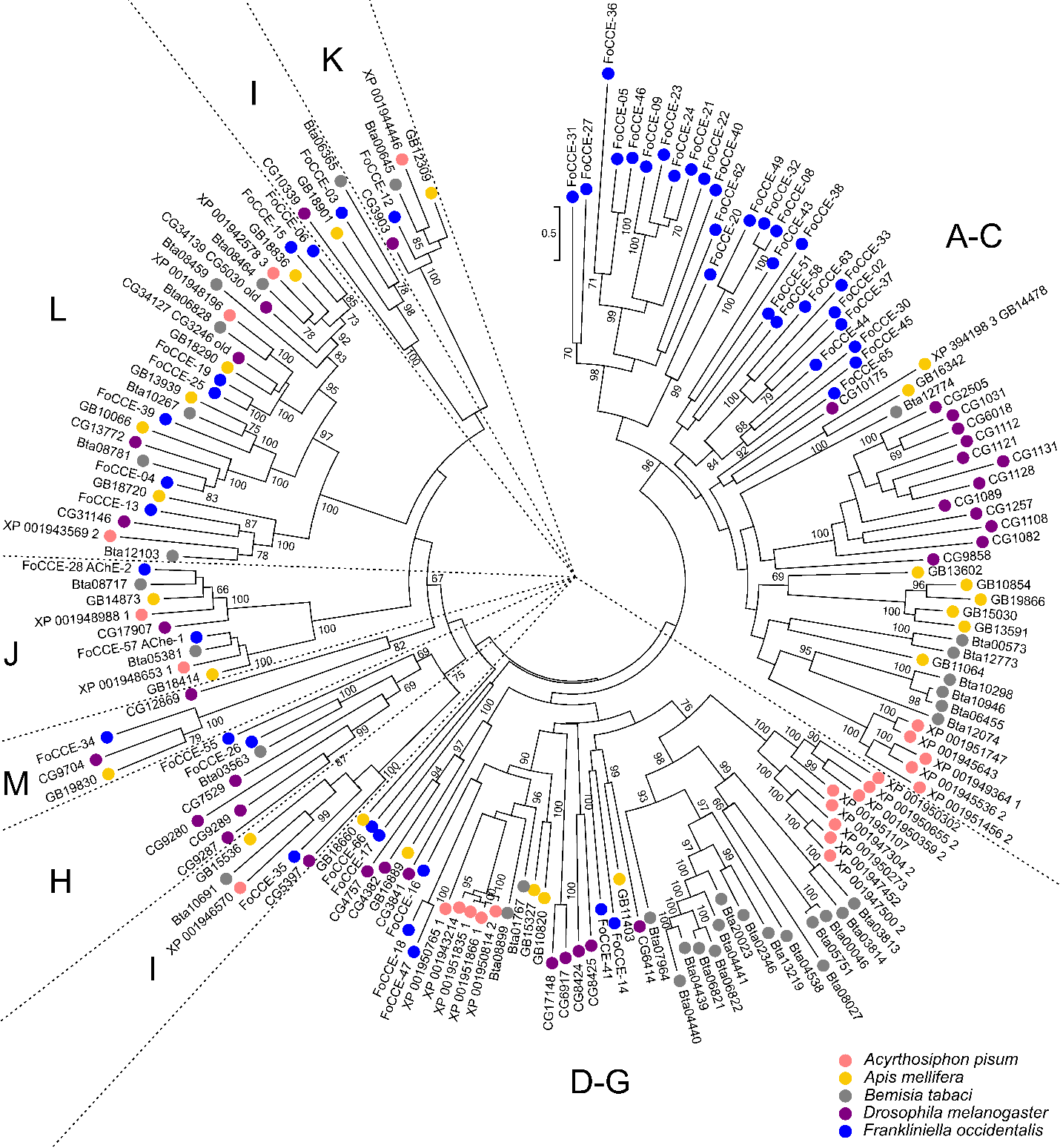


**Figure S6.7** - **Maximum likelihood phylogenetic analysis of arthropod CCEs.** Maximum likelihood phylogenetic analysis of the CCEs of *Acyrthosiphon pisum*, *Apis mellifera*, *Bemisia tabaci*, *Drosophila melanogaster* and *Frankliniella occidentalis.* The CCEs clustered into classes and/or clades (Claudianos et al., 2006): A-C (dietary class), D-G (hormone/semiochemical class), H (glutactin and like enzymes), I (uncharacterized CCEs), J (AChEs), K (gliotactins), L (neuroligins), M (neurotactins). The scale bar represents 0.5 amino-acid substitutions per site.

# 7. Innate Immune genes

*Contributed by Chris G. C. Jacobs, Maurijn Vander Zea, Jonathan Oliver and Swapna Priya Rajarapu*

**7.1. Abstract**

The *Frankliniella. occidentalis* genome encodes canonical immune gene family members, and we discovered apparent gains in pathogen recognition receptors (PGRP and GNBPs) and members of the melanization pathway, in particular prophenoloxidases (PPO). However, there was apparently an absence of two genes in the IMD pathway, a signaling pathway activated in response to gram negative bacteria (Lemaitre and Hoffmann, 2007) and in some reports, viruses (Kingsolver et al., 2013, Mussabekova et al., 2017). Thrips proteins that were shown to bind to the tomato spotted wilt virus (TSWV) proteins and may have anti-viral and/or pro-viral functions were described previously (Badillo-Vargas, I. E. et al., 2019).

**7.2. Background**

Insects, including thrips, encounter a wide variety of bacteria ranging from beneficial to highly pathogenic, along with fungi, viruses and parasites. To combat unwanted microbes, insects rely on innate immune defense mechanisms which have been best studied in *Drosophila* (Lemaitre and Hoffmann, 2007, Ligoxygakis, 2013). The most prominent response is the humoral or systemic response which involves the secretion of massive amounts of antimicrobial peptides (AMPs) by the fat body into the haemolymph (Ganesan et al., 2011). Local immune responses by epithelia include the production of AMPs and the generation of Reactive Oxygen Species (ROS) by the enzyme Duox (Dual Oxidase) (Davis and Engström, 2012, Ferrandon, 2013). Finally, haemocytes execute the cellular response including encapsulation of larger invaders such as parasites, local melanization reactions using phenoloxidase enzymes, and phagocytosis using Scavenger receptors, receptors of the Nimrod family, Dscam, and ThioEster containing Proteins (TEPs) (Vlisidou and Wood, 2015).

Invading microbes are recognized by PeptidoGlycan Recognition Proteins (PGRPs) and Gram Negative Binding Proteins (GNBPs) that activate the two main immune signaling pathways, the Toll and the IMD pathway (Ferrandon, 2013, Ganesan et al., 2011, Lemaitre and Hoffmann, 2007, Ligoxygakis, 2013). Fungi and Gram positive bacteria activate Toll signaling, whereas Gram negative bacteria activate IMD signaling. Intracellular signaling eventually leads to the nuclear localization of the NF-κBs Relish (for the IMD signaling pathway) and Dorsal/Dif (for the Toll signaling pathway), upregulating the transcription of effector genes. Although these two pathways are also activated upon viral infection (Kingsolver et al., 2013, Mussabekova et al., 2017), the main antiviral defense depends on RNAi mechanisms (see section 7.5). In this section we report on the *F. occidentalis* signal transduction, microbe recognition and immune effector genes, and report transcriptome-wide comparisons of innate immune gene expression with two other thrips vector species (*F. fusca* and *Thrips palmi*) to gain insight into species-specific and commonly-expressed innate immune genes.

**7.3. Innate immunity-associated genes**

We have annotated 96 immune genes (**Additional file 2: Table S17**). Gene content of various family members of innate immune genes indicates a well-developed immune competence, particularly concerning the recognition molecules. We found 14 PGRPs (versus none in *A. pisum*, 2 in *Oncopeltus*, 6 in *Tribolium* and 13 in *Drosophila*), and 8 GNBPs (vs 2 in *A. pisum*, 1 in *Oncopeltus*, 3 in *Tribolium* and 3 in *Drosophila*) (Gerardo et al., 2010, Panfilio et al., 2019). Perhaps gains in these pathogen recognition genes indicates adaptation to a diverse microbiome/viromes residing in/on the wide plant host range of this thrips species. Concerning the effector genes, the melanization pathway seems notably extensive. We found 6 prophenoloxidases vs. 2 in *A. pisum* and 3 in *Oncopeltus*, *Tribolium* and *Drosophila* (Gerardo et al., 2010, Panfilio et al., 2019). finding is the absence of the signal transducing molecule IMD itself. Absence of IMD has also been reported for the Hemipteran species *Bemisia tabaci* and *Diaphorina citri* (Arp et al., 2016, Chen et al., 2016, Gerardo et al., 2010, The International Aphid, Genomics Consortium, 2010, Zhang et al., 2014). In *Oncopeltus*, IMD could not be identified by homology searches, but was found by classical cloning with degenerate primers (Panfilio et al., 2019). IMD was also reported missing from the bedbug *Cimex lectularius* (Benoit et al., 2016), but was later found using *Plautia stali* IMD as query (Nishide et al., 2019). This illustrates that IMD sequences can be highly divergent and conclusions about their absence should be drawn with care. However, we could not find *Frankliniella* IMD using any of the available Hemipteran IMD sequences as query (Table S7.2).

For *A. pisum*, it has been suggested that its relation to Gram negative endosymbionts and its rather ‘sterile’ diet of phloem sap account for a generally reduced immune repertoire and the absence of IMD (Altincicek et al., 2008, Gerardo et al., 2010, The International Aphid, Genomics Consortium, 2010). This does not seem valid for the mesophyll feeding thrips species. In contrast to *A. pisum*, almost all other components of the IMD signaling pathway are present in *Frankliniella*, including two Relish molecules (**Additional file 2: Table S17**) known to transcriptionally-regulate the expression of effectors (execution of defense).

**7.4. Comparison of innate immunity transcripts of *F. occidentalis*, *F. fusca* and *T. palmi***

The occurrence and number of innate immunity-associated transcripts varied across the three species (**Table S7.1; and Additional file 8**). Among the pathogen recognition molecules, fibrinogen related proteins were found only in *F. occidentalis* transcriptome. Proteins spaetzle and tube encoding transcripts were not found in *T. palmi* and the primary response protein, myeloid- differentiation and TNF receptor associated-protein 2, important players in Toll pathway, were not found in *F. fusca* transcriptome. Interestingly, Fas-associated Death Domain (FADD), immunodeficiency (IMD) and TGF-β-activated kinase 1 (TAK1) proteins of IMD pathway were absent in all the three species. Components of JAK/STAT were found in all the three species with eight and four transcripts of cytokine receptors found in *F. occidentalis* and *F. fusca*. A single antimicrobial peptide, defensin, was found only in *F. occidentalis* and *F. fusca*. Peroxidasins, autophagy related protein and inhibitors of apoptosis have been well represented among the three transcriptomes.

Six frame translation of *F. occidentalis*, *F. fusca* and *T. palmi* resulted in 994, 296, 737 predicted protein sequences with more than 100 amino acids in length respectively. Among these, 97.38 % of *F. occidentalis*, 99.32% *F. fusca* and 89.96% *T. palmi* were retained due to the presence of predicted coding regions. Further, the start position of these translated proteins was redefined based on the blastp alignment to the homologous sequences in UniProt using a position weighted matrix approach and thus refining 49, 16 and 8 start positions for *F. occidentalis*, *F. fusca* and *T. palmi* respectively. Together with coding sequence prediction and start position refinement, 296 proteins were predicted for *F. occidentalis* transcriptome, 184 proteins were predicted for *F. fusca* transcriptome and 240 proteins were predicted from *T. palmi* transcriptome. Homology based transcript prediction resulted in approximately one predicted protein per transcript.

The canonical genes participating in humoral and cellular immune responses of insects were all identified in the three species except for few members of Toll and IMD signaling pathway (**Table S7.1**). Absence of transcripts encoding core IMD pathway genes was reported for hemipteran vectors, such as *Diaphorina citri* (Asian citrus psyllid) and *Acyrthosiphon pisum* (pea aphid) (Arp et al., 2016, Gerardo et al., 2010). IMD pathway is triggered in defense to gram negative bacteria and viruses. These core genes may be entirely missing or may be highly divergent from other species. A blastx alignments using a lower e-value cut-off (10^-3^) with other recently determined divergent IMD or IMD-like partial sequences from *Cimex lecticularis* (XP_014246002) and *Oncopeltus fasciatus* (Panfilio et al., 2019), respectively, revealed weak alignments ranging in lengths of 20-70 bp (**Table S7.2**). In addition to IMD, absence of transcripts encoding FADD suggests either absence of this downstream process or evolution of an atypical signaling pathway which is yet to be deciphered.

All components of JAK/STAT pathway were identified in all the three thrips species (**Table S7.1; and Additional file 8**). However, transcripts homologous to cytokine receptor were over-represented in *F. occidentalis* and *F. fusca* with eight and four cytokine receptor transcripts respectively. Multiple sequence alignment of these transcripts showed they are not identical indicating that these are different transcripts. Cytokine receptors are involved in many other signaling pathways and multiple transcripts encoding these receptors in *F. occidentalis* and *F. fusca* could be involved in other biological functions including innate immunity.

**Table S7.1.** Tabulation of transcript sequences of three thrips vector species classified into three major roles in innate immunity - recognition, signaling and execution of defense

| **Immune components** | Number of Transcripts | | |
| --- | --- | --- | --- |
| ***Pathogen recognition molecules*** | *F. occidentalis* | *F. fusca* | *T. palmi* |
| C-type lectins | 7 | 3 | 6 |
| Fibrinogen Related proteins | 2 | 0 | 0 |
| Peptidoglycan recognition proteins | 7 | 5 | 1 |
| 1,3-β-D-glucan-binding protein | 2 | 1 | 3 |
| ***Signaling cascades*** |  |  |  |
| *Toll Pathway* |  |  |  |
| Toll receptors | 2 | 2 | 2 |
| Spaetzle | 3 | 2 | 0 |
| Tube | 1 | 1 | 0 |
| Pelle | 1 | 1 | 1 |
| MyD88 | 1 | 0 | 1 |
| CACT | 1 | 1 | 1 |
| TRAF6 | 2 | 0 | 0 |
| *IMD pathway* |  |  |  |
| CASPAR | 1 | 1 | 1 |
| FADD | 0 | 0 | 0 |
| IKKB-ird5 | 2 | 2 | 2 |
| IMD | 0 | 0 | 0 |
| TAK1 | 1 | 1 | 1 |
| TAB | 2 | 0 | 0 |
| *JAK/STAT* |  |  |  |
| DOME | 8 | 4 | 1 |
| HOP | 1 | 1 | 1 |
| STAT | 3 | 1 | 2 |
| ***Response*** |  |  |  |
| Hexamerin | 1 | 1 | 0 |
| Antimicrobial Peptides | 1 | 1 | 0 |
| Lysozymes | 3 | 2 | 3 |
| Peroxidasins | 8 | 4 | 5 |
| Antioxidant enzymes | 9 | 5 | 1 |
| Clip-domain serine proteases | 6 | 3 | 2 |
| Autophagy | 9 | 6 | 6 |
| Prophenoloxidase | 3 | 1 | 3 |
| Inhibitors of Apoptosis | 5 | 5 | 4 |

**Table S7.2:** Blast similarity of *F. occidentalis* transcripts to IMD/IMD-like amino acid sequences^1^ from *Cimex lecticularis* (XP_014246002) and *Oncopeltus fasciatus* (Panfilio et al., 2019).

2019).

| **FOCC transcripts** | **Transcript**  **Length (bp)** | **Subject** | **% identity** | **Alignment Length (aa)** | **E-value** | **bitscore** | **FOCC transcript top match to NCBI nr database (blastx)** |  |
| --- | --- | --- | --- | --- | --- | --- | --- | --- |
| CUFF.473.2 | 8131 | *C. lecticularis* | 31.0 | 58 | 5.46E-04 | 30.0 | hypothetical protein  mediator of rna polymerase ii transcription subunit 14-like |  |
| CUFF.5189.3 | 9814 | *C. lecticularis* | 38.6 | 44 | 5.56E-04 | 30.4 | integrator complex subunit 10 isoform x1 |  |
| FOCC008130-RA | 1287 | *C. lecticularis* | 22.2 | 45 | 7.85E-04 | 26.9 | hypothetical protein |  |
| CUFF.3730.2 | 2801 | *O. fasciatus* | 36.2 | 58 | 3.27E-04 | 29.3 | uncharacterized protein |  |
| FOCC011660-RA | 2404 | *O. fasciatus* | 36.2 | 58 | 2.76E-04 | 29.3 | uncharacterized protein |  |
| FOCC003613-RA | 4359 | *O. fasciatus* | 30.0 | 70 | 6.92E-04 | 28.9 | hypothetical protein/staphylococcal nuclease domain-containing protein |  |
| CUFF.2047.2 | 12389 | *O. fasciatus* | 30.0 | 70 | 0.002 | 28.9 | hypothetical protein/staphylococcal nuclease domain-containing protein |  |
| CUFF.3730.1 | 2719 | *O. fasciatus* | 36.2 | 58 | 3.96E-04 | 28.9 | uncharacterized protein |  |

^1^Due to the absence of IMD/IMD-like sequence matches to publicly available IMD proteins representing diverse members of Arthropoda, these two hemipteroids (more closely related to thrips) were selected for blastx analysis because they were recently discovered and determined to be highly divergent from other insect species.

## 7.5 RNAi pathway genes

*Contributed by Olivier Christiaens, Clauvis N.T. Taning, Guy Smagghe*

RNA interference (RNAi) is a post-transcriptional gene silencing mechanism that is present in most eukaryotic organisms. In insects, three distinct RNAi pathways have been identified, namely the small interfering RNA (siRNA), the micro RNA (miRNA) and piwi-interacting RNA (piRNA) pathways (Dowling et al., 2016). Through these pathways, RNAi is involved in gene expression regulation, protection of the genome against transposons and it is also an important element of the antiviral defense system. The siRNA and miRNA pathways work in a similar way. In the siRNA pathway, longer double-stranded RNA (dsRNA), which can either be of endogenous or exogenous (eg. viral dsRNA) origin is processed by an RNase III enzyme called Dicer-2 into smaller siRNA pieces, which are typically around 21-23bp long. These siRNAs are then taken up in the RNA induced silencing complex (RISC), which is a protein complex also containing an Argonaute enzyme (Ago-2). One of the two strands of the siRNA will be removed from the complex and the remaining single-stranded small RNA will guide the RISC complex to its complementary mRNA and bind to it through base pair binding. This will eventually result in cleaving the mRNA by Ago2, preventing further translation to protein. In the miRNA pathway, pri-miRNA is the initial precursor of the functional miRNA. In the nucleus of the cell, this stem-loop RNA structure, typically a few hundreds of nucleotides long, is processed by the enzyme Drosha into one or more pre-miRNA molecules, also containing a hairpin structure. After transport into the cytoplasm, this pre-miRNA is processed into functional miRNAs by Dicer-1. After this step, the pathway follows a similar process as the siRNA pathway, where the mRNA is eventually destroyed by Ago-1. In insects, both the siRNA and miRNA typically have a separate set of Dicer and Argonaute enzymes, in contrast to for example nematodes which only have one Dicer enzyme. The piRNA pathway finally is an entirely different process, which is still not fully understood. Piwi-interacting RNAs form the largest class of non-coding RNAs in animal cells and are involved in silencing of transposons, epigenetic methylation and play a role in the regulation of genetic elements in germ line cells (Ozata et al., 2019). Similar to the other RNAi pathways, different piwi-interacting proteins, including a Piwi-Argonaute (Ago-3) and Aubergine, guide the piRNAs to their target sequence, for example a transposon, leading to its destruction.

Through several pathways, RNAi is involved in gene expression regulation, protection of the genome against transposons and it is also an important element of the antiviral defense system. The RNAi-related genes constitutes a group of genes that are all members of a diverse range of gene (super)families which are not evolutionarily related, but are linked based on their involvement in RNAi (Christiaens et al., 2014, Swevers et al., 2013). This group includes core machinery genes for the siRNA and miRNA pathways, such as the *dicer* and *argonaute* genes, as well as several genes involved in antiviral immune response and several genes encoding auxiliary proteins. We used methodologies and data as described in Swevers et al. (Swevers et al., 2013), to manually annotate the *F. occidentalis* RNAi genes (Figures S7.1-7.8). *Frankliniella occidentalis* is a known vector for several economically important plant viruses and is considered the primary vector for tospoviruses, including tomato spotted wilt virus (TSWV). These viruses are of great economic importance since they can cause substantial damage to a very large range of food and ornamental plant species. Knowledge of the RNAi machinery in thrips is important because TSWV and other tospoviruses replicate in thrips vectors and thus RNAi may impact the infection cycle in this insect. RNAi has been documented to be effectively elicited in *F. occidentalis* through delivery of dsRNA by injection (Badillo-Vargas, Ismael E. et al., 2015). Annotation of the RNAi genes in thrips enables future experiments examining their role in virus response in thrips and may be informative in the context of evolutionary analysis of thrips-virus relationships within this insect order.

**Figures S7.1 – S7.8. Phylogenetic trees of *F. occidentalis* RNAi pathway genes. (**See Additional file 1, Table S19 for complete list of curated genes)

1. **Ago 1, 2 and 3**

1. **Ago 1**

1. **Ago 2**

1. **Ago 3**

1. **Dicer 1, 2**

1. **Dicer 1**

1. **Dicer 2**

1. **Loquacious and r2d2** (maximum likelihood analysis)

Loquacious

R2D2

# 8. Embryonic and Post-embryonic genes

## 8.1 *Wnt* Signaling Pathway

*Contributed by Iris Vargas Jentzsch and Kristen A. Panfilio*

### 8.1.1. Abstract

The Wnt pathway is a signal transduction pathway with fundamental regulatory roles in embryonic development in all metazoans. The emergence of several gene families of both Wnt ligands and Frizzled receptors allowed the evolution of complex combinatorial interactions with multiple layers of regulation (Murat et al., 2010). Wnt signaling affects cell migration and segment polarity as well as segment patterning and addition in most arthropods (Oberhofer et al., 2014). Surveying and comparing the gene repertoire of conserved gene families within and between taxonomic groups is the first step towards understanding their function during development and evolution.

Here we curated gene models for the main components of the Wnt signaling pathway, and confirmed their orthology by phylogenetic analysis. We found 9 Wnt ligand subfamilies, three Frizzled transmembrane receptor subfamilies, the co-receptor *arrow*, and the downstream components *armadillo*/*beta*-*catenin*, *dishevelled*, *arrow*, *axin*, and *shaggy*/ *GSK*-3. All of these genes, with the exception of the Frizzled family (three *fz-2* paralogs), were present in single copy in the assembly. Three Wnt genes, *wingless*, *Wnt6* and *Wnt10*, were linked on the same scaffold, reflecting the ancient arrangement of Wnt genes in Metazoa.

The thrips Wnt ligand repertoire is comparable to that of other insects and adds to the observations of reduction in ligand diversity in the lineage leading to insects (compare 9 ligands in thrips with 17 total ligand classes known in other animals). Nevertheless, the proposal of gene losses needs to be done with caution when dealing with draft assemblies from second generation sequencing, which is the case for most recently published genomes.

### 8.1.2. Gene set manual annotations – customized strategy

Protein sequences for *Wnt* ligands as well as receptors and downstream components (*armadillo*/ *beta*-*catenin*, *dishevelled*, *frizzled*, *arrow*, *axin*, *shaggy*/ *GSK*-3) from *Drosophila* *melanogaster*, *Tribolium* *castaneum*, *Acyrthosiphon* *pisum* and *Oncopeltus fasciatus,* were retrieved from NCBI, and used to perform standalone tblastn searches on the *Frankliniella occidentalis* scaffolds with a maximum e-value of 1e^-10^. Hits from all species together were ordered by scaffold and start position, and for each group of overlapping or closely adjacent hits from multiple orthologous queries, the putative gene name was identified by blasting back the hit sequence against GenBank, with a taxonomic restriction to Arthropoda accessions. The query sequences with the best hits (lowest e-value) for each gene were then used to identify the model to be curated, by doing a tblastn search into the *Frankliniella* scaffolds from the Blast instance at the National Agricultural Library (<https://i5k.nal.usda.gov/legacy_blast>). The Blast results were visualized in the Web Apollo instance for *Frankliniella* (https://apollo.nal.usda.gov/fraocc/selectTrack.jsp), where the corresponding automated annotation models were edited. To confirm orthology, we then Blasted the edited *Frankliniella* models back into GenBank. Homology, intron/exon boundary assessments, and protein sequence completeness were identified by manual inspection and correction of protein alignments generated with Clustal Omega (http://www.ebi.ac.uk/Tools/msa/clustalo/).

The numbering (subfamily assignment) for *Wnt and fz* orthologs was assigned based on the corresponding vertebrate homolog (the naming of *Drosophila* orthologs was changed accordingly), based on phylogenetic analyses done at http://www.phylogeny.fr/.

Possible gene loci duplications were identified by performing tblastn searches on the scaffolds using the protein sequences of completed annotation models as queries, and then re-blasting the resulting hit sequences into GenBank for Arthropoda hits.

### 8.1.3. Results and Discussion

A total of 26 models for the main Wnt signaling genes were curated on the *F. occidentalis* assembly (**Table S8.1**). There were starting automatic predictions for all annotated genes, except for *frizzled-2a –part 1*, because this is one isolated exon, and the rest of the model was found on another scaffold. All automated models were very accurate, even in the absence of RNA-seq support. Only the beginning of the genes proved more difficult to predict, as we had to edit the start codon or add additional start exons in most of the cases. Most of the models were present on individual scaffolds, and three models were split into two parts on different scaffolds (*disheveled*, *Wnt7* and *WntA*).

All Wnt pathway genes, except for the *frizzled* receptor subfamilies, were found as single copy genes. The *armadillo* ortholog had very good RNA-seq support, and strongly conserved sequence compared to homologs. This model, however, contains a non-canonical splice site at the 3' end of the fifth exon. Given that the mapped RNA-seq reads strongly support the authenticity of the splice site, this could potentially be a sequencing error. Similarly, the models for both *disheveled* isoforms have a non-canonical splice site at the 3' end of exon 4, with otherwise strong RNA-seq and orthology support for this splice site.

We identified 9 *Wnt* gene subfamilies in the *F. occidentalis* assembly, all with single copy genes: *wingless/Wnt1*, *Wnts* 5-8, 10-11, 16 and *WntA*. *Wnt16* has so far only been reported in the pea aphid *Acyrthosiphon* *pisum* (Shigenobu et al., 2010), the Russian wheat aphid *Diuraphis noxia* (Nicholson et al., 2015) and *Oncopeltus fasciatus*, suggesting that the hemipteroid assemblage (clade Acercaria) has retained a Wnt ligand that was subsequently lost within the Holometabola. Meanwhile, phylogenetic analysis of the protein sequences supports our designation of the *Frankliniella* WntA ortholog, which forms a well-supported clade with WntA proteins from hemipteran species and the beetle *Tribolium castaneum*, with Wnt7 a closely related outgroup. Surprisingly, Wnt4 accessions in GenBank for several species (the hymenopterans *Acromyrmex echinator*, *Harpegnathos saltator*, *Camponotus floridanus*; the termite *Zootermopsis nevadensis*; and the lepidopteran *Papilio xuthus*) also fell within our WntA clade. However, Wnt4 is not known from *Tribolium* or several other well-characterized species such as *Drosophila*. We therefore caution that these Wnt4 accessions were likely wrongly named during automated orthology assignments, and that they in fact represent additional WntA orthologs.

Three *Wnt* models were clustered on scaffold 178, showing the same gene order previously observed in *Tribolium* *castaneum* and *Drosophila melanogaster*: *wingless*- *Wnt6-Wnt10* (Bolognesi et al., 2008). The transcriptional orientation of *Wnt6* was inverted with respect to the other two genes, as is the case in *Tribolium* (Janssen, R. et al., 2010). This gene arrangement still resembles the arrangement of *Wnt* genes observed in *Nematostella*, reflecting the ancient arrangement of *Wnt* genes in metazoans (Sullivan et al., 2007).

The *Wnt* gene repertoire observed in *F. occidentalis* is very similar to the one in *Tribolium* *castaneum* (Bolognesi et al., 2008), both having 9 Wnt subfamilies, with the difference that *Tribolium* lacks *Wnt16*, while *Frankliniella* lacks *Wnt9*. The general trend is for insects to have fewer Wnt subfamilies compared to more basally branching arthropods like the water flea *Daphnia* *pulex* and basal metazoans like *Nematostella vectensis*, with 12 and 13 *Wnt* gene subfamilies, respectively (Janssen, R. et al., 2010).

Of the four ancient *frizzled* (*fz*) receptor subfamilies that are expected to be present in the common ancestor of arthropods (Beermann et al., 2011), we found three in *F. occidentalis*: *frizzled*, three *frizzled*-*2* paralogs, and one *frizzled*-*4*. Two of these models, *frizzled*, and *frizzled-2a*, were split across two scaffolds, but the complete coding sequence could be found. For the third *frizzled* ortholog, *frizzled*-*2c*, the N-terminal region of about 70 amino acids was missing, reflected in a small gap (default size of only 50 bp) in the genome assembly in this region. *fz3* is also missing in *Tribolium castaneum* (Richards et al., 2008) and *Oncopeltus fasciatus* (Panfilio et al., 2019), which have three ( *fz*, *fz2* and *fz4*) and two *fz* families (*fz* and *fz2*), respectively. This would suggest that the loss of *fz3* preceded the split giving rise to Holometabola, but *Drosophila* does have a *fz3* ortholog with highly divergent sequence (Janssen, Ralf et al., 2015), suggesting that “missing” genes are in some cases not recognized due to the rates of molecular evolution relative to the taxonomic sampling currently available.

**Table S8.1.** Positional information for the annotated genes. Incomplete gene models are marked with an asterisk (*).

| **Gene** | **Scaffold: start..end** | **Locus length (nt)** | **Protein length (aa)** | **Number of CDS exons** |
| --- | --- | --- | --- | --- |
| *axin* | Scaffold44:1704986..1711094 | 6,109 | 901 | 11 |
| *armadillo* | Scaffold972:16955..24338 | 7,384 | 817 | 12 |
| *arrow* | Scaffold115:195674..221689 | 26,016 | 1642 | 19 |
| *dishevelled-RA* | Scaffold47:1281711..1294311 | 12,601 | 765 | 15 |
| *dishevelled-RB* | Scaffold47:1281711..1294311 | 12,601 | 625 | 13 |
| *frizzled -part 1 of 2* | Scaffold2979:1031..2872 | 1,842  (partial) | 432  (partial) | 2 |
| *frizzled -part 2 of 2* | Scaffold95:720586..722997 | 2,412  (partial) | 171  (partial) | 1 |
| *frizzled-2a -part 1 of 2* | Scaffold281:127057..127287 | 231  (partial) | 77  (partial) | 1 |
| *frizzled-2a -part 2 of 2* | Scaffold151:643883..646320 | 2,438  (partial) | 628  (partial) | 3 |
| *frizzled-2b* | Scaffold169:473303..478491 | 5,189 | 689 | 4 |
| *frizzled-2c** | Scaffold508:48411..50728 | 2,318  (partial) | 579  (partial) | 3 |
| *frizzled-4* | Scaffold353:41436..50224 | 8,789 | 344 | 2 |
| *shaggy-RA* | Scaffold47:670824..677545 | 6,722 | 504 | 8 |
| *shaggy-RB* | Scaffold47:670824..678020 | 7,197 | 504 | 8 |
| *wingless* | Scaffold178:79250..116897 | 37,648 | 426 | 3 |
| *Wnt5* | Scaffold63:925819..935398 | 9,580 | 292 | 5 |
| *Wnt6* | Scaffold178:68659..69825 | 1,167 | 276 | 4 |
| *Wnt7 -part 1 of 2* | Scaffold2000:1837..5876 | 4,040  (partial) | 84  (partial) | 2 |
| *Wnt7 -part 2 of 2* | Scaffold58:125442..130075 | 4,634  (partial) | 304  (partial) | 3 |
| *Wnt8-RA* | Scaffold22:1034670..1050363 | 15,694 | 402 | 6 |
| *Wnt8-RB* | Scaffold22:1034670..1050363 | 15,694 | 401 | 6 |
| *Wnt10* | Scaffold178:22725..28783 | 6,059 | 468 | 7 |
| *Wnt11* | Scaffold62:66421..68509 | 2,089 | 408 | 4 |
| *Wnt16* | Scaffold8:1141931..1213443 | 71,513 | 420 | 7 |
| *WntA* | Scaffold1:1219341..1266713 | 47,373 | 358 | 6 |
| *wntless* | Scaffold19:514011..515993 | 1,983 | 1088 | 2 |

###

## 8.2 Molting and Metamorphosis

*Contributed by Aaron Baumann*

### 8.2.1. Juvenile hormone esterase (JHE)

Numerous carboxylesterase genes were identified and annotated. Of these, none were given the JH esterase title. BLAST searches using *D. melanogaster* JHE protein sequence pull up 56 putative hits at threshold e<0.00001. Three carboxylesterase annotations meet a “diagnostic” criterion of containing the GQSAG motif characteristic of JH esterase proteins: **12120**, **4777**, and **4770** in which A is replaced with S (GQSSG). It is not unreasonable to propose that any of these could function as a JHE but this notion needs to be experimentally tested. *D. melanogaster* has two JHE proteins, JHE and JHEdup, suggesting independent duplications during insect evolution. It is worth noting that JHEdup contains the motif GHSAG rather than GQSAG.

Phylogenetic reconstruction identifies **13106** and **3184** as putative JHE orthologs.

A sequence identity matrix using trimmed protein sequences (**Fig. S8.1**; top diagonals represent gaps; bottom diagonals represent % identity) suggests greater identity between *D. melanogaster* JHE and **3184** and **12120**. There was more than 45% identity between *D. melanogaster* JHE and 3148 or 12120 vs. ~39% for 4777 and 47770. Likewise, *Rhodnius*, *Glossina*, and *T. castaneum* JHE each share highest sequence identity with **3184** and **12120**, relative to other putative *Frankliniella* JHE proteins. Thus, **3148** and/or **12120** (which also shares the GQSAG motif) seems the likeliest candidate JHE.

**Figure S8.1**. Amino acid identity matrix for putative juvenile hormone esterase sequences (Maker transcript IDs without FOCC prefix) located in the *F. occidentalis* genome.


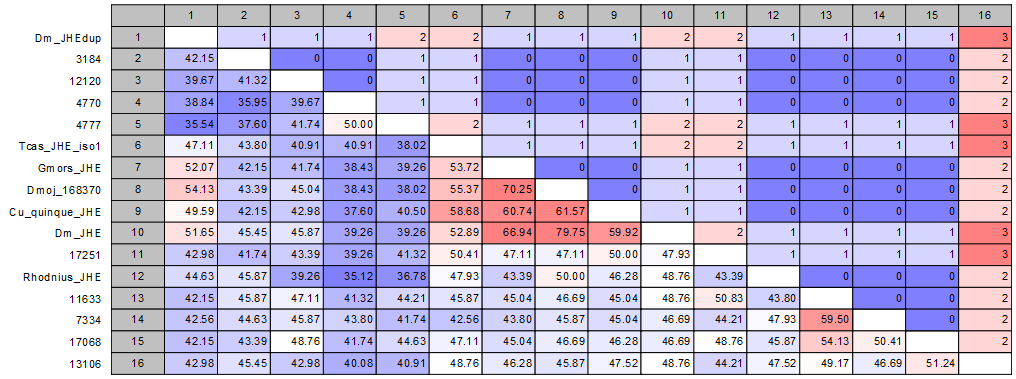


### 8.2.2. bHLH PAS and bHLH Myc family member proteins

In total, 45 orthologs were annotated for the following bHLH-PAS/myc family members (others were already dealt with by other team members, including the E(spl)-bHLH orthologs): 48 related to 3; absent MD neurons and olfactory sensilla (amos); achaete (ac); atonal (ato); atonal-like; clock; clockwork orange (putative); cycle; daughterless; deadpan; dimmed; dimmed-like; dysfusion-like; extra macrochaetae; grainyhead (putative); hairy; hairy (putative dup); Helix Loop Helix Protein 3B; helix-loop-helix protein 11; HLH54F; knot; knot-like (putative); knot-like (putative); max-interacting protein (putative); max-like protein; mitf-like; MLX interacting protein (putative); mnt; Myc; nautilus (putative); Olig family (oli); PAS domain-containing protein; period; scleraxis; similar; single-minded; spineless; Sterol regultory element-binding protein (SREBP/HLH-106); tango (tgo); target of Pox-n (tap); taxi; net; trachealess; twist (putative; tcf15-likehomolog);usf-like1. The **nautilus** annotation; FOCC0016897 may not be complete since this sequence occupies the 5’-most space on the scaffold and there may be additional coding or noncoding exons that were not resolved.

### 8.2.3. bHLH super family protein

***Clock:*** the *Clock* annotation is split across two gene models, FOCC003627 and FOCC003628, which are separated by a run of NNNNN. Names were therefore given as clock (partial) to each model. According to alignments with *Drosophila melanogaster* clock,

I**n addition to several gene losses (or independent gains in more diverged insects), there were several duplication events within this gene super family:**

***E(spl)-bHLH:*** three Enhancer of split paralogs were identified: FOCC004628, 4632,and 4635. A fourth protein, tom (FOCC004629), was included which is also a member of the enhancer of split complex but does not share sequence identity with the E(spl)-bHLH proteins. Shown below are *F. occidentalis* E(spl)-bHLH protein sequences aligned against Drosophila melanogaster E(spl)mBeta-HLH sequence. 4628 and 4632 are likely products of the most recent E(spl)-bHLH duplication in *F. occidentalis*.


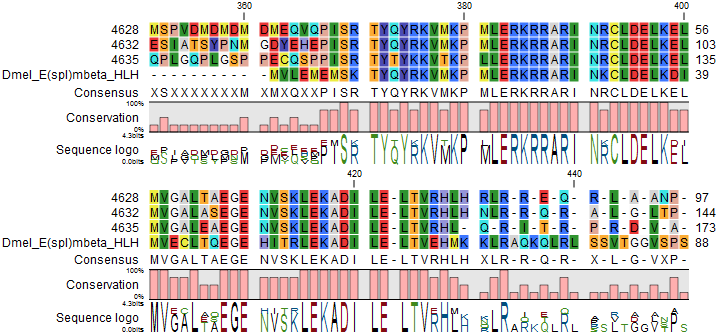


***hairy (h):*** Two *hairy* orthologs were identified, **13163** (8 introns) and **1872** (2 introns). **1872** is likely a retrotransposed copy of **13163**, as evidenced by its paucity of introns, and was thus annotated as “hairy (dup).” A multiple alignment (below) suggests that the *hairy* ortholog in *Drosophila* is likely the direct ancestor to hairy (dup) in *F. occidentalis.*  The ancestral paralog is either lost in Drosophila or annotated with a unique identifier (as is often the case with *Drosophila* paralogs; see *FTZ-F1* and *HR39*, *Met* and *gce*, etc.). The ancestral paralog is thus either lost in *Drosophila* or the homolog is annotated with a unique identifier (such as the case *FTZ-F1* and *HR39* in (Boulanger et al., 2011).


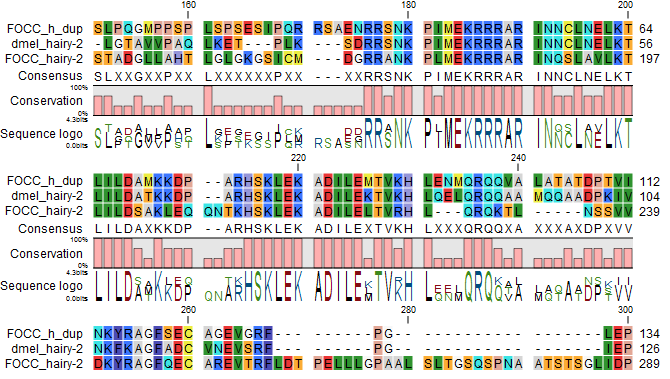


***dimmed:*** two annotations were created that are presumed paralogs of the dimmed bHLH proteins: *dimmed* (14220; 4 introns) and *dimmed-like* (12611; 5 introns). Below shows an alignment of the HLH region of these proteins aligned with *D. melanogaster* dimmed.

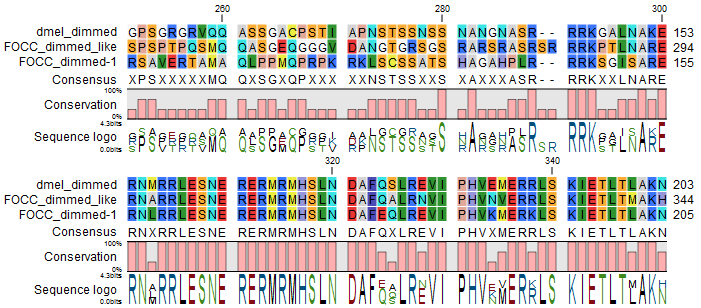


***Knot (syn. collier):*** *knot* (7498, 7499 models merged) and *knot-like* (7501).


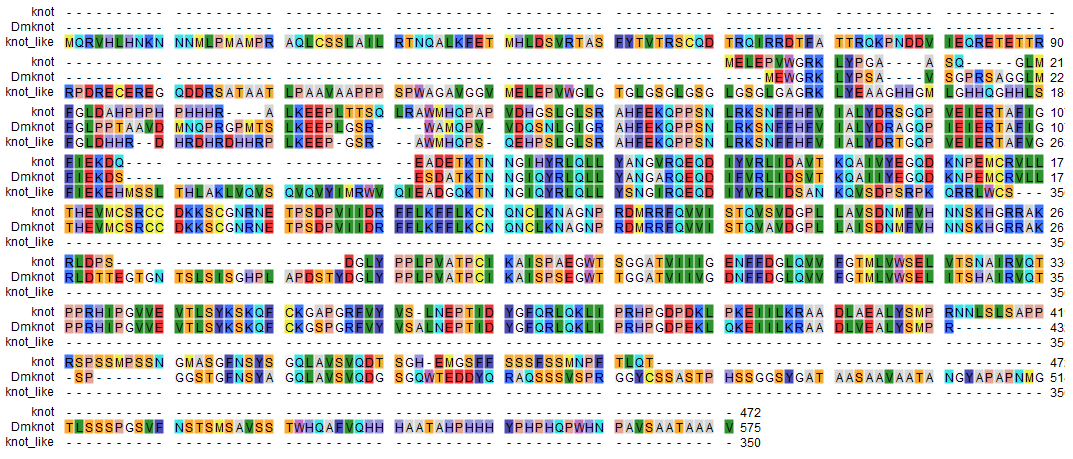


# 9. Cuticular Proteins

*Contributed by Andrew J. Rosendale, Andrew Rosselot, and Joshua Benoit*

## 9.1. Results

Sequence motifs that are characteristic of several families of cuticle proteins (Willis, 2010) were used to search the genome of *Frankliniella occidentalis* for putative cuticle proteins. 101 genes were identified, analyzed with CutProtFam-Pred, a cuticular protein family prediction tool described in Ioannidou et al. (Ioannidou et al., 2014), and assigned to one of 7 families (CPR, CPAP1, CPAP3, CPF, CPCFC, CPLCP, and TWDL) (**Table S9.1**). Many of the genes (~40%) were arranged in clusters of 3 to 5 genes (**Table S9.2**) that were primarily type specific. However, the sizes of gene clusters were smaller than those observed in other insects, which are typically 3 to ~20 genes in size. Additionally, a larger portion (50-70%) of cuticle proteins is typically found in clusters in other insects. Clustering of these genes could allow for the coordinated regulation of cuticle proteins and thereby facilitate the development of insecticide resistance.

As with most insects, the CPR RR-1 (soft cuticle), RR-2 (hard cuticle), and unclassifiable types, constituted the largest group of cuticle protein genes in the *Frankliniella* genome. The number of genes in the protein families CPR, CPAP1, CPAP3, CPCFC, and CPF were similar to the number in other insect (Willis, 2010). However, the 10 genes in the TWDL family was greater than that found in most insect orders, and is reminiscent of the expansion of this family observed in Diptera (**Fig. S9.1**).

| **Table S9.1. Number of genes identified as putative cuticle proteins per family in the genome of *Frankliniella occidentalis*** | | | | | | | | | |
| --- | --- | --- | --- | --- | --- | --- | --- | --- | --- |
| CPR^a^ | | |  |  |  |  |  |  |  |
| RR-1 | RR-2 | Uncl | CPAP1 | CPAP3 | CPF | CPCFC | CPLCP | TWDL | Total |
| 18 | 19 | 27 | 14 | 6 | 3 | 2 | 2 | 10 | 101 |
| ^a^Sequences that scored above the assigned cutoffs for the RR-1 and RR-2 models were classified as the corresponding type, whereas sequences with scores below the assigned cutoffs but above 0 were characterized as “unclassified” (Uncl). For more information, see Ioannidou et al. (Ioannidou et al., 2014). | | | | | | | | | |

| **Table S9.2 Clusters of genes coding cuticle proteins in the genome of *Frankliniella occidentalis*** | | | | | | |
| --- | --- | --- | --- | --- | --- | --- |
|  | **Scaffold #** | **# Genes** | **Family** | **Length (Kbp)** | **Density (Kbp/gene)** |  |
| 1 | 127 | 5 | TWDL | 80 | 15.9 |  |
| 2 | 25 | 5 | CPAP3 | 149 | 29.8 |  |
| 3 | 322 | 5 | CPR RR-1 | 97 | 19.5 |  |
| 4 | 52 | 5 | CPR RR-2 | 111 | 22.3 |  |
| 5 | 111 | 4 | CPR RR-1/CPR Uncl | 108 | 27.0 |  |
| 6 | 6 | 4 | CPR RR-2/CPR Uncl | 47 | 11.6 |  |
| 7 | 13 | 3 | CPR RR-2 | 52 | 17.2 |  |
| 8 | 2 | 3 | CPF | 26 | 8.6 |  |
| 9 | 47 | 3 | TWDL | 31 | 10.4 |  |
| 10 | 94 | 3 | CPAP1 | 155 | 51.5 |  |

**Figure S9.1.** Phylogenetic tree demonstrating relationship of TWDL genes from *Frankliniella occidentalis, Drosophila melanogaster, Tribolium castaneum, Apis mellifera, Pediculus humanus corporis, Acyrthosiphon pisum, Bombyx mori, Cimex lectularis*. *F. occidentalis* showed a greater number of TWDL genes than other insects, with the notable exception of Dipterans such as *D. melanogaster.* The tree was constructed using the neighbor-joining method in MEGA6 with Poisson correction and bootstrap replicates (2,000 replicates).

# REFERENCES CITED

Abdullah, Z.S., Ficken, K.J., Greenfield, B.P., Butt, T.M., 2014. Innate responses to putative ancestral hosts: is the attraction of Western flower thrips to pine pollen a result of relict olfactory receptors?. J.Chem.Ecol. 40, 534-540.

Altincicek, B., Gross, J., Vilcinskas, A., 2008. Wounding-mediated gene expression and accelerated viviparous reproduction of the pea aphid *Acyrthosiphon pisum*. Insect Mol.Biol. 17, 711-716.

Altschul, S.F., Gish, W., Miller, W., Myers, E.W., Lipman, D.J., 1990. Basic local alignment search tool. J. Mol. Biol. 215, 403-410.

Aoki, J., Inoue, A., Makide, K., Saiki, N., Arai, H., 2007. Structure and function of extracellular phospholipase A1 belonging to the pancreatic lipase gene family. 89, 197-204.

Armisén, D., Rajakumar, R., Friedrich, M., Benoit, J.B., Robertson, H.M., Panfilio, K.A., Ahn, S., Poelchau, M.F., Chao, H., Dinh, H., Doddapaneni, H.V., Dugan, S., Gibbs, R.A., Hughes, D.S.T., Han, Y., Lee, S.L., Murali, S.C., Muzny, D.M., Qu, J., Worley, K.C., Munoz-Torres, M., Abouheif, E., Bonneton, F., Chen, T., Chiang, L., Childers, C.P., Cridge, A.G., Crumière, A.J.J., Decaras, A., Didion, E.M., Duncan, E.J., Elpidina, E.N., Favé, M., Finet, C., Jacobs, C.G.C., Cheatle Jarvela, A.M., Jennings, E.C., Jones, J.W., Lesoway, M.P., Lovegrove, M.R., Martynov, A., Oppert, B., Lillico-Ouachour, A., Rajakumar, A., Refki, P.N., Rosendale, A.J., Santos, M.E., Toubiana, W., van, d.Z., Vargas Jentzsch, I.M., Lowman, A.V., Viala, S., Richards, S., Khila, A., 2018. The genome of the water strider *Gerris buenoi* reveals expansions of gene repertoires associated with adaptations to life on the water. 19, 832.

Arp, A.P., Hunter, W.B., Pelz-Stelinski, K., 2016. Annotation of the Asian citrus psyllid genome reveals a reduced innate immune system. Front Physiol. 7, 570.

Badillo-Vargas, I.E., Chen, Y., Martin, K.M., Rotenberg, D., Whitfield, A.E., 2019. Discovery of novel thrips vector proteins that bind to the viral attachment protein of the plant bunyavirus, tomato spotted wilt virus. J.Virol. 93, 699.

Badillo-Vargas, I.E., Rotenberg, D., Schneweis, B.A., Whitfield, A.E., 2015. RNA interference tools for the western flower thrips, *Frankliniella occidentalis*. J.Insect Physiol. 76, 36-46.

Beermann, A., Prühs, R., Lutz, R., Schröder, R., 2011. A context-dependent combination of Wnt receptors controls axis elongation and leg development in a short germ insect. Development. 138, 2793-2805.

Benoit, J.B., Adelman, Z.N., Reinhardt, K., Dolan, A., Poelchau, M., Jennings, E.C., Szuter, E.M., Hagan, R.W., Gujar, H., Shukla, J.N., Zhu, F., Mohan, M., Nelson, D.R., Rosendale, A.J., Derst, C., Resnik, V., Wernig, S., Menegazzi, P., Wegener, C., Peschel, N., Hendershot, J.M., Blenau, W., Predel, R., Johnston, P.R., Ioannidis, P., Waterhouse, R.M., Nauen, R., Schorn, C., Ott, M.C., Maiwald, F., Johnston, J.S., Gondhalekar, A.D., Scharf, M.E., Peterson, B.F., Raje, K.R., Hottel, B.A., Armisen, D., Crumiere, A.J.J., Refki, P.N., Santos, M.E., Sghaier, E., Viala, S., Khila, A., Ahn, S.J., Childers, C., Lee, C.Y., Lin, H., Hughes, D.S.T., Duncan, E.J., Murali, S.C., Qu, J., Dugan, S., Lee, S.L., Chao, H., Dinh, H., Han, Y., Doddapaneni, H., Worley, K.C., Muzny, D.M., Wheeler, D., Panfilio, K.A., Vargas Jentzsch, I.M., Vargo, E.L., Booth, W., Friedrich, M., Weirauch, M.T., Anderson, M.A.E., Jones, J.W., Mittapalli, O., Zhao, C., Zhou, J.J., Evans, J.D., Attardo, G.M., Robertson, H.M., Zdobnov, E.M., Ribeiro, J.M.C., Gibbs, R.A., Werren, J.H., Palli, S.R., Schal, C., Richards, S., 2016. Unique features of a global human ectoparasite identified through sequencing of the bed bug genome. Nat.Commun. 7, 10165.

Benton, R., Vannice, K.S., Gomez-Diaz, C., Vosshall, L.B., 2009. Variant ionotropic glutamate receptors as chemosensory receptors in Drosophila. 136, 149-162.

Bolognesi, R., Farzana, L., Fischer, T.D., Brown, S.J., 2008. Multiple Wnt genes are required for segmentation in the short-germ embryo of *Tribolium castaneum*. Curr.Biol. 18, 1624-1629.

Boonham N, Smith P, Walsh K, Tame J, Morris J, Spence N, Bennison J, Barker I: Detection of *Tomato spotted wilt virus* (TSWV) in individual thrips using real time fluorescent RT-PCR (Taqman). J Virol Meth 2002, 101(1-2):37-48.

Boulanger, A., Clouet-Redt, C., Farge, M., Flandre, A., Guignard, T., Fernando, C., Juge, F., Dura, J.M., 2011. ftz-f1 and Hr39 opposing roles on EcR expression during Drosophila mushroom body neuron remodeling. Nat.Neurosci. 14, 37-44.

Brand, P., Robertson, H.M., Lin, W., Pothula, R., Klingeman, W.E., Jurat-Fuentes, J.L., Johnson, B.R., 2018. The origin of the odorant receptor gene family in insects. Elife. 7, 10.7554/eLife.38340.

Bryon, A., Wybouw, N., Dermauw, W., Tirry, L., Van Leeuwen, T., 2013. Genome wide gene-expression analysis of facultative reproductive diapause in the two-spotted spider mite *Tetranychus urticae*. 14, 815.

Butterwick, J.A., Del Mármol, J., Kim, K.H., Kahlson, M.A., Rogow, J.A., Walz, T., Ruta, V., 2018. Cryo-EM structure of the insect olfactory receptor Orco. 560, 447-452.

Cao, Y., Zhi, J., Cong, C., Margolies, D.C., 2014. Olfactory cues used in host selection by *Frankliniella occidentalis* (Thysanoptera: Thripidae) in relation to host suitability. J.Insect Behav. 27, 41-56.

Capella-Gutiérrez, S., Silla-Martínez, J.M., Gabaldón, T., 2009. trimAl: a tool for automated alignment trimming in large-scale phylogenetic analyses. Bioinformatics. 25, 1972-1973.

Castresana, J., 2000. Selection of conserved blocks from multiple alignments for their use in phylogenetic analysis. Mol.Biol.Evol. 17, 540-552.

Cavodeassi, F., Modolell, J., Gomez-Skarmeta, J.L., 2001. The Iroquois family of genes: from body building to neural patterning. Development. 128, 2847-2855.

Chen, W., Hasegawa, D.K., Kaur, N., Kliot, A., Pinheiro, P.V., Luan, J., Stensmyr, M.C., Zheng, Y., Liu, W., Sun, H., Xu, Y., Luo, Y., Kruse, A., Yang, X., Kontsedalov, S., Lebedev, G., Fisher, T.W., Nelson, D.R., Hunter, W.B., Brown, J.K., Jander, G., Cilia, M., Douglas, A.E., Ghanim, M., Simmons, A.M., Wintermantel, W.M., Ling, K., Fei, Z., 2016. The draft genome of whitefly *Bemisia tabaci* MEAM1, a global crop pest, provides novel insights into virus transmission, host adaptation, and insecticide resistance. 14, 110.

Christiaens, O., Swevers, L., Smagghe, G., 2014. DsRNA degradation in the pea aphid (*Acyrthosiphon pisum*) associated with lack of response in RNAi feeding and injection assay. 53, 307-314.

Chung, H., Sztal, T., Pasricha, S., Sridhar, M., Batterham, P., 2009. Characterization of *Drosophila melanogaster* cytochrome P450 genes. Proc.Natl.Acad.Sci.U.S.A. 106, 5731-5736.

Cifuentes, D., Chynoweth, R., Guillén, J., De La Rúa, P., Bielza, P., 2012. Novel cytochrome P450 genes, CYP6EB1 and CYP6EC1, are over-expressed in acrinathrin-resistant *Frankliniella occidentalis* (Thysanoptera: Thripidae). J Econ Entomol. 105, 1006-1018.

Claudianos, C., Ranson, H., Johnson, R.M., Biswas, S., Schuler, M.A., Berenbaum, M.R., Feyereisen, R., Oakeshott, J.G., 2006. A deficit of detoxification enzymes: pesticide sensitivity and environmental response in the honeybee. Insect Mol.Biol. 15, 615-636.

Cronin, T.W., Porter, M.L., 2014. The Evolution of Invertebrate Photopigments and Photoreceptors, in: Hunt, D.M., Hankins, M.W., Collin, S.P., Marshall, N.J. (Eds.), Evolution of Visual and Non-visual Pigments. Springer US, Boston, MA, pp. 105-135.

Croset, V., Rytz, R., Cummins, S.F., Budd, A., Brawand, D., Kaessmann, H., Gibson, T.J., Benton, R., 2010. Ancient protostome origin of chemosensory ionotropic glutamate receptors and the evolution of insect taste and olfaction. PLoS Genet. 6, e1001064.

Davis, M.M., Engström, Y., 2012. Immune response in the barrier epithelia: lessons from the fruit fly *Drosophila melanogaster*. J.Innate Immun. 4, 273-283.

de Bruijn, P.J., Egas, M., Janssen, A., Sabelis, M.W., 2006. Pheromone-induced priming of a defensive response in Western flower thrips. J.Chem.Ecol. 32, 1599-1603.

de Castro, E., Sigrist, C.J., Gattiker, A., Bulliard, V., Langendijk-Genevaux, P.S., Gasteiger, E., Bairoch, A., Hulo, N., 2006. ScanProsite: detection of PROSITE signature matches and ProRule-associated functional and structural residues in proteins. Nucleic Acids Res. 34, 362.

de Kogel, W.J. and Koschier, E.H, 2002. Thrips responses to plant odours, 189-190.

De Kogel, W.J., Van Deventer, P., 2003. Intraspecific attraction in the western flower thrips, *Frankliniella occidentalis*; indications for a male sex pheromone. Entomol.Exp.Appl. 107, 87-89.

Dermauw, W., Van Leeuwen, T., 2014. The ABC gene family in arthropods: comparative genomics and role in insecticide transport and resistance. Insect Biochem.Mol.Biol. 45, 89-110.

Dermauw, W., Wybouw, N., Rombauts, S., Menten, B., Vontas, J., Grbic, M., Clark, R.M., Feyereisen, R., Van Leeuwen, T., 2013. A link between host plant adaptation and pesticide resistance in the polyphagous spider mite *Tetranychus urticae*. Proc.Natl.Acad.Sci.U.S.A. 110, 113.

Dermauw, W., Osborne, E.J., Clark, R.M., Grbić, M., Tirry, L., Van Leeuwen, T., 2013. A burst of ABC genes in the genome of the polyphagous spider mite *Tetranychus urticae*. 14, 317.

Després, L., David, J.P., Gallet, C., 2007. The evolutionary ecology of insect resistance to plant chemicals. Trends Ecol.Evol. 22, 298-307.

Dörmann, P., 2013. Galactolipids in Plant Membranes.

Dowling, D., Pauli, T., Donath, A., Meusemann, K., Podsiadlowski, L., Petersen, M., Peters, R.S., Mayer, C., Liu, S., Zhou, X., Misof, B., Niehuis, O., 2016. Phylogenetic origin and diversification of RNAi pathway genes in insects. gbe. 8, 3784-3793.

Eriksson, B.J., Fredman, D., Steiner, G., Schmid, A., 2013. Characterisation and localisation of the opsin protein repertoire in the brain and retinas of a spider and an onychophoran. BMC Evol Biol. 13, 186.

Eyun, S.I., Soh, H.Y., Posavi, M., Munro, J.B., Hughes, D.S.T., Murali, S.C., Qu, J., Dugan, S., Lee, S.L., Chao, H., Dinh, H., Han, Y., Doddapaneni, H., Worley, K.C., Muzny, D.M., Park, E.O., Silva, J.C., Gibbs, R.A., Richards, S., Lee, C.E., 2017. Evolutionary history of chemosensory-related gene families across the Arthropoda. Mol.Biol.Evol. 34, 1838-1862.

Facey, P.D., Meric, G., Hitchings, M.D., Pachebat, J.A., Hegarty, M.J., Chen, X., Morgan, L.V.A., Hoeppner, J.E., Whitten, M.M.A., Kirk, W.D.J., Dyson, P.J., Sheppard, S.K., Del Sol, R., 2015. Draft genomes, phylogenetic reconstruction, and comparative genomics of two novel cohabiting bacterial symbionts isolated from *Frankliniella occidentalis*. Genome Biol.Evol. 7, 2188-2202.

Felsenstein, J., 1985. Confidence Limits on Phylogenies: an Approach using the Bootstrap. Evolution. 39, 783-791.

Ferrandon, D., 2013. The complementary facets of epithelial host defenses in the genetic model organism *Drosophila melanogaster*: from resistance to resilience. Curr.Opin.Immunol. 25, 59-70.

Feuda, R., Marlétaz, F., Bentley, M.A., Holland, P.W., 2016. Conservation, duplication, and divergence of five opsin genes in insect evolution. Genome Biol.Evol. 8, 579-587.

Feyereisen, R., 1999. Insect P450 enzymes. Annu.Rev.Entomol. 44, 507-533.

Fujii, S., Yavuz, A., Slone, J., Jagge, C., Song, X., Amrein, H., 2015. Drosophila sugar receptors in sweet taste perception, olfaction, and internal nutrient sensing. Curr.Biol. 25, 621-627.

Ganesan, S., Aggarwal, K., Paquette, N., Silverman, N., 2011. NF-κB/Rel proteins and the humoral immune responses of *Drosophila melanogaster*. Curr.Top.Microbiol.Immunol. 349, 25-60.

Gerardo, N., Altincicek, B., Anselme, C., Atamian, H., Barribeau, S., de Vos, M., Duncan, E., Evans, J., Gabaldon, T., Ghanim, M., Heddi, A., Kaloshian, I., Latorre, A., Moya, A., Nakabachi, A., Parker, B., Perez-Brocal, V., Pignatelli, M., Rahbe, Y., Ramsey, J., Spragg, C., Tamames, J., Tamarit, D., Tamborindeguy, C., Vincent-Monegat, C., Vilcinskas, A., 2010. Immunity and other defenses in pea aphids, *Acyrthosiphon pisum*. Genome Biol. 11, R21.

Guindon, S., Dufayard, J.F., Lefort, V., Anisimova, M., Hordijk, W., Gascuel, O., 2010. New algorithms and methods to estimate maximum-likelihood phylogenies: assessing the performance of PhyML 3.0. Syst.Biol. 59, 307-321.

Guittard, E., Blais, C., Maria, A., Parvy, J.P., Pasricha, S., Lumb, C., Lafont, R., Daborn, P.J., Dauphin-Villemant, C., 2011. CYP18A1, a key enzyme of Drosophila steroid hormone inactivation, is essential for metamorphosis. Dev.Biol. 349, 35-45.

Hamilton, J.G., Hall, D.R., Kirk, W.D., 2005. Identification of a male-produced aggregation pheromone in the western flower thrips *Frankliniella occidentalis*. J.Chem.Ecol. 31, 1369-1379.

Heidel-Fischer, H.M., Vogel, H., 2015. Molecular mechanisms of insect adaptation to plant secondary compounds. Curr Opin Insect Sci. 8, 8-14.

Hering, L., Henze, M.J., Kohler, M., Kelber, A., Bleidorn, C., Leschke, M., Nickel, B., Meyer, M., Kircher, M., Sunnucks, P., Mayer, G., 2012. Opsins in onychophora (velvet worms) suggest a single origin and subsequent diversification of visual pigments in arthropods. Mol.Biol.Evol. 29, 3451-3458.

Hogenhout, S.A., Van der Hoorn, R. A., Terauchi, R., Kamoun, S., 2009. Emerging concepts in effector biology of plant-associated organisms. Mol.Plant Microbe Interact. 22, 115-122.

Höglund, A., Dönnes, P., Blum, T., Adolph, H.W., Kohlbacher, O., 2006. MultiLoc: prediction of protein subcellular localization using N-terminal targeting sequences, sequence motifs and amino acid composition. Bioinformatics. 22, 1158-1165.

Huchard, E., Martinez, M., Alout, H., Douzery, E.J., Lutfalla, G., Berthomieu, A., Berticat, C., Raymond, M., Weill, M., 2006. Acetylcholinesterase genes within the Diptera: takeover and loss in true flies. Proc.Biol.Sci. 273, 2595-2604.

Hull, J.J., Chaney, K., Geib, S.M., Fabrick, J.A., Brent, C.S., Walsh, D., Lavine, L.C., 2014. Transcriptome-based identification of ABC transporters in the western tarnished plant bug *Lygus hesperus*. PLoS One. 9, e113046.

Iga, M., Kataoka, H., 2012. Recent studies on insect hormone metabolic pathways mediated by cytochrome P450 enzymes. Biol.Pharm.Bull. 35, 838-843.

Ioannidis, P., Simao, F.A., Waterhouse, R.M., Manni, M., Seppey, M., Robertson, H.M., Misof, B., Niehuis, O., Zdobnov, E.M., 2017. Genomic features of the damselfly *Calopteryx splendens* representing a sister clade to most insect orders. Genome Biol.Evol. 9, 415-430.

Ioannidou, Z.S., Theodoropoulou, M.C., Papandreou, N.C., Willis, J.H., Hamodrakas, S.J., 2014. CutProtFam-Pred: detection and classification of putative structural cuticular proteins from sequence alone, based on profile hidden Markov models. Insect Biochem.Mol.Biol. 52, 51-59.

Janssen, R., Le Gouar, M., Pechmann, M., Poulin, F., Bolognesi, R., Schwager, E.E., Hopfen, C., Colbourne, J.K., Budd, G.E., Brown, S.J., Prpic, N.M., Kosiol, C., Vervoort, M., Damen, W.G., Balavoine, G., McGregor, A.P., 2010. Conservation, loss, and redeployment of Wnt ligands in protostomes: implications for understanding the evolution of segment formation. BMC Evol.Biol. 10, 374-374.

Janssen, R., Schönauer, A., Weber, M., Turetzek, N., Hogvall, M., Goss, G., Patel, N., McGregor, A., Hilbrant, M., 2015. The evolution and expression of panarthropod frizzled genes. Front Ecol Evol. 3, 96.

Jensen, S.E., 2000. Insecticide resistance in the Western flower thrips, *Frankliniella occidentalis*. Integrated Pest Manag Rev. 5, 131-146.

Johnson, K.P., Dietrich, C.H., Friedrich, F., Beutel, R.G., Wipfler, B., Peters, R.S., Allen, J.M., Petersen, M., Donath, A., Walden, K.K.O., Kozlov, A.M., Podsiadlowski, L., Mayer, C., Meusemann, K., Vasilikopoulos, A., Waterhouse, R.M., Cameron, S.L., Weirauch, C., Swanson, D.R., Percy, D.M., Hardy, N.B., Terry, I., Liu, S., Zhou, X., Misof, B., Robertson, H.M., Yoshizawa, K., 2018. Phylogenomics and the evolution of hemipteroid insects. Proc.Natl.Acad.Sci.USA. 115, 12775.

Josek, T., Walden, K.K.O., Allan, B.F., Alleyne, M., Robertson, H.M., 2018. A foreleg transcriptome for *Ixodes scapularis* ticks: Candidates for chemoreceptors and binding proteins that might be expressed in the sensory Haller's organ. Ticks Tick Borne Dis. 9, 1317-1327.

Joseph, R.M., Carlson, J.R., 2015. Drosophila chemoreceptors: A molecular interface between the chemical world and the brain. Trends Genet. 31, 683-695.

Jung, J.W., Park, K.W., Ahn, Y., Kwon, H.W., 2015. Functional characterization of sugar receptors in the western honeybee, *Apis mellifera*. 18, 19-26.

Käll, L., Krogh, A., Sonnhammer, E.L., 2007. Advantages of combined transmembrane topology and signal peptide prediction--the Phobius web server. Nucleic Acids Res. 35, 429.

Katoh, K., Misawa, K., Kuma, K., Miyata, T., 2002. MAFFT: a novel method for rapid multiple sequence alignment based on fast Fourier transform. Nucleic Acids Res. 30, 3059-3066.

Kent, L.B., Robertson, H.M., 2009. Evolution of the sugar receptors in insects. BMC Evol.Biol. 9, 41-41.

Kingsolver, M.B., Huang, Z., Hardy, R.W., 2013. Insect antiviral innate immunity: Pathways, effectors, and connections. J Mol Biol. 425, 4921-4936.

Kirk, W.D., Hamilton, J.G., 2004. Evidence for a male-produced sex pheromone in the western flower thrips *Frankliniella occidentalis*. J.Chem.Ecol. 30, 167-174.

Kirkness, E.F., Haas, B.J., Sun, W., Braig, H.R., Perotti, M.A., Clark, J.M., Lee, S.H., Robertson, H.M., Kennedy, R.C., Elhaik, E., Gerlach, D., Kriventseva, E.V., Elsik, C.G., Graur, D., Hill, C.A., Veenstra, J.A., Walenz, B., Tubio, J.M., Ribeiro, J.M., Rozas, J., Johnston, J.S., Reese, J.T., Popadic, A., Tojo, M., Raoult, D., Reed, D.L., Tomoyasu, Y., Kraus, E., Mittapalli, O., Margam, V.M., Li, H.M., Meyer, J.M., Johnson, R.M., Romero-Severson, J., Vanzee, J.P., Alvarez-Ponce, D., Vieira, F.G., Aguade, M., Guirao-Rico, S., Anzola, J.M., Yoon, K.S., Strycharz, J.P., Unger, M.F., Christley, S., Lobo, N.F., Seufferheld, M.J., Wang, N., Dasch, G.A., Struchiner, C.J., Madey, G., Hannick, L.I., Bidwell, S., Joardar, V., Caler, E., Shao, R., Barker, S.C., Cameron, S., Bruggner, R.V., Regier, A., Johnson, J., Viswanathan, L., Utterback, T.R., Sutton, G.G., Lawson, D., Waterhouse, R.M., Venter, J.C., Strausberg, R.L., Berenbaum, M.R., Collins, F.H., Zdobnov, E.M., Pittendrigh, B.R., 2010. Genome sequences of the human body louse and its primary endosymbiont provide insights into the permanent parasitic lifestyle. Proc.Natl.Acad.Sci.U.S.A. 107, 12168-12173.

Knecht, Z.A., Silbering, A.F., Ni, L., Klein, M., Budelli, G., Bell, R., Abuin, L., Ferrer, A.J., Samuel, A.D.T., Benton, R., Garrity, P.A., 2016. Distinct combinations of variant ionotropic glutamate receptors mediate thermosensation and hygrosensation in Drosophila. 5, e17879.

Koh, T.W., He, Z., Gorur-Shandilya, S., Menuz, K., Larter, N.K., Stewart, S., Carlson, J.R., 2014. The Drosophila IR20a clade of ionotropic receptors are candidate taste and pheromone receptors. 83, 850-865.

Koschier, E.H., De Kogel, W.J., Visser, J.H., 2000. Assessing the attractiveness of volatile plant compounds to Western flower thrips *Frankliniella occidentalis*. J.Chem.Ecol. 26, 2643-2655.

Krumlauf, R., 1992. Evolution of the vertebrate Hox homeobox genes. Bioessays. 14, 245-252.

Kumar, S., Stecher, G., Li, M., Knyaz, C., Tamura, K., 2018. MEGA X: Molecular evolutionary genetics analysis across computing platforms. Mol.Biol.Evol. 35, 1547-1549.

Larkin, M.A., Blackshields, G., Brown, N.P., Chenna, R., McGettigan, P.A., McWilliam, H., Valentin, F., Wallace, I.M., Wilm, A., Lopez, R., Thompson, J.D., Gibson, T.J., Higgins, D.G., 2007. Clustal W and Clustal X version 2.0. Bioinformatics. 23, 2947-2948.

Lemaitre, B., Hoffmann, J., 2007. The host defense of *Drosophila melanogaster*. Annu.Rev.Immunol. 25, 697-743.

Li B, Dewey CN: RSEM: accurate transcript quantification from RNA-Seq data with or without a reference genome. BMC Bioinformatics 2011, 12:323.

Ligoxygakis, P., 2013. Genetics of immune recognition and response in Drosophila host defense. Adv.Genet. 83, 71-97.

Liscombe, D.K., Louie, G.V., Noel, J.P., 2012. Architectures, mechanisms and molecular evolution of natural product methyltransferases. Nat.Prod.Rep. 29, 1238-1250.

Livak KJ, Schmittgen TD: Analysis of relative gene expression data using real-time quantitative PCR and the 2−ΔΔCT method. Methods 2001, 25(4):402-408.

Mackert, A., do Nascimento, A.M., Bitondi, M.M., Hartfelder, K., Simões, Z.L., 2008. Identification of a juvenile hormone esterase-like gene in the honey bee, *Apis mellifera* L.--expression analysis and functional assays. Comp.Biochem.Physiol.B.Biochem.Mol.Biol. 150, 33-44.

Mainali, B., Lim, U.T., 2011. Behavioral response of Western flower thrips to visual and olfactory cues. J.Insect Behav. 24, 436-446.

McNeill, H., Yang, C.H., Brodsky, M., Ungos, J., Simon, M.A., 1997. Mirror encodes a novel PBX-class homeoprotein that functions in the definition of the dorsal-ventral border in the Drosophila eye. Genes Dev. 11, 1073-1082.

Mesquita, R.D., Vionette-Amaral, R.J., Lowenberger, C., Rivera-Pomar, R., Monteiro, F.A., Minx, P., Spieth, J., Carvalho, A.B., Panzera, F., Lawson, D., Torres, A.Q., Ribeiro, J.M., Sorgine, M.H., Waterhouse, R.M., Montague, M.J., Abad-Franch, F., Alves-Bezerra, M., Amaral, L.R., Araujo, H.M., Araujo, R.N., Aravind, L., Atella, G.C., Azambuja, P., Berni, M., Bittencourt-Cunha, P.R., Braz, G.R., Calderón-Fernández, G., Carareto, C.M., Christensen, M.B., Costa, I.R., Costa, S.G., Dansa, M., Daumas-Filho, C.R., De-Paula, I.F., Dias, F.A., Dimopoulos, G., Emrich, S.J., Esponda-Behrens, N., Fampa, P., Fernandez-Medina, R.D., da Fonseca, R.N., Fontenele, M., Fronick, C., Fulton, L.A., Gandara, A.C., Garcia, E.S., Genta, F.A., Giraldo-Calderón, G.I., Gomes, B., Gondim, K.C., Granzotto, A., Guarneri, A.A., Guigó, R., Harry, M., Hughes, D.S., Jablonka, W., Jacquin-Joly, E., Juárez, M.P., Koerich, L.B., Lange, A.B., Latorre-Estivalis, J.M., Lavore, A., Lawrence, G.G., Lazoski, C., Lazzari, C.R., Lopes, R.R., Lorenzo, M.G., Lugon, M.D., Majerowicz, D., Marcet, P.L., Mariotti, M., Masuda, H., Megy, K., Melo, A.C., Missirlis, F., Mota, T., Noriega, F.G., Nouzova, M., Nunes, R.D., Oliveira, R.L., Oliveira-Silveira, G., Ons, S., Orchard, I., Pagola, L., Paiva-Silva, G.O., Pascual, A., Pavan, M.G., Pedrini, N., Peixoto, A.A., Pereira, M.H., Pike, A., Polycarpo, C., Prosdocimi, F., Ribeiro-Rodrigues, R., Robertson, H.M., Salerno, A.P., Salmon, D., Santesmasses, D., Schama, R., Seabra-Junior, E.S., Silva-Cardoso, L., Silva-Neto, M.A., Souza-Gomes, M., Sterkel, M., Taracena, M.L., Tojo, M., Tu, Z.J., Tubio, J.M., Ursic-Bedoya, R., Venancio, T.M., Walter-Nuno, A.B., Wilson, D., Warren, W.C., Wilson, R.K., Huebner, E., Dotson, E.M., Oliveira, P.L., 2015. Genome of *Rhodnius prolixus*, an insect vector of Chagas disease, reveals unique adaptations to hematophagy and parasite infection. Proc.Natl.Acad.Sci.U.S.A. 112, 14936-14941.

Miller, M.A., Pfeiffer, W., Schwartz, T., 2010. Creating the CIPRES Science Gateway for inference of large phylogenetic trees, 1-8.

Missbach, C., Dweck, H.K., Vogel, H., Vilcinskas, A., Stensmyr, M.C., Hansson, B.S., Grosse-Wilde, E., 2014. Evolution of insect olfactory receptors. Elife. 3, e02115.

Miyamoto, T., Slone, J., Song, X., Amrein, H., 2012. A fructose receptor functions as a nutrient sensor in the Drosophila brain. 151, 1113-1125.

Montero-Astúa M, Ullman DE, and Whitfield AE: Salivary gland morphology, tissue tropism and the progression of tospovirus infection in *Frankliniella occidentalis*. Virology 2016, 493:39-51.

Murat, S., Hopfen, C., McGregor, A.P., 2010. The function and evolution of Wnt genes in arthropods. Arthropod Struct.Dev. 39, 446-452.

Mussabekova, A., Daeffler, L., Imler, J.L., 2017. Innate and intrinsic antiviral immunity in Drosophila. Cell Mol.Life Sci. 74, 2039-2054.

Nelson, D.R., 1998. Cytochrome P450 nomenclature. Methods Mol.Biol. 107, 15-24.

Ni, J.D., Baik, L.S., Holmes, T.C., Montell, C., 2017. A rhodopsin in the brain functions in circadian photoentrainment in Drosophila. 545, 340-344.

Nicholson, S.J., Nickerson, M.L., Dean, M., Song, Y., Hoyt, P.R., Rhee, H., Kim, C., Puterka, G.J., 2015. The genome of *Diuraphis noxia*, a global aphid pest of small grains. BMC Genomics. 16, 429-1.

Nishide, Y., Kageyama, D., Yokoi, K., Jouraku, A., Tanaka, H., Futahashi, R., Fukatsu, T., 2019. Functional crosstalk across IMD and Toll pathways: insight into the evolution of incomplete immune cascades. Proc.Biol.Sci. 286, 20182207.

Oakeshott, J.G., Claudianos, C., Campbell, P.M., Newcomb, R.D., Russell, R.J., 2005. 5.10 - Biochemical Genetics and Genomics of Insect Esterases, in: Gilbert, L.I. (Ed.), Comprehensive Molecular Insect Science. Elsevier, Amsterdam, pp. 309-381.

Oakeshott, J.G., Johnson, R.M., Berenbaum, M.R., Ranson, H., Cristino, A.S., Claudianos, C., 2010. Metabolic enzymes associated with xenobiotic and chemosensory responses in *Nasonia vitripennis*. Insect Mol.Biol. 19 Suppl 1, 147-163.

Oberhofer, G., Grossmann, D., Siemanowski, J.L., Beissbarth, T., Bucher, G., 2014. Wnt/beta-catenin signaling integrates patterning and metabolism of the insect growth zone. Development. 141, 4740-4750.

Olaniran, O.A., Sudhakar, A.V., Drijfhout, F.P., Dublon, I.A., Hall, D.R., Hamilton, J.G., Kirk, W.D., 2013. A male-predominant cuticular hydrocarbon, 7-methyltricosane, is used as a contact pheromone in the western flower thrips *Frankliniella occidentalis*. J.Chem.Ecol. 39, 559-568.

Ozata, D.M., Gainetdinov, I., Zoch, A., O'Carroll, D., Zamore, P.D., 2019. PIWI-interacting RNAs: small RNAs with big functions. Nat.Rev.Genet. 20, 89-108.

Panfilio, K.A., Vargas Jentzsch, I.M., Benoit, J.B., Erezyilmaz, D., Suzuki, Y., Colella, S., Robertson, H.M., Poelchau, M.F., Waterhouse, R.M., Ioannidis, P., Weirauch, M.T., Hughes, D.S.T., Murali, S.C., Werren, J.H., Jacobs, C.G.C., Duncan, E.J., Armisén, D., Vreede, B.M.I., Baa-Puyoulet, P., Berger, C.S., Chang, C., Chao, H., Chen, M.M., Chen, Y., Childers, C.P., Chipman, A.D., Cridge, A.G., Crumière, A.J.J., Dearden, P.K., Didion, E.M., Dinh, H., Doddapaneni, H.V., Dolan, A., Dugan, S., Extavour, C.G., Febvay, G., Friedrich, M., Ginzburg, N., Han, Y., Heger, P., Holmes, C.J., Horn, T., Hsiao, Y., Jennings, E.C., Johnston, J.S., Jones, T.E., Jones, J.W., Khila, A., Koelzer, S., Kovacova, V., Leask, M., Lee, S.L., Lee, C., Lovegrove, M.R., Lu, H., Lu, Y., Moore, P.J., Munoz-Torres, M., Muzny, D.M., Palli, S.R., Parisot, N., Pick, L., Porter, M.L., Qu, J., Refki, P.N., Richter, R., Rivera-Pomar, R., Rosendale, A.J., Roth, S., Sachs, L., Santos, M.E., Seibert, J., Sghaier, E., Shukla, J.N., Stancliffe, R.J., Tidswell, O., Traverso, L., van, d.Z., Viala, S., Worley, K.C., Zdobnov, E.M., Gibbs, R.A., Richards, S., 2019. Molecular evolutionary trends and feeding ecology diversification in the Hemiptera, anchored by the milkweed bug genome. Genome Biol. 20, 64.

Petersen, T.N., Brunak, S., von Heijne, G., Nielsen, H., 2011. SignalP 4.0: discriminating signal peptides from transmembrane regions. Nat.Methods. 8, 785-786.

Poelchau, M., Childers, C., Moore, G., Tsavatapalli, V., Evans, J., Lee, C.Y., Lin, H., Lin, J.W., Hackett, K., 2015. The i5k Workspace@NAL--enabling genomic data access, visualization and curation of arthropod genomes. Nucleic Acids Res. 43, 714.

Porter, M.L., 2016. Beyond the eye: Molecular evolution of extraocular photoreception. Integr.Comp.Biol. 56, 842-852.

Prieto-Godino, L.L., Rytz, R., Cruchet, S., Bargeton, B., Abuin, L., Silbering, A.F., Ruta, V., Dal Peraro, M., Benton, R., 2017. Evolution of acid-sensing olfactory circuits in Drosophilids. 93, 661-676.e6.

Qi, W., Ma, X., He, W., Chen, W., Zou, M., Gurr, G.M., Vasseur, L., You, M., 2016. Characterization and expression profiling of ATP-binding cassette transporter genes in the diamondback moth, *Plutella xylostella* (L.). 17, 760.

Ramsey, J.S., Rider, D.S., Walsh, T.K., De Vos, M., Gordon, K.H., Ponnala, L., Macmil, S.L., Roe, B.A., Jander, G., 2010. Comparative analysis of detoxification enzymes in *Acyrthosiphon pisum* and *Myzus persicae*. Insect Mol.Biol. 19 Suppl 2, 155-164.

Richards, S., Gibbs, R.A., Weinstock, G.M., Brown, S.J., Denell, R., Beeman, R.W., 2008. The genome of the model beetle and pest *Tribolium castaneum*. 452, 955.

Rimal, S., Lee, Y., 2018. The multidimensional ionotropic receptors of *Drosophila melanogaster*. Insect Mol.Biol. 27, 1-7.

Robertson, H.M., 2019. Molecular evolution of the major arthropod chemoreceptor gene families. Annu.Rev.Entomol. 64, 227-242.

Robertson, H.M., 2015. The insect chemoreceptor superfamily is ancient in animals. Chem.Senses. 40, 609-614.

Robertson, H.M., Baits, R.L., Walden, K.K.O., Wada-Katsumata, A., Schal, C., 2018. Enormous expansion of the chemosensory gene repertoire in the omnivorous German cockroach *Blattella germanica*. J.Exp.Zool.B.Mol.Dev.Evol. 330, 265-278.

Robertson, H.M., Kent, L.B., 2009. Evolution of the gene lineage encoding the carbon dioxide receptor in insects. J.Insect Sci. 9, 19.

Robertson, H.M., Warr, C.G., Carlson, J.R., 2003. Molecular evolution of the insect chemoreceptor gene superfamily in *Drosophila melanogaster*. Proc.Natl.Acad.Sci.U.S.A. 100 Suppl 2, 14537-14542.

Rytz, R., Croset, V., Benton, R., 2013. Ionotropic receptors (IRs): chemosensory ionotropic glutamate receptors in Drosophila and beyond. Insect Biochem.Mol.Biol. 43, 888-897.

Saina, M., Busengdal, H., Sinigaglia, C., Petrone, L., Oliveri, P., Rentzsch, F., Benton, R., 2015. A cnidarian homologue of an insect gustatory receptor functions in developmental body patterning. Nat.Commun. 6, 6243.

Saitou, N., Nei, M., 1987. The neighbor-joining method: a new method for reconstructing phylogenetic trees. Mol.Biol.Evol. 4, 406-425.

Sánchez-Alcañiz, J.A., Silbering, A.F., Croset, V., Zappia, G., Sivasubramaniam, A.K., Abuin, L., Sahai, S.Y., Münch, D., Steck, K., Auer, T.O., Cruchet, S., Neagu-Maier, G., Sprecher, S.G., Ribeiro, C., Yapici, N., Benton, R., 2018. An expression atlas of variant ionotropic glutamate receptors identifies a molecular basis of carbonation sensing. Nat Commun. 9, 4252.

Schinkel, A.H., Jonker, J.W., 2012. Mammalian drug efflux transporters of the ATP binding cassette (ABC) family: an overview. Adv.Drug Deliv.Rev. 64, 138-153.

Schneweis, D.J., 2017. Characterizing global gene expression and antiviral response in *Frankliniella occidentalis* infected with Tomato spotted wilt virus, 1-202.

Scott, J.G., Wen, Z., 2001. Cytochromes P450 of insects: the tip of the iceberg. Pest Manag.Sci. 57, 958-967.

Shigenobu, S., Richards, S., Cree, A.G., Morioka, M., Fukatsu, T., Kudo, T., Miyagishima, S., Gibbs, R.A., Stern, D.L., Nakabachi, A., 2010. A full-length cDNA resource for the pea aphid, *Acyrthosiphon pisum*. Insect Mol.Biol. 19 Suppl 2, 23-31.

Sievers, F., Wilm, A., Dineen, D., Gibson, T.J., Karplus, K., Li, W., Lopez, R., McWilliam, H., Remmert, M., Söding, J., Thompson, J.D., Higgins, D.G., 2011. Fast, scalable generation of high-quality protein multiple sequence alignments using Clustal Omega. Mol Syst Biol&nbsp;. 7, 539.

Silva, R., Walter, G.H., Wilson, L.J., Furlong, M.J., 2016. Effect of the postfeeding interval on olfactory responses of thrips to herbivore-induced cotton plants. Insect Sci. 23, 881-892.

Smadja, C., Shi, P., Butlin, R.K., Robertson, H.M., 2009. Large gene family expansions and adaptive evolution for odorant and gustatory receptors in the pea aphid, *Acyrthosiphon pisum*. Mol.Biol.Evol. 26, 2073-2086.

Sparks, M.E., Bansal, R., Benoit, J.B., Blackburn, M.B., Chao, H., Chen, M., Cheng, S., Childers, C., Dinh, H., Doddapaneni, H.V., Dugan, S., Elpidina, E.N., Farrow, D.W., Friedrich, M., Gibbs, R.A., Hall, B., Han, Y., Hardy, R.W., Holmes, C.J., Hughes, D.S.T., Ioannidis, P., Cheatle Jarvela, A.,M., Johnston, J.S., Jones, J.W., Kronmiller, B.A., Kung, F., Lee, S.L., Martynov, A.G., Masterson, P., Maumus, F., Munoz-Torres, M., Murali, S.C., Murphy, T.D., Muzny, D.M., Nelson, D.R., Oppert, B., Panfilio, K.A., Paula, D.P., Pick, L., Poelchau, M.F., Qu, J., Reding, K., Rhoades, J.H., Rhodes, A., Richards, S., Richter, R., Robertson, H.M., Rosendale, A.J., Tu, Z.J., Velamuri, A.S., Waterhouse, R.M., Weirauch, M.T., Wells, J.T., Werren, J.H., Worley, K.C., Zdobnov, E.M., Gundersen-Rindal, D., 2020. Brown marmorated stink bug, *Halyomorpha halys* (Stål), genome: putative underpinnings of polyphagy, insecticide resistance potential and biology of a top worldwide pest. BMC Genomics. 21, 227.

Stamatakis, A., 2014. RAxML version 8: a tool for phylogenetic analysis and post-analysis of large phylogenies. Bioinformatics. 30, 1312-1313.

Stamatakis, A., 2006. RAxML-VI-HPC: maximum likelihood-based phylogenetic analyses with thousands of taxa and mixed models. Bioinformatics. 22, 2688-2690.

Stewart, S., Koh, T.W., Ghosh, A.C., Carlson, J.R., 2015. Candidate ionotropic taste receptors in the Drosophila larva. Proc.Natl.Acad.Sci.U.S.A. 112, 4195-4201.

Sturm, A., Cunningham, P., Dean, M., 2009. The ABC transporter gene family of *Daphnia pulex*. BMC Genomics. 10, 170-170.

Sullivan, J.C., Ryan, J.F., Mullikin, J.C., Finnerty, J.R., 2007. Conserved and novel Wnt clusters in the basal eumetazoan *Nematostella vectensis*. Dev.Genes Evol. 217, 235-239.

Sun, B.F., Xiao, J.H., He, S.M., Liu, L., Murphy, R.W., Huang, D.W., 2013. Multiple ancient horizontal gene transfers and duplications in lepidopteran species. Insect Mol.Biol. 22, 72-87.

Sun, H., Pu, J., Chen, F., Wang, J., Han, Z., 2017. Multiple ATP-binding cassette transporters are involved in insecticide resistance in the small brown planthopper, *Laodelphax striatellus*. Insect Mol.Biol. 26, 343-355.

Swevers, L., Vanden Broeck, J., Smagghe, G., 2013. The possible impact of persistent virus infection on the function of the RNAi machinery in insects: a hypothesis. Front Physiol. 4, 319.

Sztal, T., Chung, H., Berger, S., Currie, P.D., Batterham, P., Daborn, P.J., 2012. A cytochrome p450 conserved in insects is involved in cuticle formation. PLoS One. 7, e36544.

Tamura, K., Stecher, G., Peterson, D., Filipski, A., Kumar, S., 2013. MEGA6: Molecular Evolutionary Genetics Analysis version 6.0. Mol.Biol.Evol. 30, 2725-2729.

Teerling, C.R., Pierce, H.D.,Jr, Borden, J.H., Gillespie, D.R., 1993. Identification and bioactivity of alarm pheromone in the western flower thrips, *Frankliniella occidentalis*. J.Chem.Ecol. 19, 681-697.

Terrapon, N., Li, C., Robertson, H.M., Ji, L., Meng, X., Booth, W., Chen, Z., Childers, C.P., Glastad, K.M., Gokhale, K., Gowin, J., Gronenberg, W., Hermansen, R.A., Hu, H., Hunt, B.G., Huylmans, A.K., Khalil, S.M., Mitchell, R.D., Munoz-Torres, M.C., Mustard, J.A., Pan, H., Reese, J.T., Scharf, M.E., Sun, F., Vogel, H., Xiao, J., Yang, W., Yang, Z., Yang, Z., Zhou, J., Zhu, J., Brent, C.S., Elsik, C.G., Goodisman, M.A., Liberles, D.A., Roe, R.M., Vargo, E.L., Vilcinskas, A., Wang, J., Bornberg-Bauer, E., Korb, J., Zhang, G., Liebig, J., 2014. Molecular traces of alternative social organization in a termite genome. Nat.Commun. 5, 3636.

Teulon, D.A.J., Penman, D.R., Ramakers, P.M.J., 1993. Volatile chemicals for thrips (Thysanoptera: Thripidae) host finding and applications for thrips pest management. J.Econ.Entomol. 86, 1405-1415.

The International Aphid, Genomics Consortium, 2010. Genome sequence of the pea aphid *Acyrthosiphon pisum*. 8, e1000313.

Tian, L., Song, T., He, R., Zeng, Y., Xie, W., Wu, Q., Wang, S., Zhou, X., Zhang, Y., 2017. Genome-wide analysis of ATP-binding cassette (ABC) transporters in the sweetpotato whitefly, *Bemisia tabaci*. 18, 330.

Ullman DE, Cho JJ, Mau RFL, Hunter WB, Westcot DM, Custer DM: Thrips-Tomato spotted wilt virus interactions: morphological, behavioral and cellular components influencing thrips transmission. In *Advances in Disease Vector Research. Volume 9.* Edited by Harris KF. New York: Springer-Verlag; 1992:195-240.

Valenzuela, J.G., Francischetti, I.M., Pham, V.M., Garfield, M.K., Ribeiro, J.M., 2003. Exploring the salivary gland transcriptome and proteome of the *Anopheles stephensi* mosquito. Insect Biochem.Mol.Biol. 33, 717-732.

Velarde, R.A., Sauer, C.D., Walden, K.K., Fahrbach, S.E., Robertson, H.M., 2005. Pteropsin: a vertebrate-like non-visual opsin expressed in the honey bee brain. Insect Biochem.Mol.Biol. 35, 1367-1377.

Vlisidou, I., Wood, W., 2015. Drosophila blood cells and their role in immune responses. FEBS J. 282, 1368-1382.

Wall, D.P., Fraser, H.B., Hirsh, A.E., 2003. Detecting putative orthologs. Bioinformatics. 19, 1710-1711.

Wang, M., 2013. Purification, characterization, and production of β-mannanase from *Bacillus subtilis* TJ-102 and its application in gluco-mannooligosaccharides preparation. Eur Food Res Technol. . 237, 399-408.

Wang H, Reitz S, Wang L, Wang S, Li X, Lei Z: The mRNA expression profiles of five heat shock protein genes from *Frankliniella occidentalis* at different stages and their responses to temperatures and insecticides. J Integr&nbsp; Agric 2014, 13:2196–2210.

Wanker, E., Schörgendorfer, K., Schwab, H., 1991. Expression of the Bacillus subtilis levanase gene in *Escherichia coli* and *Saccharomyces cerevisiae*. J.Biotechnol. 18, 243-254.

Weiss, L.A., Dahanukar, A., Kwon, J.Y., Banerjee, D., Carlson, J.R., 2011. The molecular and cellular basis of bitter taste in Drosophila. 69, 258-272.

Willis, J.H., 2010. Structural cuticular proteins from arthropods: annotation, nomenclature, and sequence characteristics in the genomics era. Insect Biochem.Mol.Biol. 40, 189-204.

Wybouw, N., Pauchet, Y., Heckel, D.G., Van Leeuwen, T., 2016. Horizontal gene transfer contributes to the evolution of arthropod herbivory. Genome Biol.Evol. 8, 1785-1801.

193. Xie W, Yang X, Chen C, Yang Z, Guo L, Wang D, Huang J, Zhang H, Wen Y, Zhao J, Wu Q, Wang S, Coates BS, Zhou X, Zhang Y: The invasive MED/Q *Bemisia tabaci* genome: a tale of gene loss and gene gain. BMC Genomics 2018, 19(1):68.

Yan, D.K., Hu, M., Tang, Y.X., Fan, J.Q., 2015. Proteomic analysis reveals resistance mechanism against chlorpyrifos in *Frankliniella occidentalis* (Thysanoptera: Thripidae). J.Econ.Entomol. 108, 2000-2008.

Yu, Q.Y., Lu, C., Li, W.L., Xiang, Z.H., Zhang, Z., 2009. Annotation and expression of carboxylesterases in the silkworm, *Bombyx mori*. BMC Genomics. 10, 553-553.

Zhang, C., Zhang, S., Xia, J., Li, F., Xia, W., Liu, S., Wang, X., 2014. The immune strategy and stress response of the Mediterranean species of the *Bemisia tabaci* complex to an orally delivered bacterial pathogen. 9, e94477.

Zhu, F., Moural, T.W., Shah, K., Palli, S.R., 2013. Integrated analysis of cytochrome P450 gene superfamily in the red flour beetle, *Tribolium castaneum*. BMC Genomics. 14, 174-174.

Zuckerkandl, E., Pauling, L., 1965. Evolutionary divergence and convergence in proteins, in: Bryson, V., Vogel, H.J. (Eds.), Evolving Genes and Proteins. Academic Press, pp. 97-166.
